# The Involvement of Xanthone and (*E*)-Cinnamoyl Chromophores for the Design and Synthesis of Novel Sunscreening Agents

**DOI:** 10.3390/ijms22010034

**Published:** 2020-12-22

**Authors:** Justyna Popiół, Agnieszka Gunia-Krzyżak, Karolina Słoczyńska, Paulina Koczurkiewicz-Adamczyk, Kamil Piska, Katarzyna Wójcik-Pszczoła, Dorota Żelaszczyk, Anna Krupa, Paweł Żmudzki, Henryk Marona, Elżbieta Pękala

**Affiliations:** 1 Department of Pharmaceutical Biochemistry, Faculty of Pharmacy, Jagiellonian University Medical College, Medyczna 9, 30-688 Kraków, Poland; justyna.popiol@uj.edu.pl (J.P.); karolina.sloczynska@uj.edu.pl (K.S.); paulina.koczurkiewicz@uj.edu.pl (P.K.-A.); kamil.piska@uj.edu.pl (K.P.); katarzynaanna.wojcik@uj.edu.pl (K.W.-P.); elzbieta.pekala@uj.edu.pl (E.P.); 2 Department of Bioorganic Chemistry, Chair of Organic Chemistry, Faculty of Pharmacy, Jagiellonian University Medical College, Medyczna 9, 30-688 Kraków, Poland; dorota.zelaszczyk@uj.edu.pl (D.Ż.); henryk.marona@uj.edu.pl (H.M.); 3 Department of Pharmaceutical Technology and Biopharmaceutics, Faculty of Pharmacy, Jagiellonian University Medical College, Medyczna 9, 30-688 Kraków, Poland; a.krupa@uj.edu.pl; 4 Department of Medicinal Chemistry, Faculty of Pharmacy, Jagiellonian University Medical College, Medyczna 9, 30-688 Kraków, Poland; pawel.zmudzki@uj.edu.pl

**Keywords:** UV absorption, UV filters, UV radiation, photoprotective activity, xanthone, (*E*)-cinnamoyl

## Abstract

Excessive UV exposure contributes to several pathological conditions like skin burns, erythema, premature skin aging, photodermatoses, immunosuppression, and skin carcinogenesis. Effective protection from UV radiation may be achieved with the use of sunscreens containing UV filters. Currently used UV filters are characterized by some limitations including systemic absorption, endocrine disruption, skin allergy induction, and cytotoxicity. In the research centers all over the world new molecules are developed to improve the safety, photostability, solubility, and absorption profile of new derivatives. In our study, we designed and synthesized seventeen novel molecules by combining in the structures two chromophores: xanthone and (*E*)-cinnamoyl moiety. The ultraviolet spectroscopic properties of the tested compounds were confirmed in chloroform solutions. They acted as UVB or UVA/UVB absorbers. The most promising compound 9 (6-methoxy-9-oxo-9*H*-xanthen-2-yl)methyl (*E*)-3-(2,4-dimethoxyphenyl)acrylate) absorbed UV radiation in the range 290–369 nm. Its photoprotective activity and functional photostability were further evaluated after wet milling and incorporation in the cream base. This tested formulation with compound 9 possessed very beneficial UV protection parameters (SPF_in vitro_ of 19.69 ± 0.46 and UVA PF of 12.64 ± 0.32) which were similar as broad-spectrum UV filter tris-biphenyl triazine. Additionally, compound 9 was characterized by high values of critical wavelength (381 nm) and UVA/UVB ratio (0.830) thus it was a good candidate for broad-spectrum UV filter and it might protect skin against UVA-induced photoaging. Compound 9 were also shown to be photostable, non-cytotoxic at concentrations up to 50 µM when tested on five cell lines, and non-mutagenic in Ames test. It also possessed no estrogenic activity, according to the results of MCF-7 breast cancer model. Additionally, its favorable lipophilicity (miLogP = 5.62) does not predispose it to penetrate across the skin after topical application.

## 1. Introduction

Ultraviolet radiation (UVR) detected on Earth significantly affects all living organisms including humans. Apart from positive effects of the reasonable UVR exposure, e.g., vitamin D production, several pathological conditions like skin burns, erythema, premature skin aging, photodermatoses, immunosuppression, and skin carcinogenesis were identified as results of extensive UVR exposure [1,2,3]. In order to provide effective protection from UV radiation, sunscreen preparations containing UV filters are recommended. Currently used UV filters are characterized by many drawbacks such as interrupting of endocrine system, skin allergic reactions, and cytotoxicity [2,4,5,6]. Further attempts are made all over the world to discover novel molecules which would provide effective UV protection with minimum adverse side effects.

One of the strategies in the development of new UV filter molecules is the modification of well-known sunscreens by means of multiplying the chromophore and by incorporation of additional auxochromes or alkyl groups. These modifications are intended to improve safety, photostability, solubility, and absorption profile of new derivatives. For example, the molecule of ethylhexyl triazone approved in the late 1990s contains three molecules of PABA (para-aminobenzoic acid) linked to a triazine ring. The chromophore used in triplicate allowed to obtain broad absorption profile and high molecular weight that prevent penetration through the skin [7,8]. Bino et al. focused on the modification of 2-phenyl-1*H*-benzimidazole-5-sulfonic acid by incorporating additional functional groups in phenyl and benzimidazole rings to obtain molecules able to act as UV absorbers and inhibitors of radical species formation [9]. In our recent study, we designed new UV filters based on the structure of 3-benzylidene camphor. In our compounds, the camphor fragment was replaced with the imidazolidine-2,4-dione moiety with the aim of improving safety, the incorporation of allyl group contributed to the extension of the UV absorption, whereas 2-ethoxy-2-oxoethyl substituents in the imidazolidine ring improved the solubility of molecules [10].

**Figure 1:**
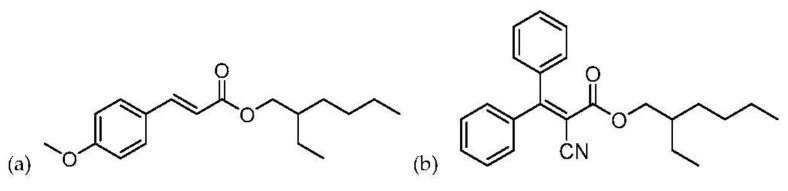
The chemical structures of octinoxate (INCI: Ethylhexyl methoxycinnamate) (**a**) and octocrylene (**b**).

Cinnamates are widely used in cosmetics, not only as UV filters or UV absorbers but also as skin and hair conditioners, antioxidants, perfuming, masking, and antimicrobial ingredients [11]. UV filters that are (*E*)-cinnamoyl derivatives have been approved in selected countries all over the world: octinoxate (INCI: *Ethylhexyl methoxycinnamate*, EHMC), amiloxate (INCI: *Isoamyl p-methoxycinnamate*), octocrylene, cinoxate, diethanolamine methoxycinnamate, 2,5-diisopropyl methyl cinnamate, and isopentyl trimethoxycinnamate trisiloxane [12,13]. Octinoxate (Figure 1a) is ethylhexyl ester of 4-methoxycinnamic acid, its structure allows electron delocalization required for absorbance in UVB region and is typical for UV filters—constitutes disubstituted aromatic molecule with electron releasing (-OCH_3_) and electron accepting (ester group) substituents. Electron releasing group is in para position to the carbonyl group which is additionally conjugated with a double bond [14]. Octocrylene (Figure 1b) is ethylhexyl ester of 2-cyano-3,3-diphenylacrylic acid, this compound is characterized by relatively low extinction coefficient but has an excellent photostability and is used as photostabilizer of other UV filters [15]. Absorption range of cinnamates is narrow, they provide protection only in UVB region, moreover EHMC upon irradiation undergoes trans-cis isomerization which results in absorption loss [16]. Therefore, the aim of our study was to design new potential UV filters by modifications of (*E*)-cinnamic acid derivatives to obtain new, safe, broad absorbing and photostable compounds. We decided to combine (*E*)-cinnamoyl moiety with another chromophore—xanthone skeleton.

**Figure 2:**
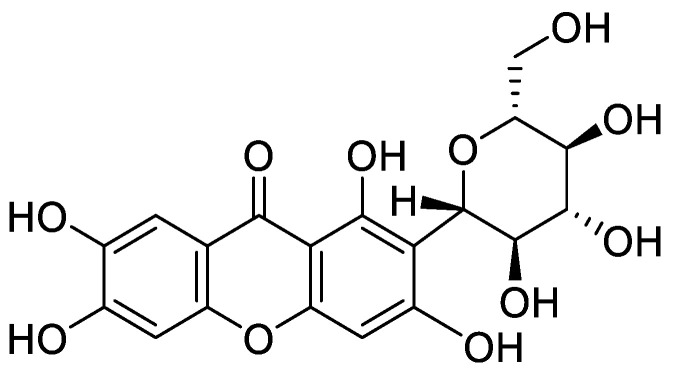
The chemical structure of mangiferin.

Xanthone (dibenzo-γ-pyrone) possesses conjugated double bond system and several potential substitution positions. Coupling with an appropriate auxochromic groups and additional chromophores gives the opportunity to select substances not only with the intended photoprotectiveness in a wide UV range but also with a satisfactory safety profile. Xanthone derivatives have been subject of multidirectional research for many years. A lot of them have a favorable biological and pharmacological activity profile, including among other antioxidant [17], antimicrobial [18], anticancer [19,20], and anti-inflammatory effect [21]. Moreover, xanthone derivatives have also recently been of interest in the field of cosmetics chemistry. Mangiferin (2-β-D-glucopyranosyl-1,3,6,7-tetrahydroxy-9*H*-xanthen-9-one, Figure 2) and its derivatives are of particular interest, its cosmetic application is the subject of many patents claims [22]. Moreover in in vivo study mangiferin was shown to inhibit UVB-induced length and depth of skin wrinkles. Additionally, mangiferin improves the photostability of avobenzone and reduces its phototoxic potential [23,24].

**Figure 3:**
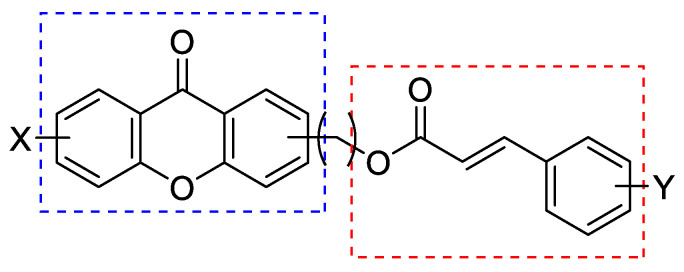
The design concept of the novel UV filters: combination of xanthone and (*E*)-cinnamoyl chromophores.

In the present study, we designed and synthesized novel derivatives combining in the structures the two above mentioned chromophores: xanthone and (*E*)-cinnamoyl moiety (Figure 3). We intended to obtain compounds characterized by lipophilicity predisposing for affinity to stratum corneum rather than penetrating into deeper layers of the skin. Apart from UV absorption properties, we aimed to incorporate the most promising compounds into cosmetic formulation to evaluate their photoprotective activity and photostability in the conditions of future use. Additionally, with in vitro methods we assessed their safety profile in mutagenic and estrogenic activity assays. We also performed tests of their cytotoxicity in keratinocytes and skin fibroblasts as well as in hepatocytes, astrocytes and cardiomyoblasts.

## 2. Results and Discussion

### 2.1. Chemistry

**Scheme 1:**
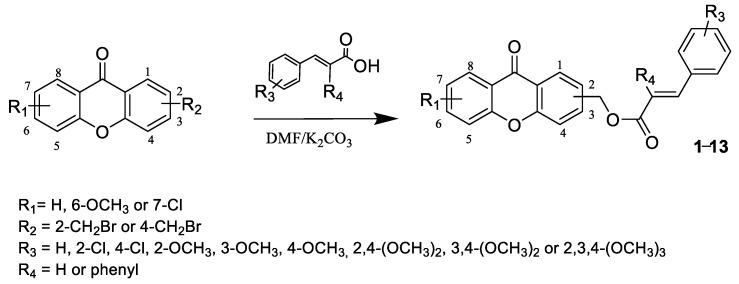
General route of synthesis of compounds **1**–**13**.

**Scheme 2:**
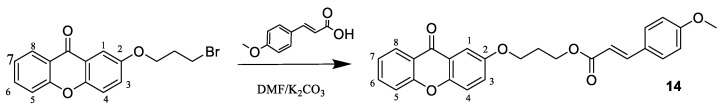
General route of synthesis of compound **14**.

**Scheme 3:**
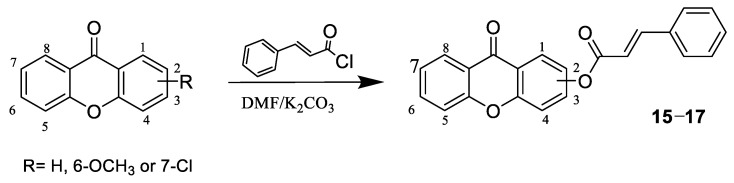
General route of synthesis of compounds **15**–**17**.

Chemical structures of tested compounds were shown in Table 1 and the route of their synthesis was presented in Scheme 1, Scheme 2 and Scheme 3.

Synthesis of starting materials for preparation of compounds **1**–**13**, i.e., 4-(bromomethyl)-9*H*-xanthen-9-one [25], 2-(bromomethyl)-6-methoxy-9*H*-xanthen-9-one [26], 5-(bromomethyl)-3-methoxy-9*H*-xanthen-9-one [19] and 2-(bromomethyl)-7-chloro-9*H*-xanthen-9-one [27] was performed according to previously published procedures. These compounds were further used in the reaction with appropriate derivatives of (*E*)-cinnamic acid in DMF in the presence of anhydrous K_2_CO_3_ as a proton acceptor resulting in obtaining of compounds **1**–**13** (Scheme 1). Substrates for synthesis of compounds **14**–**17,** i.e., 2-, 3-, or 4-hydroxy-9*H*-xanthen-9-one [28,29,30,31] and 2-(3-bromopropoxy)-9*H*-xanthen-9-one [32] were also synthesized according to available literature. 2-(3-Bromopropoxy)-9*H*-xanthen-9-one was further used in the reaction with (*E*)-4-methoxycinnamic acid to synthesize compound **14** (Scheme 2). 2-, 3-, or 4-hydroxy-9*H*-xanthen-9-ones were used in the reaction with (*E*)-cinnamoyl chloride to synthesize compounds **15**–**17** (Scheme 3). Chemical structures and purity of final compounds were confirmed by spectral analysis (NMR, LC/MS/MS).

### 2.2. Lipophilicity Evaluation

The relative lipophilicity parameters (R_M0_) of the tested compounds were determined using reversed-phase thin-layer chromatography (RP-TLC) according to previously published procedures [33,34]. The investigations were carried on RP-18-coated aluminum sheets with mobile phase consisting of diverse concentrations of methanol and potassium phosphate buffer (pH = 7.4). The experimental data showed a linear correlation between R_M_ values and the concentration of methanol, the obtained correlation coefficients ranged from 0.956 to 0.996 (Table S2 Supplementary Materials). The relative lipophilicity values (R_M0_) of the tested compounds, calculated as extrapolations of R_M_ for 0% methanol concentrations, ranged from 4.19 to 6.15 (Table 1). We also calculated LogP values by Molispiration on-line tool [35] and provided results in Table 1. The calculated values of miLogP ranged from 5.21 to 7.10.

Previous studies showed that lipophilicity was an important parameter of the molecule affecting the possibility of skin permeation. Compounds with logP of 4–5 were characterized by poor permeation rate across the skin, while compounds with more hydrophilic nature (logP = 0–2.5) were predisposed to effective skin permeation after topical application [36]. Similarly, according to formal guidance for the testing of cosmetic ingredients, substances with logP ≥ 4 were characterized by very low dermal absorption [37]. Based on the performed lipophilicity evaluation, reported here UV filters would not penetrate across the skin after topical application.

### 2.3. Ultraviolet Spectroscopic Properties

**Figure 4:**
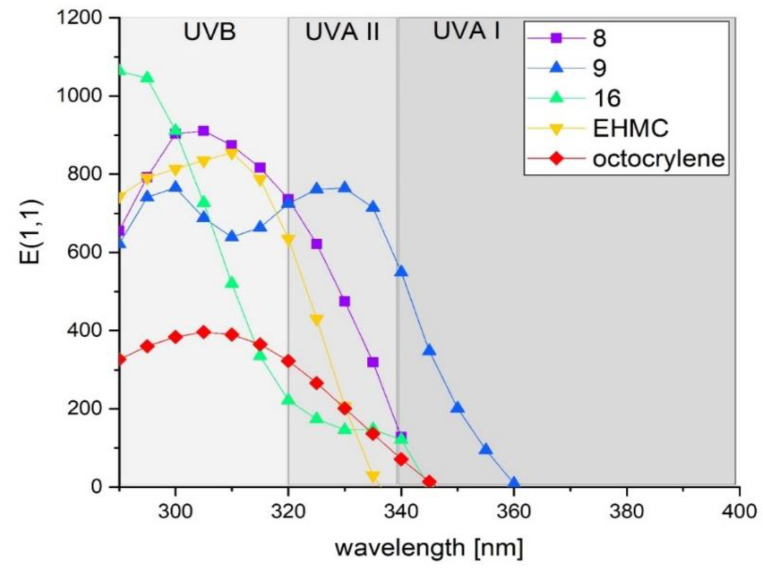
UV-absorption spectra of tested compounds and reference UV filters in chloroform solutions. E(1,1)—extinction at 1% concentration and 1 cm optical pathlength.

The results of studies on the ultraviolet spectroscopic properties of the tested compounds and reference UV filters were presented in Table 2. This type of screening using low-concentrated compound solutions is very useful in the initial phase of searching for new UV filter candidates and is aimed at identification of structures for more advanced tests. Among presented group of (*E*)-cinnamoyl xanthone derivatives, all tested compounds might be characterized as ultraviolet radiation absorbers. UV spectroscopic properties of some of them surpass commercially available UV filters such us 3-(4-methyl)benzylidene camphor (4-MBC), octocrylene (OCT), octinoxate (EHMC) and avobenzone (BMDM). Compound **1** showed the weakest absorption properties. This compound is deprived of 6-methoxy group in xanthone skeleton additionally chloro substituent is located in ortho position of phenyl ring of *trans*-cinnamoyl residue. Compound **2** with chlorine atom in para position was characterized by much higher molar extinction coefficient in comparison to the compound **1**, whereas compound **4** with both chlorine atom in para position and 6-methoxy group in xanthone skeleton showed not only higher ε_max_ and ‹*E*_1,1_›_mean_ value than compounds **1**–**3** but also bathochromic shift by 6 nm. Ultraviolet spectroscopic properties of compounds **7** and **8** were very similar which indicated that position of methoxycinnamoyl substituent in xanthone ring did not significantly affect their UV absorption properties. The λ_max_ of these compounds (300 and 303 nm, respectively), similarly to EHMC, was located in UVB region, additionally their ε_max_, ‹*E*_1,1_›_mean_ and absorption range were much higher in comparison to the reference UVB filters (Figure 4). E(1,1) value as well as molecular mass are often used to present UV filters properties. Reported here (*E*)-cinnamoyl xanthone derivatives possessed high molar mass, which is desirable from modern UV filters, therefore despite much higher ε_max_ for compounds **7** and **8** in comparison to the EHMC and 4-MBC, their E(1,1) values were similar.

Absorption spectra of compounds **9** and **10** with two methoxy groups in phenyl ring of cinnamoyl residue respectively in 2,4- and 3,4- position differed from other derivatives in the tested group demonstrated two peaks of λ_max_ one in UVB and one in UVA II region, additionally the values of ε_max_ were equally high at both wavelengths which was well reflected in the high ‹*E*_1,1_›_mean_ value, especially for compound **9**. Ultraviolet absorption curve of compound **9** was presented in Figure 4.

Compounds **15**–**17** without alkyl or alkoxy linker between xanthone moiety and (*E*)-cinnamoyl residue showed different ultraviolet spectroscopic properties than other compounds. The higher values of molar extinction coefficient were observed at 290 nm moreover E(1,1) value for compounds **15** (1115) and **16** (1064) with methyl cinnamate substituent in position 2- or 3- of xanthone ring were comparable to avobenzone. Despite high E(1,1) values, these compounds were characterized by relatively low ‹*E*_1,1_›_mean_ parameters. This was due to the fact that the absorbance was very high at the initial wavelengths but gradually decreased as the wavelength increased (Figure 4). Compound **16** might be considered as a strong UVB absorber.

### 2.4. Photoprotective Activity

The most promising ultraviolet spectroscopic properties showed (*E*)-cinnamoyl derivatives of 6-metoxy-2-methyl-9*H*-xanthen-9-one, compounds **8** and **9**. They were subjected to further tests of photoprotective activity. These compounds were poorly soluble in common solvents used in cosmetic products, such as ethanol, polysorbates, triacetin, glycerol, or polyoxyethylene glycols. Thus, the selection of an appropriate solvent that would enable us to achieve the desired concentration of the UV filter in the final formulation, turned out to be impossible. This prompted us to search for a more advanced technological approach to incorporate these insoluble compounds into a cosmetic formulation. For that reason, the tested compounds were wet milled in a high-energy ball milling process. The milling procedure was carried out in the presence of triacetin, and finally a concentrated paste of each of the tested compounds was formed. This paste could be easily combined with the cream base.

Finally, the formulations loaded with 12.5% (*w*/*w*) of compounds **8** or **9** were tested using an in vitro method, recommended for the assessment of photoprotective parameters in cosmetics products [38]. The absorbance of the samples spread onto PMMA (polymethylmethacrylate) plates with a rough surface, imitating the physical characteristic of the skin was measured with the SPF-290AS Analyzer. This device was equipped with an integrating sphere which collects light scattered by the sample.

**Figure 5:**
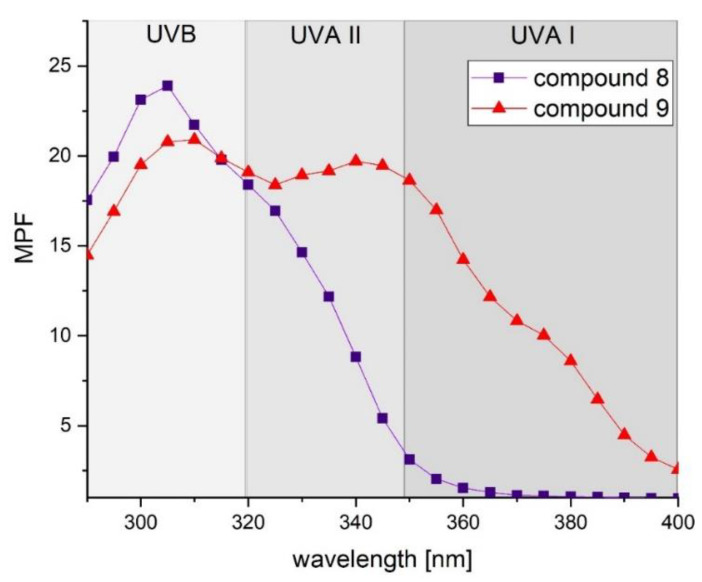
UV absorption spectra of compounds **8** and **9** obtained in cosmetic formulation at 12.5% (*w*/*w*) cosmetic formulation applied on polymethylmethacrylate plates. MPF—monochromatic protection factor.

Further studies aimed to compare the photoprotective activity of the compounds **8** and **9**. The results were presented in Table 3 and Figure 5. Compound **8** ((*E*)-4-methoxy-cinnamoyl xanthone derivative) showed high absorption of UVB radiation. The value of SPF_in vitro_ of a 12.5% formulation was 14.69 ± 2.25 and exceeded the SPF_in vitro_ coefficient, which can be achieved at the maximum allowable concentration by a number of UV protective substances, such as octocrylene, octinoxate, enzacamene, ensulizole, or iscotrizinol [39]. Moreover compound **8** was also active in UVA II region, UVA PF reached 4.92 ± 0.78.

Compound **9** possessed an additional methoxy group in 2- position of phenyl ring of (*E*)-cinnamoyl residue. This additional auxochrome caused significant changes in UV absorption properties as compared to compound **8**. Compound **9** in a 12.5% formulation showed SPF_in vitro_ coefficient of 19.69 ± 0.46 and UVA PF of 12.64 ± 0.32. These values were similar to tris-biphenyl triazine, which is a broad-spectrum UV-filter and was approved for the use in cosmetic products within the European Union in 2014. Couteau et al. tested this compound with a similar in vitro method using 50 mg of the formulation instead of 32 mg applied to the PMMA plate in the present study. The obtained values of SPF_in vitro_ and UVA-PF were respectively 21.85 ± 2.70 and 9.64 ± 0.75 [40].

Differences between compound **8** and **9** were also found in two other important parameters that characterize sunscreen formulations such as critical wavelength (λ*_c_*) and UVA/UVB ratio. Critical wavelength is the wavelength for which the section under the integrated optical density curve starting at 290 nm is equal to 90% of the integrated section between 290 to 400 nm [41] and is in the range from 320 nm which means that UVA radiation is not absorbed to 389 nm when UVA radiation is absorbed to the same extent as UVB [42]. Critical wavelength and UVA/UVB ratio in final sunscreen product should be respectively at least 370 nm and 1/3 (0.33333) to provide a minimum UVB and UVA protection. For compound **8** critical wavelength and UVA/UVB ratio were 345 nm and 0.329, respectively, which indicated that it provided little protection against UVA radiation and could be considered as a UVB filter. For compound **9** these parameters were 381 nm and 0.830, respectively. These findings indicated that compound **9** was a good candidate for broad-spectrum UV filter and provided sufficient spectrum of sunscreen product (required in EU) without other UV filters in formulation [41]. Additionally, compound **9** might protect skin against UVA-induced photoaging, because its critical wavelength exceeded 380 [43].

### 2.5. Photostability Studies

**Figure 6:**
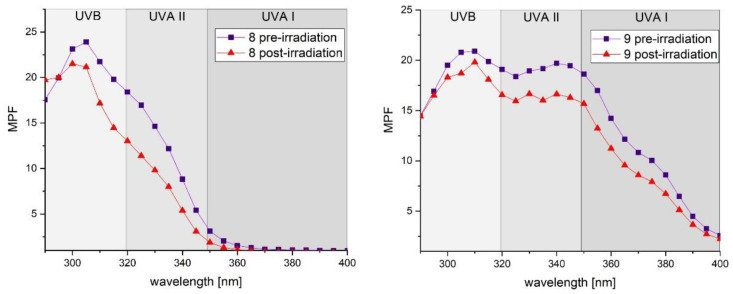
UV absorption spectra of compounds **8** and **9** obtained in pre-irradiation conditions and 1 h post-irradiation with solar light simulator conducted at 500 W/m^2^ in 12.5% (*w*/*w*) cosmetic formulations.

In order to evaluate functional photostability of compounds **8** and **9**, thin layer of formulations containing tested compounds applied on PMMA plates were irradiated with solar light simulator. The changes in SPF_in vitro_, UVA PF, λ*_c_* and UVA/UVB ratio following 1 h irradiation corresponding to natural sunlight were investigated. Results were presented in Figure 6 and Table 4. The irradiation of formulation with compound **8** caused the change in the shape of absorption curve-slight hypsochromic shift and hypochromic effect were observed. The decrease of SPF_in vitro_ and UVA PF by 23.95 and 32.8%, respectively, was observed. The value of critical wavelength shifted by 10 nm from 345 nm to 335 nm, additionally the UVA/UVB ratio decreased from 0.339 to 0.201. It indicated that irradiation mainly contributed to decrease in protection in the UVA region. The decrease of SPF_in vitro_ was comparable to that showed by EHMC. In previous study, formulation containing EHMC was irradiated in the same conditions and % of Initial SPF_in vitro_ was 80.83 [10].

Compound **9** was characterized by much higher photostability. None hypsochromic shift was observed, the value of critical wavelength changed only by 1 nm. The decrease in SPF_in vitro_ and UVA PF was 10.97 and 17.25%, respectively. Based on the fact that the photoprotective activity parameters did decrease by more than 20%, compound **9** might be considered as a photostable UV filter [44,45]. It is worth mentioning that for avobenzone which is most widely used UVA absorber in Europe, USA, Australia, and Japan the decrease of UVA PF in cosmetic formulation reaches 41% [46]. Thus, compound **9** may be a good alternative for avobenzone.

The changes in photoprotective activity upon irradiation of compounds **8** and **9** most likely resulted from the photoisomerization process. Both compounds, similarly to EHMC, are (*E*)-cinnamate derivatives. The main photochemical reaction for these compounds is E-Z (trans-cis) isomerization. This photoreaction takes place upon irradiation of UV filters containing unsaturated bonds and is considered to be a very efficient way of dispersing the absorbed energy [16,47]. In the case of EHMC, both isomers form a photostationary state which results in UV absorption loss (30–50%) because the resulting cis isomer of EHMC has almost twice lower extinction coefficient [16]. Compound **9** in comparison to the **8**, was characterized by higher photostability. It probably was results of the presence of an additional methoxy group in ortho position of phenyl ring.

### 2.6. Safety Assessment

Compound **9**, due to its broad-spectrum UV absorbance and excellent photostability, and compound **16**, a strong UVB absorber with the most promising properties within the group of compounds with methyl cinnamate substituent were subjected to in vitro preliminary safety assessment. The cytotoxicity of **9** and **16** was evaluated using two cell lines of human origin: keratinocytes (HaCaT) and skin fibroblasts (BJ). Additionally, hepatocytotoxicity, neurocytotoxicity and cardiocytotoxicity were tested. As reference standards, four commercially available UV filters were used including cinnamic acid derivatives (octocrylene (OCT) and octinoxate (EHMC)) as well as 3-(4-methyl)benzylidene camphor (4-MBC), and benzophenone-1 (BP-1). To investigate the metabolic activity of cells incubated in the presence of tested compounds, a MTT (3-(4,5-dimethylthiazol-2-yl)-2,5-diphenyltetrazolium bromide) assay was performed. Additionally, estrogenic activity of compound **9** was examined in preliminary indirect model involving MCF-7 breast cancer cell line, while UV filter benzophenone-2 (BP-2) was used as positive control. Additionally, mutagenicity of compounds **8**, **9**, and **10** was evaluated in vitro using microplate Ames assay.

#### 2.6.1. Cytotoxicity Assessment

The cytotoxicity of compounds **9** and **16** was tested on five cell lines: human keratinocytes, human skin fibroblast, human liver cancer, rat cardiomyoblast and mouse astrocytes. The two cell lines delivered from human skin cells: keratinocytes and fibroblasts were chosen based on the fact that skin cells are mostly exposed to topically applied cosmetic formulations. However, some known UV-filters (e.g., octinoxate and benzophenone-3) were proved to cross skin barrier and penetrate to systemic blood stream after topical application [5] so we used further cell lines: human liver cancer, rat cardiomyoblast, and mouse astrocytes to investigate potential systemic hepatocytotoxicity, cardiocytotoxicity, and neurocytotoxicity of the tested compounds, respectively.

**Figure 7:**
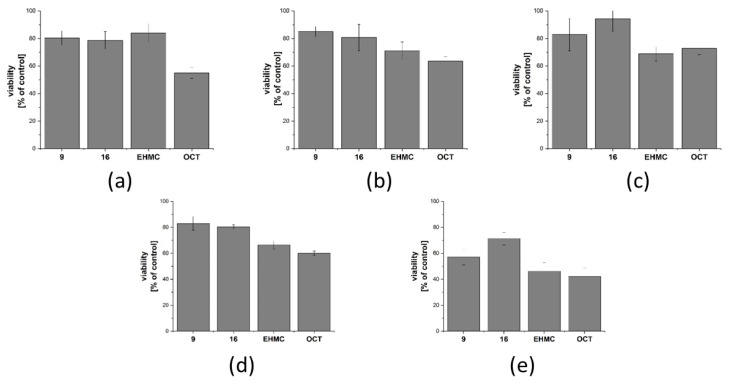
Viability of human keratinocytes HaCaT (**a**) and human skin fibroblast (BJ) (**b**) HepG2 cells (**c**), mouse astrocytes (**d**) rat cardiomyoblasts H9c2 (**e**) incubated for 24 h in the presence of compounds **9** and **16**, EHMC, and OCT at 50 µM. Cell viability was determined using MTT assay. Each experiment was repeated in triplicate. Graphs represent the number of viable cells expressed as percent of control (cells not treated with compounds) ± SD.

The viability of two different types of skin cells grown in presence of the highest administered concentration was presented in Figure 7a,b. The cytotoxicity was examined after 24 h of incubation with compounds **9** and **16** and reference UV filters (EHMC, OCT). Results of cytotoxicity study on human keratinocytes indicated that compounds **9** and **16**, and EHMC in the range of tested concentration (2–50 µM) were deprived of cytotoxic activity. A viability of HaCaT cells at the highest tested concentration (50 µM) was above 78%. In contrast, the viability of human keratinocytes incubated in the presence of reference filters significantly decreased (less than 55%). A similar dependence was observed also in BJ fibroblast model, where compounds **9** and **16** did not significantly reduce cell viability (>80% of control), while incubation with EHMC and OCT resulted in 70.90% and 63.47% viable cells, respectively.

The results obtained after incubation of HepG2 cells, mouse astrocytes and rat cardiomyoblasts with the compounds **9** and **16**, octinoxate, and octocrylene at the highest administered (50 µM) concentration were presented in Figure 7c–e. Results of cytotoxicity on HepG2 hepatocellular cells indicated that compounds **9** and **16** were safe in the range of tested concentrations—viability of cells at 50 µM concentration was 82.79% and 94.26%, respectively. Viability of HepG2 cells incubated with EHMC and OCT was 68.87% and 72.75%, respectively. Compounds **9** and **16** were also deprived of cytotoxic activity against mouse astrocytes, the viability of cells was higher than 80%. However reference control decreased cellular viability to 59%. Results obtained after incubation of rat cardiomyoblasts with compounds **9** and **16** at 50 µM concentration indicated cell viability at the level of 57.02% and 71.25%, respectively, for EHMC and OCT viability was 46.25% and 42.12%, respectively.

#### 2.6.2. In Vitro Effect on MCF-7 Cells—Preliminary Indirect Model of Estrogenic Activity

**Figure 8:**
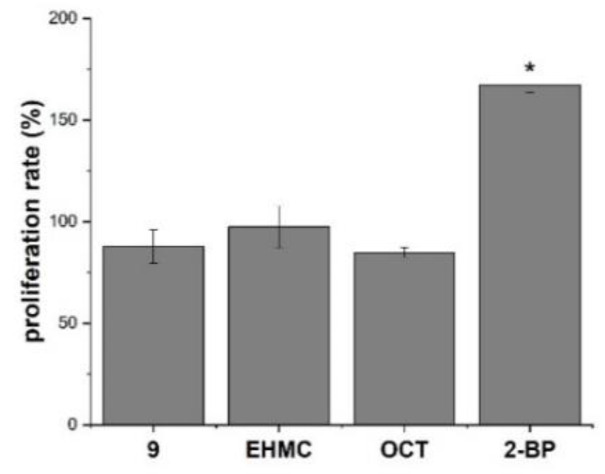
Proliferation rate (±SD) of MCF-7 cells incubated with tested compounds at 5 µM. Compound **9**, EHMC, and OCT showed lack of proliferation-stimulating effect. BP-2 increased MCF-7 proliferation by over 60% (*—data significantly different from control, *p* < 0.05).

Altering of normal function of endocrine system may lead to a variety of health problems including reproductive impairments as well as female and male cancers. Some currently used UV filters (e.g., benzophenones and benzylidene camphor derivatives) were proved to influence estrogenic system [48]. Thus, we used preliminary indirect model involving MCF-7 breast cancer cell line to investigate estrogenic activity of compound **9**. In this indirect model, potential estrogenic activity may be concluded based on the observation of increased cell proliferation after binding of the tested compound with the estrogen receptor [48]. We also examined some commercially available UV filters (EHMC, OCT, and BP-2). The obtained results were presented in Figure 8. Proliferation analyses indicated lack of stimulation effect of tested compounds in MCF-7 breast cancer model. In contrast, UV filter BP-2, stimulated proliferation by over 60%, which was statistically significant. This preliminary evaluation indicated lack of estrogenic activity of compound **9**.

#### 2.6.3. Mutagenic Activity

Mutagenic activity is one of the most important endpoints for risk assessment of chemical compounds including drugs and candidate drugs. In the present study, mutagenicity of test compounds **8** and **9** was evaluated in vitro using microplate Ames assay. Microplate format mutagenicity assay showed that test compounds **8** and **9** did not induce more than a twofold induction over the baseline or a dose dependent response, demonstrating the absence of mutagenic activity (Table 5).

### 2.7. Structure-Activity Relationship Studies

(*E*)-cinnamoyl xanthone derivatives absorbed UV radiation in chloroform solutions in the range of 290–375 nm with λ_max_ 290–329, depending on the position of (*E*)-cinnamoyl moiety in xanthone skeleton and on the presence, type and position of auxochrome in both xanthone and cinnamoyl moiety. The presence of methoxy group in position 6 of xanthone ring contributed to significant improvement of the ultraviolet spectroscopic properties, compounds **1** and **2** without 6-methoxy substituent showed worse UV absorption potential when compared to their 6-methoxy analogues, compounds **3** and **4**. It seemed more favorable to introduce a methoxy group than a chlorine atom into the phenyl ring of the cinnamoyl residue because it did not contribute to the increase of extinction coefficient, but the higher values of ‹*E*_1,1_›_mean_ was observed for compounds **5** and **7** than **3** and **4**. The effect of the presence of two methoxy groups in the phenyl ring of the cinnamoyl residue was very favorable—it contributed to the appearance of additional λ_max_ as well as extension of the UV absorption range into the UVA region and increase of ‹*E*_1,1_›_mean_ value. Moreover, an additional methoxy group in “ortho” position caused improvement of photoprotective activity and functional photostability (compound **8** vs. **9**). Introduction of further methoxygroup in meta position did not result in improvement of UV absorption properties (compounds **10** and **11**). In compounds **1**–**13** the position of (*E*)-cinnamoyl substituent in xanthone skeleton did not affect ultraviolet absorption properties. On the other hand, in compounds **15**–**17** the influence of the position of (*E*)-cinnamoyl substituent was significant. Based on the obtained values of extinction coefficient and ‹*E*_1,1_›_mean_, the most favorable was substitution in position 3 of xanthone ring (compound **16**).

## 3. Materials and Methods

### 3.1. Chemistry

All reagents used in synthesis of compounds were commercially available of at least 97% purity. Solvents were commercially available materials of reagent grade. Melting points (mp) were uncorrected and were determined using a Buchi SMP-20 apparatus (Buchi Labortechnik, Flawil, Switzerland). The ^1^HNMR (300 MHz) were obtained in CDCl_3_ with a Varian Mercury-VX 300 NMR spectrometer (Varian Inc., Palo Alto, CA, USA) using TMS as an internal standard. Spectral data includes chemical shifts in ppm, multiplicities, constant couplings in Hz, number of protons, protons’ positions. Multiplicities are abbreviated as follow: s (singlet), d (doublet), dd (doublet of doublets), ddd (double doublet of doublets), ddt (double doublet of triplets), t (triplet), quint (quintet), and m (multiplet). The LC/MS system consisted of an Acquity UPLC system (Waters Corporation, Milford, MA, USA) coupled to a Waters TQD mass spectrometer (electrospray ionization mode ESI-tandem quadrupole). All the LC/MS analyses were carried out using an Acquity UPLC BEH C18, 1.7 µm, 2.1 × 100 mm^2^ column. A flow rate of 0.3 mL/min and a gradient of (5–95)% B over 10 min and then 100% B over 2 min was used. Eluent A: water/0.1% HCOOH; eluent B: acetonitrile/0.1% HCOOH. LC/MS data were obtained by scanning the first quadrupole in 0.5 s in a mass range from 50 to 1000 Da; 8 scans were added to produce the final spectrum.

#### 3.1.1. Preparation of Substrates for Syntheses

Substrates used in the synthesis of compounds **1**–**17** were obtained according to previously published procedures. Their physicochemical data was consistent with available literature. The first step was the modified Ullmann’s condensation [49] of the commercially available substrates, i.e., appropriate sodium salts of 2-chlorobenzoic acid reacted with cresol, 2- or 4-methoxyphenol or phenol (also as sodium salts). The condensation was performed at the temperature of 200–210 °C in paraffin oil with the catalytic amount of Cu and Cu2O. Then by cyclization (2 to 4 h) of condensation products using concentrated sulfuric acid obtained a corresponding 9*H*-xanthen-9-ones: 4-methyl-9*H*-xanthen-9-one [25,50], 6-chloro- or 7-chloro-2-methyl-9*H*-xanthen-9-one [27], 3-chloro-5-methyl-9*H*-xanthen-9-one [19], 3-chloro-9*H*-xanthen-9-one [51]. 2- and 4-Hydroxy-9*H*-xanthen-9-one [28,31] were products of demethylation in cyclization process. 3-Methoxy- [30], 6-methoxy-2-methyl-9*H*-xanthen-9-one [52] and 3-methoxy-5-methyl-9*H*-xanthen-9-one [19] were obtained by methanolysis of appropriate 3-chloro and 6-chloro substituted 9*H*-xanthen-9-ones. Then appropriate methyl derivatives of 9*H*-xanthen-9-one were subject to bromination with N-bromosuccinimide in the presence of benzoyl peroxide catalyst in tetrachloride [19,25,26]. 3-Hydroxy-9*H*-xanthen-9-one was obtained by demethylation of 3-metoxy-9*H*-xanthen-9-one using 80% H_2_SO_4_ [53]. 2-(3-Bromopropoxy)-9*H*-xanthen-9-one was prepared in the two-step reaction from 2-hydroxy-9*H*-xanthen-9-one using 3-chloropropanol and phosphorus tribromide [32] (Schemes S1 and S2 Supplementary Materials).

#### 3.1.2. Preparation of Compounds **1**–**13**

A mixture of appropriate bromo-substituted xanthone derivative (0.01 mol), appropriate (*E*)-cinnamic acid derivative (0.011 mol), and potassium carbonate (0.007 mol) was stirred in DMF (20 mL) for 8 to 10 h at room temperature. Then the mixture was poured into ice and obtained solid was filtered and washed with 5% sodium bicarbonate solution and water. After drying crude product was crystallized from toluene: heptan (2/1) with the addition of silica gel. The yield of reaction was 64 to 76%.

#### 3.1.3. Preparation of Compound **14**

A mixture of 2-(3-bromopropoxy)-9*H*-xanthen-9-one (0.01 mol), trans-4-methoxycinnamic acid (0.011 mol), and potassium carbonate (0.007 mole was stirred in DMF (20 mL) for 8 to 10 h at room temperature. Then the mixture was poured into ice and obtained solid was filtered and washed with 5% sodium bicarbonate solution and water. After drying crude product was crystallized from toluene: heptan (2/1) with the addition of silica gel. The yield of reaction was 63%.

#### 3.1.4. Preparation of Compounds **15**–**17**

A mixture of appropriate 2-, 3- or 4-hydroxy-9*H*-xanthen-9-one derivative (0.01 mol), 0.015 mole of (*E*)-cinnamoyl chloride (0.015 mol), and potassium carbonate (0.007 mol) was stirred in DMF (20 mL) for 6 to 8 h at room temperature. Then the mixture was poured into ice and obtained solid was filtered and washed with 5% sodium bicarbonate solution and water. After drying crude product was crystallized from toluene: heptan (2/1) with the addition of silica gel. The yield of reaction was 64 to 70%.

#### 3.1.5. Physiochemical Properties of Tested Compounds

(9-oxo-9*H*-xanthen-4-yl)methyl (*E*)-3-(2-chlorophenyl)acrylate (**1**) was obtained as white solid (yield 64%), MW = 390.82, mp 156–157 °C, ^1^H NMR (300 MHz, CDCl_3_) δ 8.40–8.30 (m, 2H), Ar-H1, Ar-H8, 8.20 (dd, *J* = 16.0, 0.7 Hz, 1H, =CH-), 7.86 (ddd, *J* = 7.3, 1.7, 0.8 Hz, 1H, Ar-H3), 7.76 (ddd, *J* = 8.7, 7.1, 1.8 Hz, 1H, Ar-H6), 7.67–7.52 (m, 2H, Ar-H7, Cyn-H6), 7.51–7.35 (m, 3H, Ar-H2, Cyn-H3, Cyn-H4), 7.35–7.20 (m, 2H, Ar-H5, Cyn-H5), 6.52 (d, *J* = 16.0 Hz, 1H, -CH=), 5.67 (s, 2H, -CH_2_-), ESI-MS (*m*/*z*): [M + H]+ calcd. for C_23_H_15_ClO_4_, 391.82, found, 391.11 100%.

(9-oxo-9*H*-xanthen-4-yl)methyl (*E*)-3-(4-chlorophenyl)acrylate (**2**) was obtained as white solid (yield 66%), MW = 390.82, mp 177–178 °C, ^1^H NMR (300 MHz, CDCl_3_) δ 8.34 (dt, *J* = 8.0, 1.6 Hz, 2H, Cyn-H2, Cyn-H6), 7.83 (dd, *J* = 7.3, 1.7 Hz, 1H, Ar-H8), 7.79–7.61 (m, 2H, Ar-H1, =CH-), 7.54 (dd, *J* = 8.5, 1.1 Hz, 1H, Ar-H3), 7.50–7.26 (m, 6H, Ar-H7, Ar-H6, Ar-H5, Ar-H2, Cyn-H3, Cyn-H5), 6.49 (d, *J* = 16.0 Hz, 1H, -CH=), 5.63 (s, 2H, -CH_2_-), ESI-MS (*m*/*z*): [M + H]+ calcd. for C_23_H_15_ClO_4_, 391.82, found, 391.11 98.46%.

(6-methoxy-9-oxo-9*H*-xanthen-4-yl)methyl (*E*)-3-(2-chlorophenyl)acrylate (**3**) was obtained as white solid (yield 69%), MW = 420.85, mp 160–162 °C, ^1^H NMR (300 MHz, CDCl_3_) δ 8.32 (dd, *J* = 8.0, 1.8 Hz, 1H, Ar-H1), 8.27–8.10 (m, 2H, =CH-, Ar-H7), 7.81 (ddd, *J* = 7.3, 1.7, 0.8 Hz, 1H, Ar-H3), 7.66–7.56 (m, 1H, Ar-H8), 7.48–7.19 (m, 4H, Ar-H2, Cyn-H3, Cyn-H4, Cyn-H5), 7.00–6.89 (m, 2H, Ar-H5, Cyn-H6), 6.52 (d, *J* = 16.0 Hz, 1H, -CH=), 5.64 (s, 2H, -CH_2_-), 3.94 (s, 3H, -OCH_3_), ESI-MS (*m*/*z*): [M + H]+ calcd. for C_24_H_17_ClO_5_, 421.85, found, 421.15 98.02%.

(6-methoxy-9-oxo-9*H*-xanthen-4-yl)methyl (*E*)-3-(4-chlorophenyl)acrylate (**4**) was obtained as white solid (yield 66%), MW = 420.85, mp 202–203 °C, ^1^H NMR (300 MHz, CDCl_3_) δ 8.33 (dd, *J* = 8.0, 1.8 Hz, 1H, Ar-H1), 8.25 (d, *J* = 8.8 Hz, 1H, Cyn-H5), 7.81 (dd, *J* = 7.4, 1.8 Hz, 1H, Ar-H2), 7.72 (d, *J* = 16.0 Hz, 1H, =CH-), 7.46 (d, *J* = 8.5 Hz, 2H, Cyn-H2, Cyn-H6), 7.40 (d, *J* = 7.6 Hz, 1H, Ar-H8), 7.36 (d, *J* = 3.1 Hz, 1H, Cyn-H3), 7.34 (s, 1H, Ar-H5), 6.98 (d, *J* = 2.4 Hz, 1H, Ar-H7), 6.96–6.91 (m, 1H, Ar-H3), 6.50 (d, *J* = 16.0 Hz, 1H, -CH=), 5.63 (s, 2H, -CH_2_-), 3.94 (s, 3H, -OCH_3_), ESI-MS (*m*/*z*): [M + H]+ calcd. for C_24_H_17_ClO_5_, 421.85, found, 421.15 100%.

(6-methoxy-9-oxo-9*H*-xanthen-4-yl)methyl (*E*)-3-(2-methoxyphenyl)acrylate (**5**) was obtained as white solid (yield 68%), MW = 416.43, mp 164–165 °C, ^1^H NMR (300 MHz, CDCl_3_) δ 8.32 (dd, *J* = 8.0, 1.7 Hz, 1H, Ar-H1), 8.27–8.21 (m, 1H, Ar-H8), 8.09 (d, *J* = 16.2 Hz, 1H, =CH-), 7.85–7.79 (m, 1H, Cyn-H6), 7.51 (dd, *J* = 7.7, 1.7 Hz, 1H, Ar-H3), 7.42–7.31 (m, 2H, Ar-H2, Cyn-H4), 6.99–6.88 (m, 4H, Ar-H7, Ar-H5, Cyn-H3, Cyn-H5), 6.62 (d, *J* = 16.1 Hz, 1H, -CH=), 5.62 (s, 2H, -CH_2_-), 3.93 (s, 3H, -OCH_3_), 3.87 (s, 3H, -OCH_3_), ESI-MS (*m*/*z*): [M + H]+ calcd. for C_25_H_20_O_6_, 417.43, found, 417.16 100%.

(6-methoxy-9-oxo-9*H*-xanthen-4-yl)methyl (*E*)-3-(3-methoxyphenyl)acrylate (**6**) was obtained as white solid (yield 67%), MW = 416.43, mp 153–154 °C, ^1^H NMR (300 MHz, CDCl_3_) δ 8.32 (dd, *J* = 8.0, 1.8 Hz, 1H, Ar-H1), 8.31–8.18 (m, 1H, Ar-H8), 7.81 (ddd, *J* = 7.4, 1.6, 0.8 Hz, 1H, Ar-H3), 7.74 (d, *J* = 16.0 Hz, 1H, =CH-), 7.44–7.33 (m, 1H, Cyn-H4), 7.33–7.22 (m, 1H, Ar-H2), 7.12 (ddt, *J* = 7.6, 1.5, 0.7 Hz, 1H, Cyn-H6), 7.04–6.88 (m, 4H, Ar-H5, Ar-H7, Cyn-H2, Cyn-H5), 6.51 (d, *J* = 16.0 Hz, 1H, -CH=), 5.62 (s, 2H, -CH_2_-), 3.93 (s, 3H, -OCH_3_), 3.81 (s, 3H, -OCH3), ESI-MS (*m*/*z*): [M + H]+ calcd. for C_25_H_20_O_6_, 417.43, found, 417.09 100%.

(6-methoxy-9-oxo-9*H*-xanthen-4-yl)methyl (*E*)-3-(4-methoxyphenyl)acrylate (**7**) was obtained as white solid (yield 71%), MW = 416.43, mp 167–168 °C, ^1^H NMR (300 MHz, CDCl_3_) δ 8.33 (dd, *J* = 8.0, 1.7 Hz, 1H, Ar-H1), 8.25 (dd, *J* = 8.6, 0.7 Hz, 1H, Ar-H8), 7.82 (dd, *J* = 7.4, 1.8 Hz, 1H, Ar-H7), 7.74 (d, *J* = 16.0 Hz, 1H, =CH-), 7.49 (d, *J* = 8.8 Hz, 2H, Cyn-H2, Cyn-H6), 7.39 (t, *J* = 7.7 Hz, 1H, Ar-H2), 6.98 (d, *J* = 2.4 Hz, 1H, Ar-H3), 6.95 (s, 1H, Ar-H5), 6.90 (d, *J* = 8.8 Hz, 2H, Cyn-H3, Cyn-H5), 6.40 (d, *J* = 15.9 Hz, 1H, -CH=), 5.62 (s, 2H, -CH_2_-), 3.94 (s, 3H, -OCH_3_), 3.83 (s, 3H, -OCH_3_), ESI-MS (*m*/*z*): [M + H]+ calcd. for C_25_H_20_O_6_, 417.43, found, 417.20 100%.

(6-methoxy-9-oxo-9*H*-xanthen-2-yl)methyl (*E*)-3-(4-methoxyphenyl)acrylate (**8**) was obtained as white solid (yield 71%), MW = 416.43, mp 169–171 °C, ^1^H NMR (300 MHz, CDCl_3_) δ 8.38–8.33 (m, 1H, Ar-H1), 8.24 (d, *J* = 8.9 Hz, 1H, Ar-H8), 7.77–7.72 (m, 1H, Ar-H3), 7.69 (d, *J* = 15.9 Hz, 1H, =CH-), 7.51–7.43 (m, 3H, Ar-H4, Cyn-H6, Cyn-H2), 6.96 (d, *J* = 2.4 Hz, 1H, Ar-H5), 6.93 (d, *J* = 2.4 Hz, 1H, Ar-H7), 6.91–6.83 (m, 2H, Cyn-H3, Cyn-H5), 6.40–6.32 (m, 1H, -CH=), 5.33 (s, 2H, -CH_2_-), 3.93 (s, 3H, -OCH_3_), 3.82 (s, 3H, -OCH_3_), ESI-MS (*m*/*z*): [M + H]+ calcd. for C_25_H_20_O_6_, 417.43, found, 417.23 99.14%.

(6-methoxy-9-oxo-9*H*-xanthen-2-yl)methyl (*E*)-3-(2,4-dimethoxyphenyl)acrylate (**9**) was obtained as white solid (yield 73%), MW = 446.46, mp 194–195 °C, ^1^H NMR (300 MHz, CDCl_3_) δ 8.40–8.34 (m, 1H, Ar-H1), 8.25 (d, *J* = 8.9 Hz, 1H, Cyn-H5), 7.95 (d, *J* = 16.1 Hz, 1H, =CH-), 7.75 (dd, *J* = 8.6, 2.3 Hz, 1H, Ar-H3), 7.45 (t, *J* = 8.4 Hz, 2H, Ar-H4, Ar-H8), 6.95 (dd, *J* = 8.9, 2.4 Hz, 1H, Ar-H7), 6.89 (d, *J* = 2.4 Hz, 1H, Ar-H5), 6.56–6.45 (m, 2H, Cyn-H3, -CH=), 6.44 (d, *J* = 2.4 Hz, 1H, Cyn-H6), 5.33 (s, 2H, -CH_2_-), 3.94 (s, 3H, -OCH_3_), 3.86 (s, 3H, -OCH_3_), 3.83 (s, 3H, -OCH_3_), ESI-MS (*m*/*z*): [M + H]+ calcd. for C_26_H_22_O_7_, 447.46, found, 447.20 98.69%.

(6-methoxy-9-oxo-9*H*-xanthen-2-yl)methyl (*E*)-3-(3,4-dimethoxyphenyl)acrylate (**10**) was obtained as white solid (yield 76%), MW = 446.46, mp 189–190 °C, ^1^H NMR (300 MHz, CDCl_3_) δ 8.41–8.34 (m, 1H, Ar-H1), 8.25 (d, *J* = 8.9 Hz, 1H, Ar-H8), 7.74 (dd, *J* = 8.6, 2.3 Hz, 1H, Ar-H3), 7.68 (d, *J* = 15.9 Hz, 1H, =CH-), 7.51–7.42 (m, 1H, Ar-H7), 7.15–7.02 (m, 2H, Ar-H4, Ar-H5), 7.00–6.92 (m, 1H, Cyn-H5), 6.92–6.81 (m, 2H, Cyn-H2, Cyn-H6), 6.37 (d, *J* = 15.9 Hz, 1H, -CH=), 5.34 (s, 2H, -CH_2_-), 3.96–3.85 (m, 9H, -OCH_3_, -OCH_3_, -OCH_3_)., ESI-MS (*m*/*z*): [M + H]+ calcd. for C_26_H_22_O_7_, 447.46, found, 447.14 96.99%.

(6-methoxy-9-oxo-9*H*-xanthen-2-yl)methyl (*E*)-3-(2,3,4-trimethoxyphenyl)acrylate (**11**) was obtained as white solid (yield 68%), MW = 476.48, mp 188–190 °C, ^1^H NMR (300 MHz, CDCl_3_) δ 8.39 (dd, *J* = 2.3, 0.6 Hz, 1H, Ar-H1), 8.26 (d, *J* = 8.9 Hz, 1H, Ar-H8), 7.74 (dd, *J* = 8.6, 2.3 Hz, 1H, Ar-H3), 7.65 (d, *J* = 15.9 Hz, 1H, =CH-), 7.48 (d, *J* = 8.6 Hz, 1H, Ar-H7), 7.29–7.23 (m, 1H, Ar-H4), 7.01–6.93 (m, 1H, Cyn-H2), 6.90 (d, *J* = 2.4 Hz, 1H, Ar-H5), 6.76 (s, 1H, Cyn-H6), 6.42 (d, *J* = 15.9 Hz, 1H, -CH=), 5.35 (s, 2H, -CH_2_-), 3.94 (s, 3H, -OCH_3_), 3.88 (d, *J* = 2.0 Hz, 9H, -OCH_3_, -OCH_3_, -OCH_3_), ESI-MS (*m*/*z*): [M + H]+ calcd. for C_27_H_24_O_8_, 477.48, found, 477.18 100%.

(6-methoxy-9-oxo-9*H*-xanthen-2-yl)methyl (*E*)-2,3-diphenylacrylate (**12**) was obtained as white solid (yield 72%), MW = 462.50, mp 171–172 °C, ^1^H NMR (300 MHz, CDCl_3_) δ ppm 8.30 (d, *J* = 2.34 Hz, 1H, Ar-H1), 8.24 (d, *J* = 8.79 Hz, 1H, Ar-H8), 7.89 (s, 1H, =CH-), 7.65 (dd, *J* = 8.50, 2.05 Hz, 1H, Ar-H3), 7.33–7.44 (m, 4H, Ar-H4, Cyn-H3, Cyn-H5, Ph-H4), 7.11–7.27 (m, 5H, Cyn-H4, Ph-H2, Ph-H3, Ph-H2, Ph-H6), 7.03–7.08 (m, 2H, Cyn-H2, Cyn-H6), 6.94 (dd, *J* = 8.79, 2.34 Hz, 1H, Ar-H7), 6.87 (d, *J* = 2.34 Hz, 1H, Ar-H5), 5.35 (s, 2H, -CH_2_-), 3.92 (s, 3H, -OCH_3_), ESI-MS (*m*/*z*): [M + H]+ calcd. for C_30_H_22_O_5_, 463.50, found, 463.22 98.64%.

(7-chloro-9-oxo-9*H*-xanthen-2-yl)methyl (*E*)-3-(4-methoxyphenyl)acrylate (**13**) was obtained as white solid (yield 68%), MW = 420.85, mp 160–161 °C, ^1^H NMR (300 MHz, CDCl_3_) δ 8.38–8.26 (m, 2H, Ar-H8, Ar-H1), 7.80 (dd, *J* = 8.7, 2.3 Hz, 1H, Ar-H3), 7.76–7.62 (m, 2H, Ar-H5, =CH-), 7.58–7.42 (m, 4H, Ar-H4, Ar-H6, Cyn-H2, Cyn-H6), 6.96–6.85 (m, 2H, Cyn-H3, Cyn-H5), 6.37 (d, *J* = 15.9 Hz, 1H, -CH=), 5.34 (s, 2H, -CH_2_-), 3.84 (s, 3H, -OCH_3_), ESI-MS (*m*/*z*): [M + H]+ calcd. for C_24_H_17_ClO_5_, 421.85, found, 421.08 98.32%.

3-((9-oxo-9*H*-xanthen-2-yl)oxy)propyl (*E*)-3-(4-methoxyphenyl)acrylate (**14**) was obtained as white solid (yield 63%), MW = 430.46, mp 139–140 °C, ^1^H NMR (300 MHz, CDCl_3_) δ 8.39–8.29 (m, 1H, Ar-H8), 7.77–7.68 (m, 2H, Ar-H1, Ar-H7), 7.65 (d, *J* = 16.0 Hz, 1H, =CH-), 7.53–7.41 (m, 4H, Ar-H3, Ar-H5, Cyn-H2, Cyn-H6), 7.41–7.29 (m, 2H, Ar-H4, Ar-H6), 6.94–6.83 (m, 2H, Cyn-H3, Cyn-H5), 6.39–6.26 (m, 1H, -CH=), 4.42 (t, *J* = 6.2 Hz, 2H, -CH_2_-O), 4.23 (t, *J* = 6.1 Hz, 2H, O-CH_2_-), 3.82 (s, 3H, -OCH_3_), 2.24 (quint, *J* = 6.2 Hz, 2H, -CH_2_-), ESI-MS (*m*/*z*): [M + H]+ calcd. for C_26_H_22_O_6_, 431.46, found, 431.25 100%.

9-oxo-9*H*-xanthen-2-yl cinnamate (**15**) was obtained as white solid (yield 70%), MW = 342.35, mp 162–164 °C, ^1^H NMR (300 MHz, CDCl_3_) δ ppm 8.35 (dd, *J* = 7.91, 1.47 Hz, 1H, Ar-H8), 8.09–8.13 (m, 1H, Ar-H1), 7.92 (d, *J* = 15.82 Hz, 1H, =CH-), 7.71–7.79 (m, 1H, Ar-H6), 7.62 (dd, *J* = 6.45, 2.93 Hz, 2H, Cyn-H2, Cyn-H6), 7.55–7.58 (m, 2H, Ar-H4, Ar-H5), 7.52 (d, *J* = 7.62 Hz, 1H, Ar-H3), 7.42–7.47 (m, 3H, Ar-H7, Cyn-H3, Cyn-H5), 7.37–7.42 (m, 1H, Cyn-H4), 6.67 (d, *J* = 15.82 Hz, 1H, -CH=), ESI-MS (*m*/*z*): [M + H]+ calcd. for C_22_H_14_O_4_, 343.35, found, 343.12 100%.

9-oxo-9*H*-xanthen-3-yl cinnamate (**16**) was obtained as white solid (yield 64%), MW = 342.35, mp 173–174 °C, ^1^H NMR (300 MHz, CDCl_3_) δ ppm 8.39 (d, *J* = 8.79 Hz, 1H, Ar-H1), 8.35 (dd, *J* = 7.91, 1.47 Hz, 1H, Ar-H8), 7.93 (d, *J* = 16.41 Hz, 1H, =CH-), 7.58–7.77 (m, 3H, Ar-H6, Cyn-H2, Cyn-H6), 7.36–7.53 (m, 6H, Ar-H4, Ar-H7, Ar-H5, Cyn-H3, Cyn-H4, Cyn-H5), 7.22 (dd, *J* = 8.79, 2.34 Hz, 1H, Ar-H2), 6.66 (d, *J* = 15.82 Hz, 1H, -CH=), ESI-MS (*m*/*z*): [M + H]+ calcd. for C_22_H_14_O_4_, 343.35, found, 343.12 100%.

9-oxo-9*H*-xanthen-4-yl cinnamate (**17**) was obtained as white solid (yield 67%), MW = 342.35, mp 192–193 °C, ^1^H NMR (300 MHz, CDCl_3_) δ 8.40–8.29 (m, 1H, Ar-H8), 8.29–8.21 (m, 1H, Ar-H1), 8.00 (d, *J* = 16.0 Hz, 1H, =CH-), 7.76–7.54 (m, 4H, Ar-H6, Ar-H3, Cyn-H3, Cyn-H5), 7.52–7.33 (m, 6H, Ar-H7, Ar-H5, Ar-H2, Cyn-H2, Cyn-H6, Cyn-H4), 6.79 (d, *J* = 16.0 Hz, 1H, -CH=), ESI-MS (*m*/*z*): [M + H]+ calcd. for C_22_H_14_O_4_, 343.35, found, 343.12 100%.

### 3.2. Evaluation of Lipophilicity Parameter R_M0_

Reversed-phase thin-layer chromatography (RP-TLC) was performed on aluminum sheets 20 × 20 cm^2^ coated with silica gel RP-18 F254 (Merck, Darmstadt, Germany). The mobile phases constituted mixtures of methanol and 100 mM potassium-phosphate buffer (pH = 7.4), with the methanol content between 95 and 65% (*v*/*v*). The tested compounds were dissolved separately in chloroform (1 mg/mL) and 5 µL of solutions were transferred on the sheets. Chromatographic chambers were saturated with mobile phase for 1 h at room temperature before developing the sheets. The spots were observed in UV light. For each concentration of methanol R_f_ values were calculated and mean values are reported from two independent experiments (Table S1 Supplementary material). R_M_ values were calculated from the experimental R_f_ using equation: R_M_ = log(1/R_f_ − 1) (Table S2 Supplementary material). The calculated values of R_M_ were extrapolated for 0% methanol concentration (Table 1) [33,34].

### 3.3. Ultraviolet Spectroscopy

Spectra with a scan range of 290–400 nm were recorded in 30 µM chloroform solutions, in 1 cm path length, 1.5 mL quartz cuvettes on a U-2800 double beam spectrophotometer (Hitachi, Tokyo, Japan) controlled by UV Solution version 2.2 software. The molar extinction coefficient at maximum absorption (εmax) of tested compounds was determined in chloroform as the slope of the linear regression of absorbance vs. concentration of tested compound (from 2.5 to 30 µM). E(1,1) coefficient was calculated from the formula:

$$E_{1,1}=\varepsilon \left[\frac{L}{mol\;cm}\right]\times \frac{10\left[\frac{g}{L}\right]}{M\left[\frac{g}{mol}\right]}\times 1\left[cm\right]$$

‹*E*_1,1_›_mean_ value is a mean value of the specific extinction over the spectral range from 290 to 400 nm.

### 3.4. Photoprotective Activity

#### 3.4.1. Preparation of Simple Cosmetic Formulations

To assess the activity of compounds **8** and **9** as UV filters, they were incorporated into a macrogol cream. This cream was made of PEG 400 and PEG 1500 mixed at 70 °C in 1:1 (*w*/*w*) ratio. Then, two concentrates, containing 50% (*w*/*w*) of the tested compounds **8** or **9** were prepared with the aim to combine them with this macrogol cream. First, the tested compounds were mixed with triacetin in 1:1 (*w*/*w*) ratio. Each mixture was wet milled using a high-energy planetary ball mill (Pulverisette 7 classic line, Fritsch, Germany). The total milling time was 12 h, but to avoid the overheating of the sample, the milling procedure was performed in cycles, i.e., the milling periods of 20 min were followed by the pause periods of 10 min. The rotation speed of the solar disc was kept at 400 rpm. After such processing, the concentrates in the form of a white paste were obtained. Then, each of these concentrates was mixed with the macrogol cream at 70 °C in three wt. ratios, namely 1:3, 1:5.25 or 1:9.

#### 3.4.2. In Vitro Photoprotection Study

In vitro photoprotective activity of compounds **8** and **9** was evaluated according to EN ISO 24443:2012 [38] with slight modifications. 32.5 mg of formulation with tested compound was spread onto roughened polymethylmethacrylate plate (PMMA) purchased from Schonberg GmbH (Hamburg, Germany) with the application area 25 cm^2^ and 5 µm roughness value to simulate the skin surface. After 30 min in the dark in vitro absorbance of samples was measured from 290 to 400 nm with 1 nm steps by reflectance spectrophotometry. SPF-290AS Analyser (Solar Light Company, Glenside, PA, USA) equipped with integrating sphere coupled with a WinSPF version 4.4 software was used. For each sample two plates were prepared, absorbance measures were performed on 6 different positions of the plate. The results were expressed as an average of data from 12 scans.

### 3.5. Photostability Study

To evaluate functional photostability of compounds **8** and **9** PMMA plates with tested compounds was irradiated with solar light simulator (Suntest CPS+, Atlas, Linsengericht, Germany) equipped with an optical filter cutting off wavelengths shorter than 290 nm and IR-block filter to neutralize thermal effects. The light source emission was maintained at 500 W/m^2^ and irradiation procedure was conducted for 1 h which corresponds to cumulative dose of ultraviolet radiation 218 kJ/m^2^ and is consistent with previous studies [10,54]. The UV absorption spectrum of the samples and photoprotective activity parameters were analysed post-irradiation and compared with pre-irradiation results. All analyses were performed in duplicates.

### 3.6. In Vitro Viability Assessment

Skin fibroblast BJ (ATCC CRL 2522), keratinocytes cell line HaCaT (Sigma Aldrich) human liver cancer HepG2 cell line (ATCC HB8065), rat cardiomyoblast H9c2 (ATCC CRL1446), and mouse astrocytes (ATCC, CRL2541) were cultured in standard conditions (37 °C, 5% CO_2_, 95% humidity) in appropriate culture medium (according to manufacturer procedure), supplemented with 10% fetal bovine serum (FBS, Gibco) and antibiotics (1% streptomycin/penicillin mixture; Sigma-Aldrich). Cells were seeded at density of 2 × 10^4^ (BJ, HaCaT) on 96-well plates. After 24 h cells were treated with increasing doses of compounds and incubated for additional 24 h. Following the incubation, MTT reagent (Sigma-Aldrich, Darmstadt, Germany) was added to each well and after 4 h incubation, the medium was aspirated, and the formazan produced in the cells appeared as dark crystals in the bottom of the wells. Next, DMSO (dissolving solution) was added to each well. Then, the absorbance of solution was determined at 570 nm (A570) on plate reader (Spectra iD3 Max, Molecular Devices; San Jose, CA, USA). Viability (% of control) was determined by dividing A570 of experimental wells by of A570 of control wells × 100%. Data from the cytotoxicity tests were subjected to one-way analysis of variance, followed by Dunnett’s test using GraphPad Prism 7.0 Software (GraphPad Software Inc., San Diego, CA, USA). Values of *p* < 0.05 were considered to be statistically significant.

### 3.7. Estrogenic Activity

Estrogenic activity of tested compounds was investigated by study of their influence on estrogen-dependent MCF-7 breast cancer cell line proliferation. Cells were seeded into 24-wells plates in density of 40,000 per well. After 24 h, cells were washed twice with PBS and fresh, estrogen- and phenol red-free medium (DMEM low-glucose; 2.5% Charcoal Stripped FBS; 2 mM glutamine; Sigma Aldrich, Darmstadt, Germany) was added. Then cells were incubated with tested compounds and benzophenone-2 (BP-2; Sigma Aldrich, Darmstadt, Germany) to final concentration of 5 μM. After 120 h incubations, cells proliferation was determined with crystal violet assay. Briefly, cells were washed with PBS and fixed with 3.7% formaldehyde. Then, crystal violet solution was added for 10 min. After incubation, solution was removed, and cells were washed five times with PBS. Crystal violet was extracted from cells using destaining solution (1.33% citric acid, 1.09% sodium citrate in water/methanol (1:1) solution) and absorbance of the solution was determined at 570 nm (A570). Proliferation rate was determined by dividing the A570 of experimental wells by the A570 of control wells × 100%.

### 3.8. Mutagenicity Assay Procedure

Test compounds mutagenic effects were evaluated using the Ames microplate format (MPF) mutagenicity assay with Salmonella typhimurium TA98, TA100, TA1535 and TA1537 strains (Xenometrix, Allschwil, Switzerland). These strains meet the requirements of the Organization for Economic Cooperation and Development (OECD) guideline 471 for testing of chemicals [55]. Culture media, positive control chemicals, and tester strains were provided in the kit. TA100 and TA1535 strains are used to detect mutagens that cause base-pair substitutions, while TA98 and TA1537 detect frameshift mutations.

MPF assay is a modification of the traditional Salmonella test [56,57]. During the assay, bacteria are exposed to a test compound in a medium containing histidine to support cell divisions. Then, the cultures are diluted using indicator medium which lacks the required amino acid and selects for prototrophic reversion, and aliquoted into 48 wells of a 384-well plate. Within two days, bacteria that have undergone reversion to amino acid prototrophy will form colonies. The indicator medium turns yellow as the pH drops as a result of catabolic activity of revertant bacteria that grow in the absence of the required amino acid [58].

In the present study bacterial cultures were grown overnight (16 h) in growth medium and then exposed to test compounds (**8** and **9**) in 24-well plates for 90 min at 37 °C with constant shaking. Compounds were tested at final concentrations of 0.1, 0.2, and 0.5 mM. After preincubation, the cultures were diluted in the indicator medium and the contents of each 24-well culture were transferred to 48 wells on a 384-well plate. Upon completion of 48 h incubation period, yellow revertant wells were counted for each dose and compared with the number of spontaneous revertants obtained with the negative control. The standard positive controls for the Ames MPF assay were 2-nitrofluorene (2-NF) at 2 µg/mL (TA98); 4-nitroquinoline-N-oxide (4-NQO) at 0.1 µg/mL (TA100); N4-aminocytidine (N4-ACT) at 100 µg/mL (TA1535) and 9-aminoacridine (9-AAc) at 15 µg/mL (TA1537), whereas pure DMSO was used as a negative control. Each dose was done in triplicate [58,59,60,61].

Two criteria were applied to evaluate mutagenicity assay results such as the fold increase in the number of positive wells over the solvent control baseline (FIB), and the dose dependent response. FIB was calculated by dividing the mean number of positive wells at each dose by the solvent control at baseline (mean of negative control plus 1 SD). When an induction of more than twofold relative to the baseline at more than one dose with a dose-response was observed the sample was considered positive, whereas when no response >2 times the baseline and no dose-response was stated the sample was classified as negative in the assay [57,58,59].

## 4. Conclusions

We designed and synthetized 17 novel structures containing two chromophores: xanthone and (*E*)-cinnamoyl. Among the presented group of compounds, there were potential UVA and UVB filters, their λ_max_ was in the range from 290 to 329 nm, additionally compounds were characterized by high extinction coefficient and favorable lipophilicity predisposing to cumulate in stratum corneum. The most promising compound in the tested series was compound **9** ((6-methoxy-9-oxo-9*H*-xanthen-2-yl)methyl (*E*)-3-(2,4-dimethoxyphenyl)acrylate). It showed two peaks of λ_max_, one in UVB and one in UVA II region. After milling, it was loaded into a simple cream base at the concentration of 12.5% and showed very beneficial photoprotective properties (SPF_in vitro_ 19.69 ± 0.46, UVA PF 12.64 ± 0.32), which were comparable to tris-biphenyltiazine. Moreover, high value of critical wavelength (381 nm) indicated that compound **9** was a good candidate for a broad-spectrum UV filter that may protect skin against UVA-induced photoaging. Compound **9** in high concentration (50 µM) was deprived of cytotoxic activity toward human keratinocytes, human skin fibroblasts, mouse astrocytes and hepatoma cells. Compound **9** showed no estrogenic activity in MCF-7 breast cancer model. It also showed no mutagenic activity, the performed Ames test indicated that compound **9** was not base substitution or frameshift mutagen. According to the results of performed research it can be concluded, that in the group of xanthone derivatives new UV filters may be identified.

## Figures and Tables

**Table 1:**
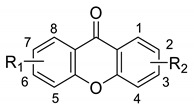
Chemical structures and lipophilicity parameters of tested compounds.

Compound	R_1_	Position of R_2_	R_2_	miLogP *	R_M0_ **
**1**	H	4-	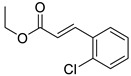	6.16	5.02
**2**	H	4-	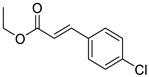	6.38	5.28
**3**	6-OCH_3_	4-	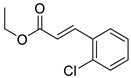	6.19	5.66
**4**	6-OCH_3_	4-	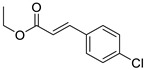	6.42	5.06
**5**	6-OCH_3_	4-	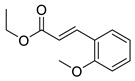	5.57	5.10
**6**	6-OCH_3_	4-	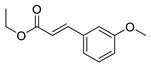	5.77	4.96
**7**	6-OCH_3_	4-	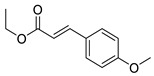	5.79	5.06
**8**	6-OCH_3_	2-	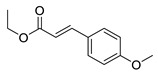	5.82	6.15
**9**	6-OCH_3_	2-	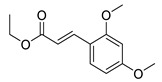	5.62	4.64
**10**	6-OCH_3_	2-	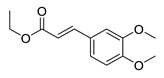	5.41	4.53
**11**	6-OCH_3_	2-	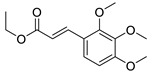	5.39	4.50
**12**	6-OCH_3_	2-	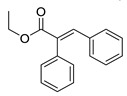	7.10	5.62
**13**	7-Cl	2-	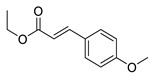	6.44	4.85
**14**	H	2-	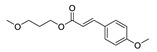	6.14	5.18
**15**	H	2-	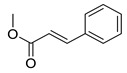	5.24	4.19
**16**	H	3-	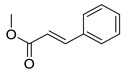	5.24	4.54
**17**	H	4-	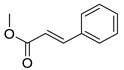	5.21	4.25

* calculated LogP values by Molispiration on-line tool, ** relative lipophilicity parameter determined by reversed-phase thin-layer chromatography.

**Table 2:** Ultraviolet spectroscopic properties of the tested xanthone derivatives and reference UV filters obtained in chloroform solutions.

Compound	Absorption Range (nm)	λ_max_ (nm)	ε_max_ (M^−1^ cm^−1^)	E(1,1) _(λmax)_ (g^−1^ L cm^−1^)	‹*E*_1,1_›_mean_
**1**	290–352	290	15,311	392	69
**2**	290–351	290	25,688	657	133
**3**	290–342	290	27,690	658	168
**4**	290–341	296	36,895	948	202
**5**	290–350	290	24,118	580	221
**6**	290–342	290	25,749	619	168
**7**	290–346	300	39,002	937	342
**8**	290–348	303	38,128	916	314
**9**	290–369	299, 329	34,534, 34,177	774, 766	360
**10**	290–355	301, 326	31,005, 30,469	694, 682	307
**11**	290–354	304	33,330	898	326
**12**	290–340	299	21,409	463	112
**13**	290–359	312	26,590	633	243
**14**	290–375	300	28,360	659	214
**15**	290–355	290	38,152	1115	188
**16**	290–345	290	36,410	1064	235
**17**	290–351	290	20,100	587	120
**4-MBC**	290–328	300	22,053	867	221
**OCT**	290–347	306	14,330	396	141
**EHMC**	290–337	309	24,907	858	266
**BMDM**	290–391	357	35,939	1158	520

**Table 3:** Photoprotective activity of compounds **8** and **9** obtained in 12.5% (*w*/*w*) cosmetic formulation.

Compound	SPF_in vitro_ ± SD	UVA PF ± SD	λ*_c_* (nm)	UVA/UVB Ratio
**8**	14.69 ± 2.25	4.92 ± 0.78	345	0.329
**9**	19.69 ± 0.46	12.64 ± 0.32	381	0.830

Results are presented as mean ± SD from two independent experiments, at each determination six scans were performed.

**Table 4:** The changes in photoprotective activity of compounds **8** and **9** after irradiation with solar light simulator at 500 W/m^2^.

Compound	% of Initial SPF_in vitro_	% of Initial UVA PF	λ*_c_*(nm)		UVA/UVB Ratio	
			Pre-Irradiation	Post-Irradiation	Pre-Irradiation	Post-Irradiation
**8**	76.05	67.20	345	335	0.339	0.201
**9**	89.03	82.75	381	380	0.830	0.784

Results are presented as means from two independent experiments, at each determination six scans were performed.

**Table 5:** Mutagenic evaluation of test compounds in the Ames microplate format (MPF) assay.

Positive Wells per Microplate					
		*Salmonella typhimurium*			
		TA98	TA100	TA1535	TA1537
Compound	Concentration (mM)	FIB *			
**8**	0.1	0.5	0.1	0.7	0.7
	0.2	1.1	1.2	0.4	1.0
	0.5	1.0	1.3	0.3	0.7
**9**	0.1	0.4	0.8	0.1	1.0
	0.2	0.2	0.7	0.8	0.3
	0.5	0.5	0.8	0.7	1.0
Positive control **		6.4	3.4	14.7	48.0

* FIB—fold induction over baseline (baseline = mean zero-dose control + 1 SD); SD—standard deviation. ** Positive controls: 2-nitrofluorene (2-NF) at 2 µg/mL (TA98); 4-nitroquinoline-N-oxide (4-NQO) at 0.1 µg/mL (TA100); N^4^-aminocytidine (N^4^-ACT) at 100 µg/mL (TA1535); 9-aminoacridine (9-AAc) at 15 µg/mL (TA1537).

## Data Availability

Data is contained within the article and supplementary material.

## Supplementary Materials

The following are available online at https://www.mdpi.com/1422-0067/22/1/34/s1.
