# Supplementary material for: The Involvement of Xanthone and (E)-Cinnamoyl Chromophores for the Design and Synthesis of Novel Sunscreening Agents
Source: Int J Mol Sci. 2020 Dec 22;22(1):34. doi: 10.3390/ijms22010034 (PMC7792956; doi:10.3390/ijms22010034)
Supplement: Supplementary file 1 [file ijms-22-00034-s001.pdf]

The involvement of xanthone and *(E)*-cinnamoyl chromophores for the design and synthesis of novel sunscreening agents

Table S1. Values of obtained R_f in RP-TLC experiment using as mobile phase mixtures of methanol and 100 mM potassium-phosphate buffer in different concentrations.

Compound	R_f obtained in methanol/potassium phosphate buffer (v/v)				
	95:5	90:10	80:20	75:25	65:35
**1**	0.294	0.191	0.074	0.066	0.012
**2**	0.277	0.209	0.067	0.036	0.000
**3**	0.254	0.176	0.063	0.042	0.007
**4**	0.325	0.152	0.071	0.042	0.000
**5**	0.282	0.189	0.073	0.051	0.011
**6**	0.283	0.186	0.072	0.048	0.013
**7**	0.295	0.172	0.075	0.054	0.011
**8**	0.259	0.182	0.041	0.018	0.007
**9**	0.286	0.157	0.067	0.048	0.020
**10**	0.345	0.231	0.103	0.059	0.023
**11**	0.345	0.254	0.114	0.079	0.022
**12**	0.222	0.170	0.057	0.038	0.006
**13**	0.312	0.210	0.054	0.045	0.018
**14**	0.287	0.158	0.083	0.048	0.010
**15**	0.272	0.255	0.119	0.088	0.022
**16**	0.260	0.218	0.096	0.064	0.017
**17**	0.329	0.291	0.140	0.095	0.025

Table S2. R_M values calculated from the R_f obtained in RP-TLC performed for tested compounds according to the equation: $R_M=\log(1/R_f-1)$ and correlation coefficients (R^2).

Compound	R_M for different mobile phases					R^2
	95:5*	90:10*	80:20*	75:25*	65:35*	
**1**	0.380	0.627	1.100	1.153	1.934	0.967
**2**	0.416	0.578	1.143	1.425	-	0.993
**3**	0.467	0.671	1.169	1.358	2.178	0.975
**4**	0.316	0.743	1.118	1.359	-	0.972
**5**	0.406	0.632	1.104	1.271	1.937	0.989
**6**	0.404	0.640	1.113	1.297	1.881	0.996
**7**	0.379	0.684	1.094	1.243	1.937	0.982
**8**	0.457	0.653	1.368	1.732	2.175	0.990
**9**	0.397	0.730	1.141	1.293	1.684	0.982
**10**	0.279	0.522	0.938	1.199	1.602	0.999
**11**	0.279	0.469	0.893	1.066	1.647	0.991
**12**	0.346	0.690	1.220	1.407	2.201	0.975
**13**	0.343	0.575	1.245	1.325	1.725	0.975
**14**	0.396	0.727	1.043	1.296	2.001	0.974
**15**	0.427	0.467	0.872	1.018	1.650	0.956
**16**	0.453	0.554	0.975	1.163	1.774	0.977
**17**	0.309	0.387	0.788	0.979	1.586	0.971

*mobile phases: methanol/potassium phosphate buffer (v/v)

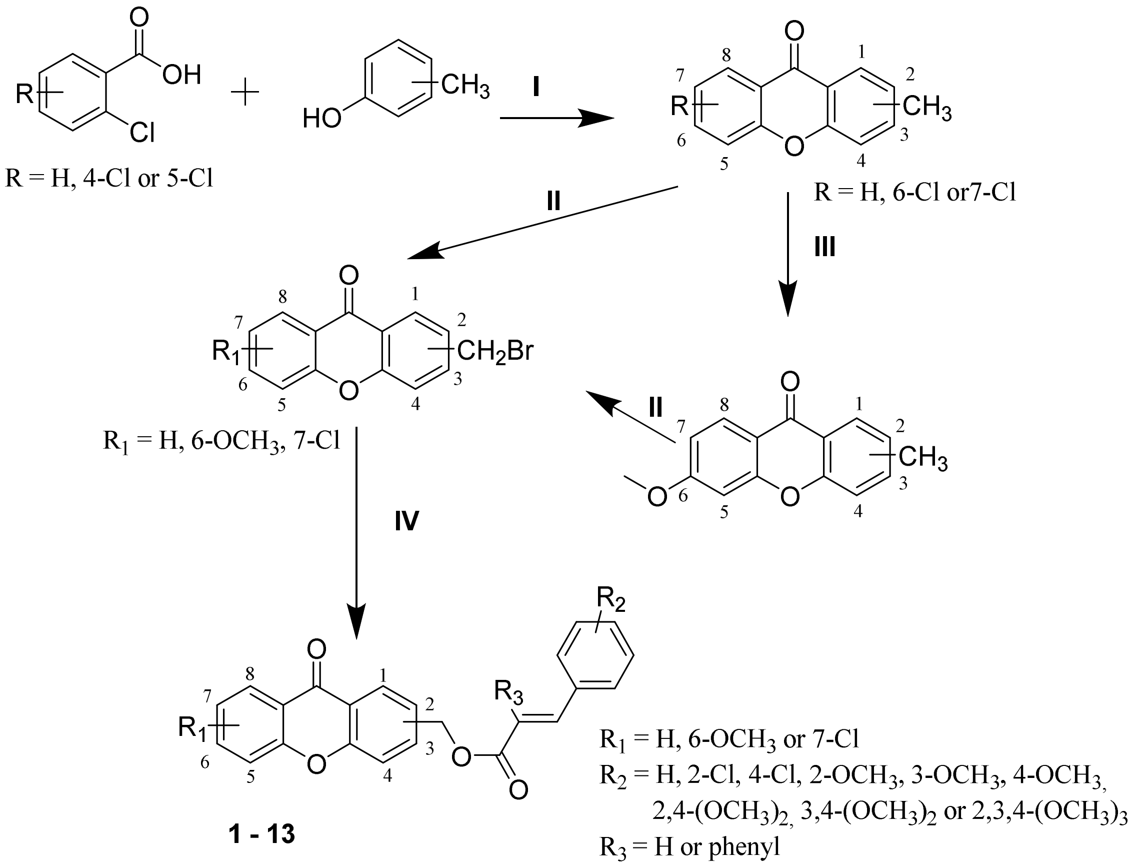

I - Ullmann`s condensation (C_2H_5ONa, paraffin oil (200-210^oC), and cyclization (conc. H_2SO_4 (boiling water bath, 2-4h))

II - Wohl-Ziegler bromination (NBS, benzoyl peroxide, *hv*)

III - methanolysis (CH_3ONa)

IV - 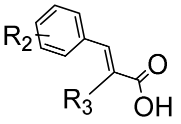

, DMF, K_2CO_3

Scheme S1. Synthetic pathway of compounds **1–13**.

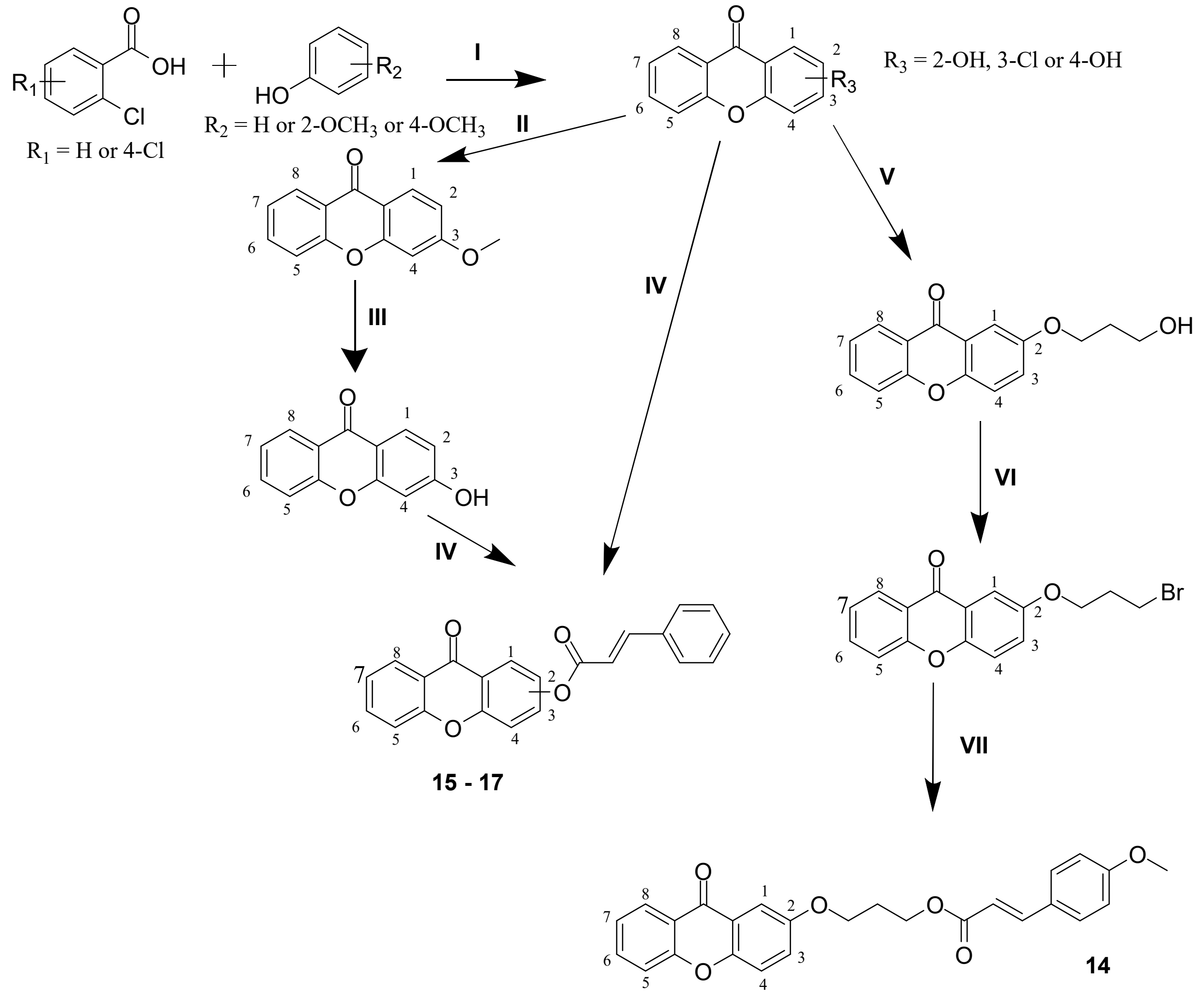

I - Ullmann`s condensation and cyclization (C_2H_5ONa, paraffin oil (200-210°C), and cyclization (conc. H_2SO_4 (boiling water bath, 2-4h))

II - methanolysis (CH_3ONa)

III - demethylation (80% H_2SO_4)

IV - 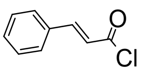 , DMF, K_2CO_3

V - 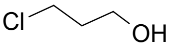 , K_2CO_3, acetone/ethanol, 50h

VI - PBr_3

VII - 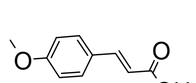 , DMF, K_2CO_3

Scheme S2. Synthetic pathway of compounds **14 – 17**.

1. NMR spectra of new compounds:

compound 1

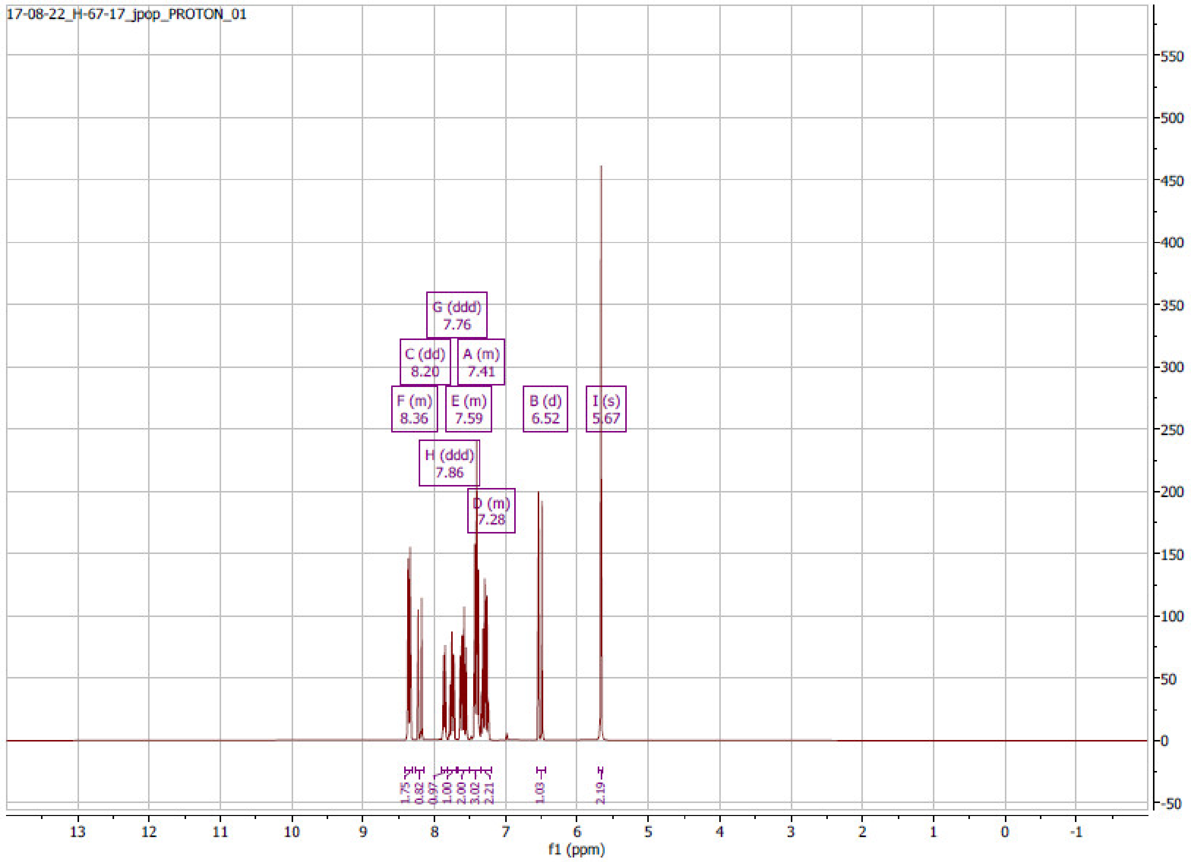

compound 2

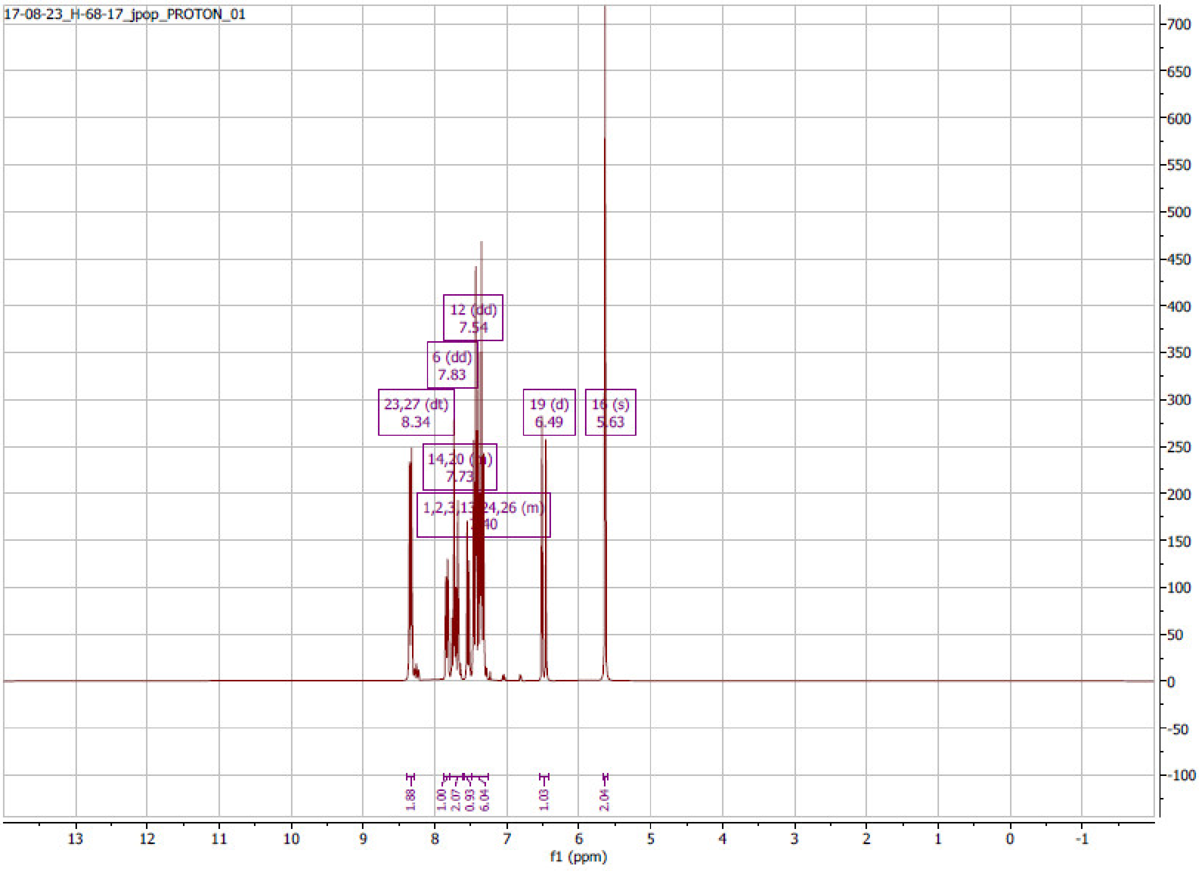
17-08-23_H-68-17_jpop_PROTON_01
12 (dd)
7.54
6 (dd)
7.83
23,27 (dt)
8.34
19 (d)
6.49
16 (s)
5.63
14,20 (m)
7.73
1,2,3,13,24,26 (m)
7.40
1.88
1.00
2.07
0.93
6.04
1.03
2.04
f1 (ppm)

compound 3

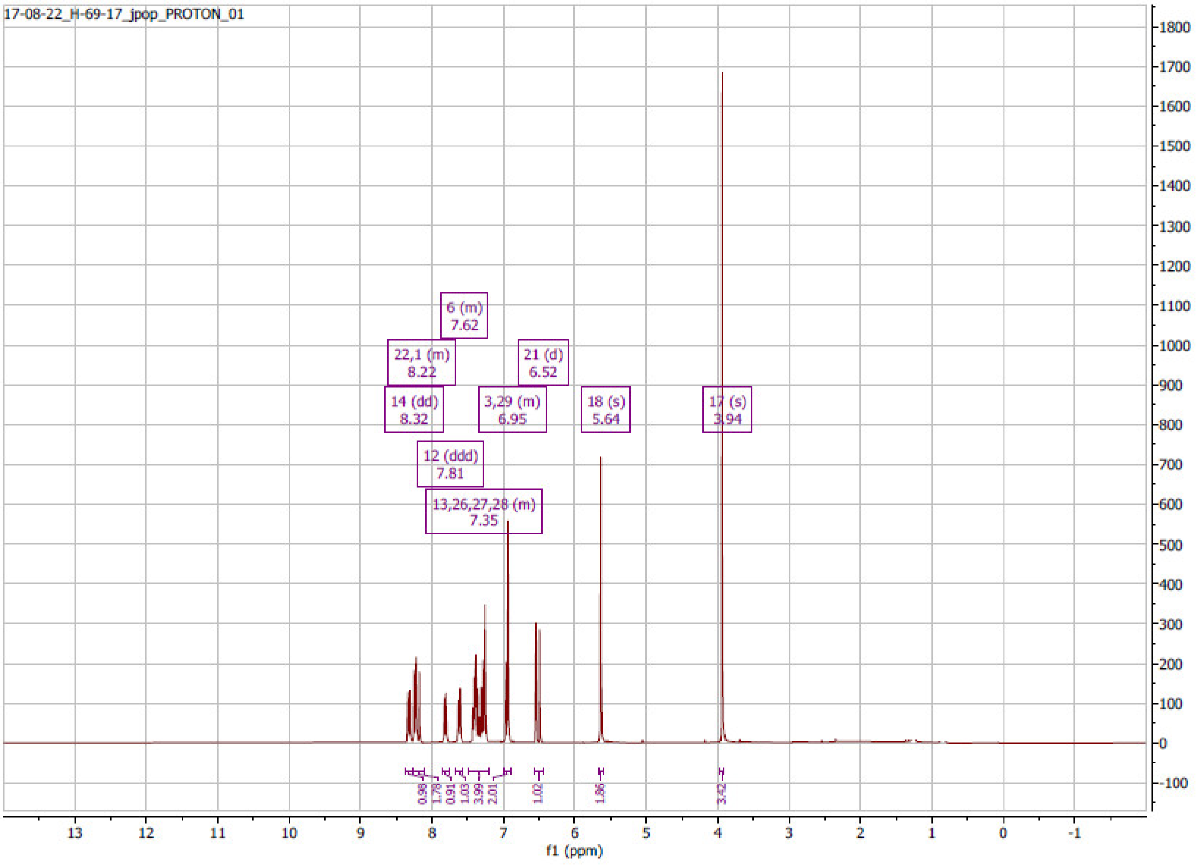
17-08-22_H-69-17_jpop_PROTON_01
6 (m)
7.62
22,1 (m)
8.22
21 (d)
6.52
14 (dd)
8.32
3,29 (m)
6.95
18 (s)
5.64
17 (s)
3.94
12 (ddd)
7.81
13,26,27,28 (m)
7.35
0.98
1.78
0.91
1.03
3.99
2.01
1.02
1.86
3.42
f1 (ppm)

compound 4

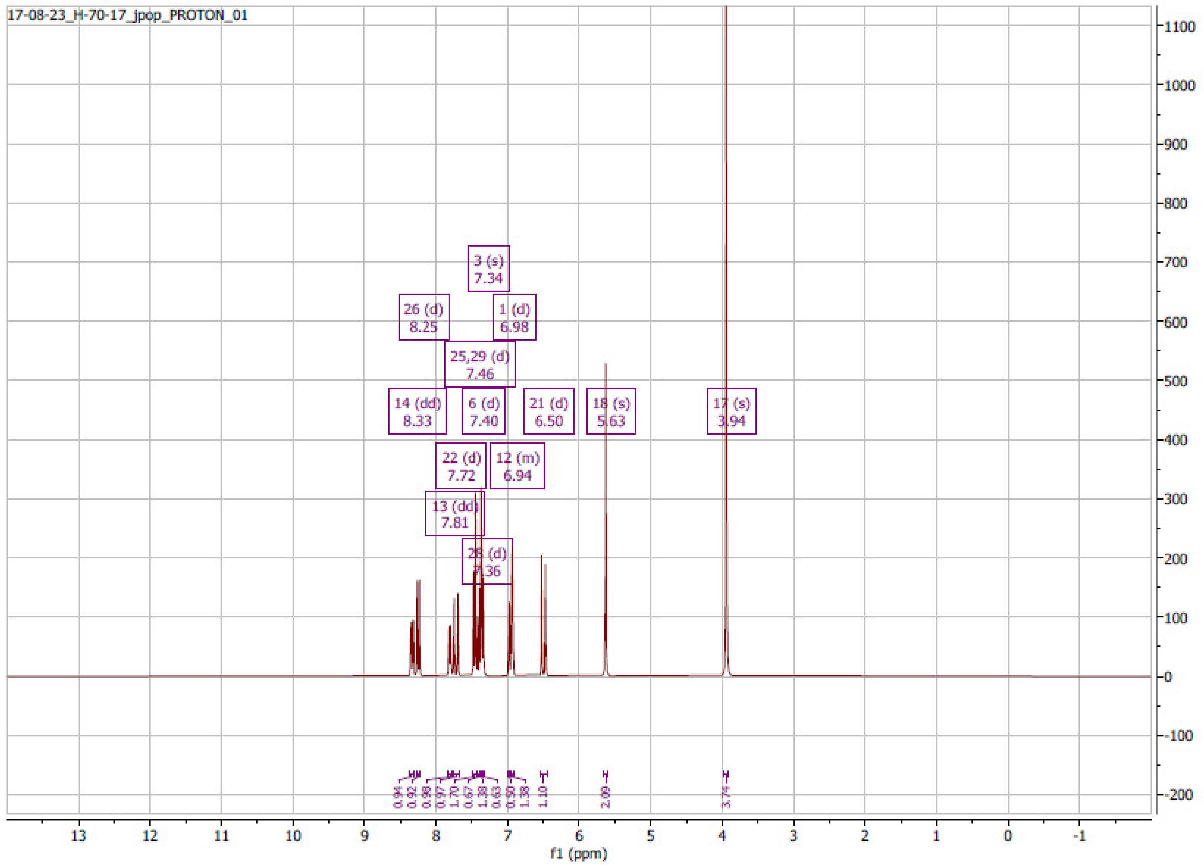

compound 5

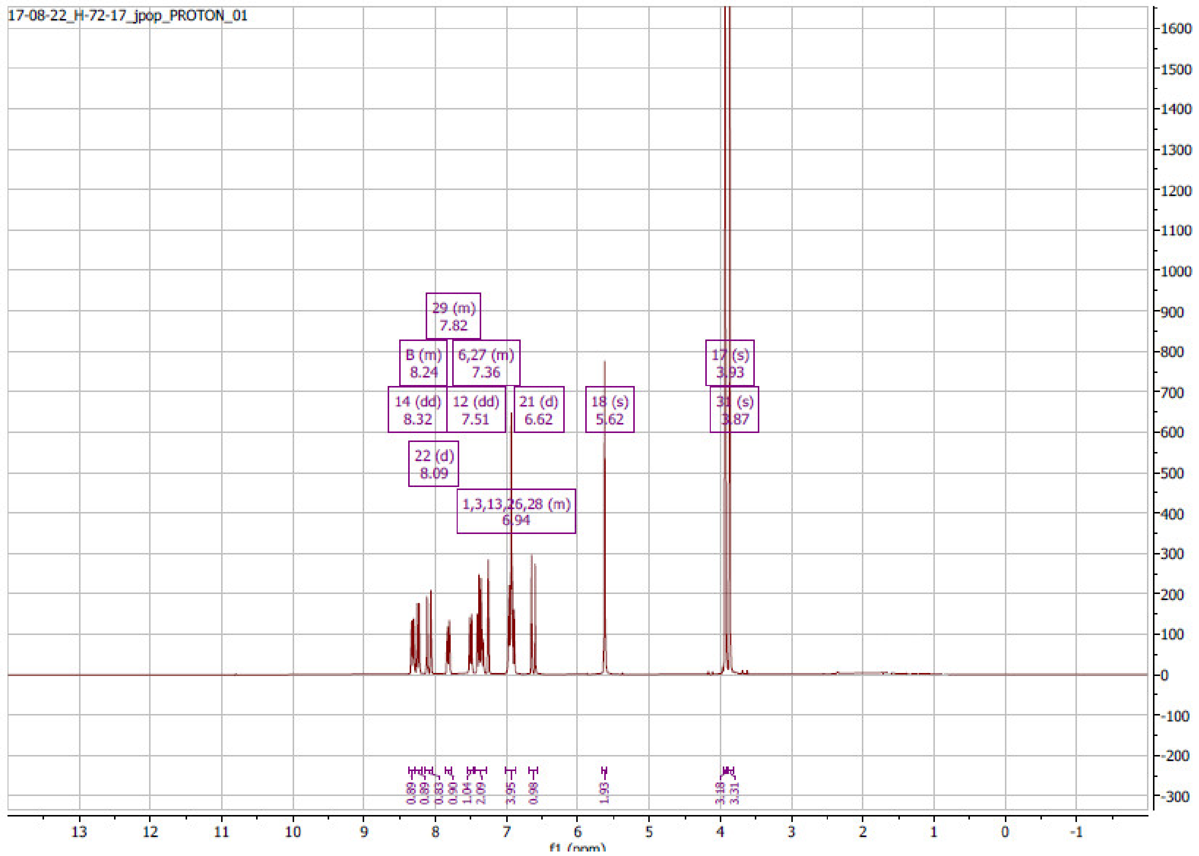
17-08-22_H-72-17_jpop_PROTON_01
29 (m) 7.82
B (m) 8.24
6,27 (m) 7.36
14 (dd) 8.32
12 (dd) 7.51
21 (d) 6.62
18 (s) 5.62
17 (s) 3.93
30 (s) 3.87
22 (d) 8.09
1,3,13,26,28 (m) 6.94
f1 (ppm)

compound 6

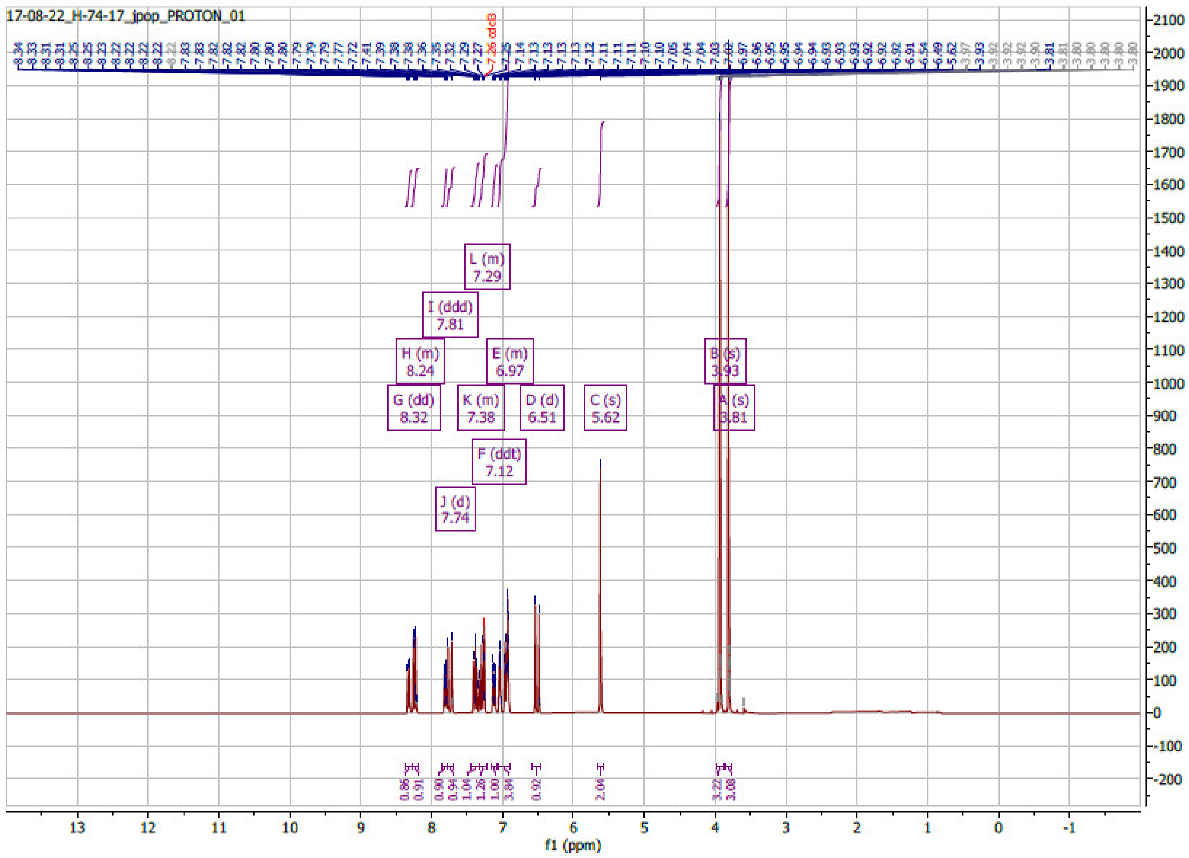
17-08-22_H-74-17_jpop_PROTON_01
L (m) 7.29
I (ddd) 7.81
H (m) 8.24
E (m) 6.97
B (s) 3.93
G (dd) 8.32
K (m) 7.38
D (d) 6.51
C (s) 5.62
A (s) 3.81
F (ddt) 7.12
J (d) 7.74
f1 (ppm)

compound 7

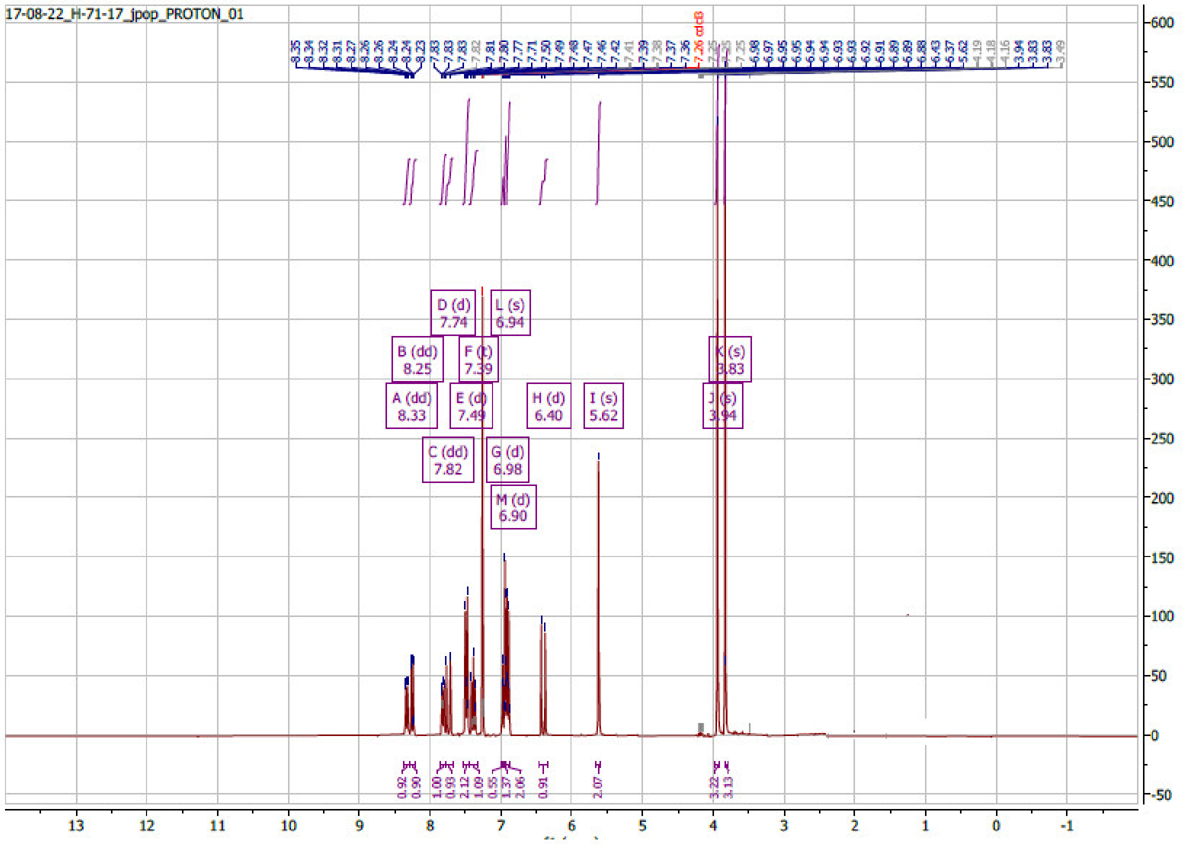

compound 8

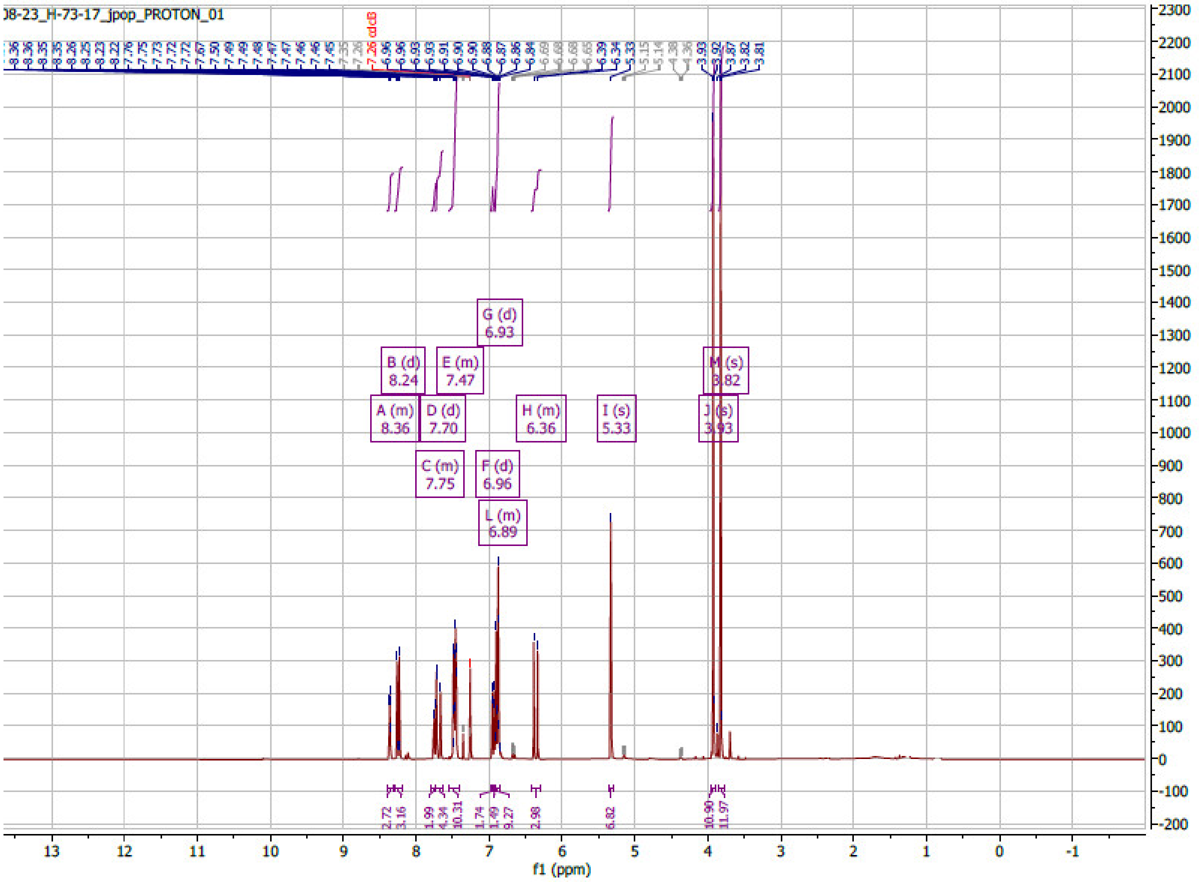
08-23_H-73-17_jpop_PROTON_01
cdcl3
A (m) 8.36
B (d) 8.24
C (m) 7.75
D (d) 7.70
E (m) 7.47
F (d) 6.96
G (d) 6.93
H (m) 6.36
I (s) 5.33
J (s) 3.93
L (m) 6.89
M (s) 3.82
f1 (ppm)

compound 9

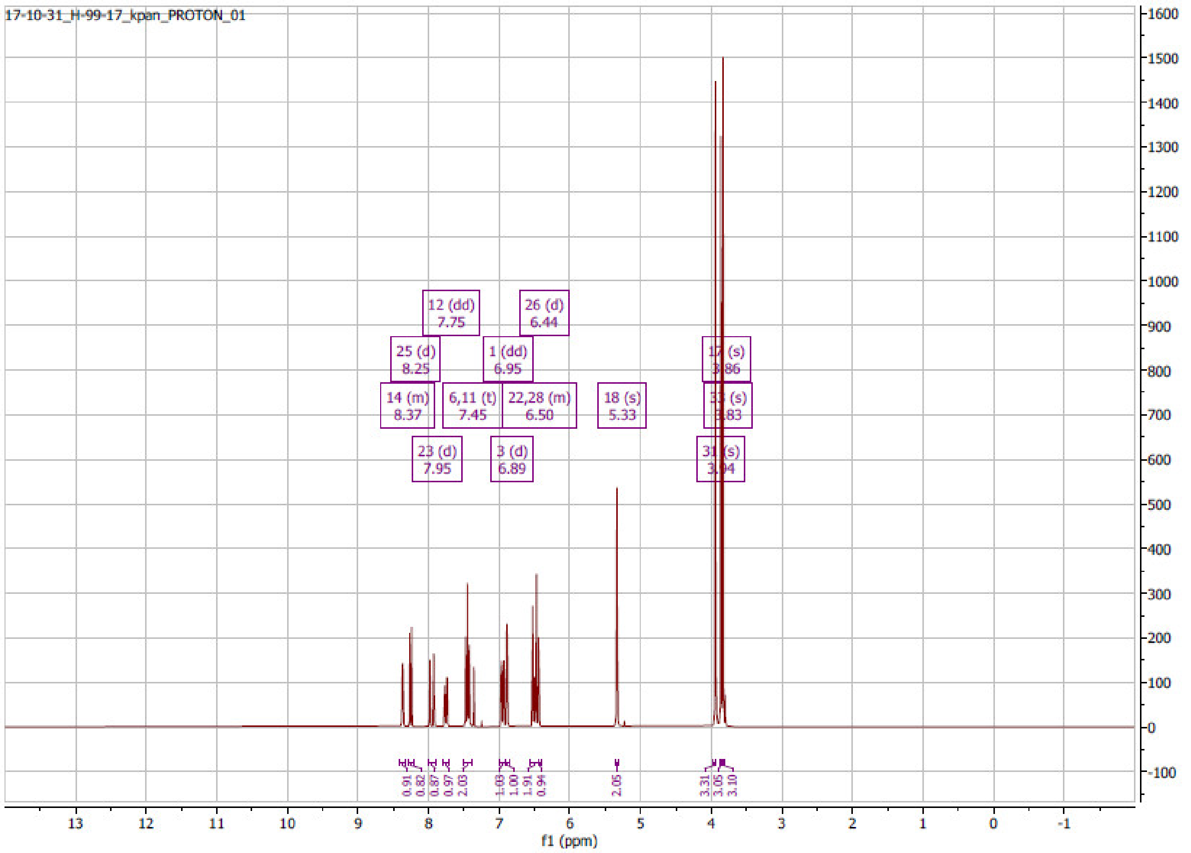
17-10-31_H-99-17_kpan_PROTON_01
f1 (ppm)

compound 10

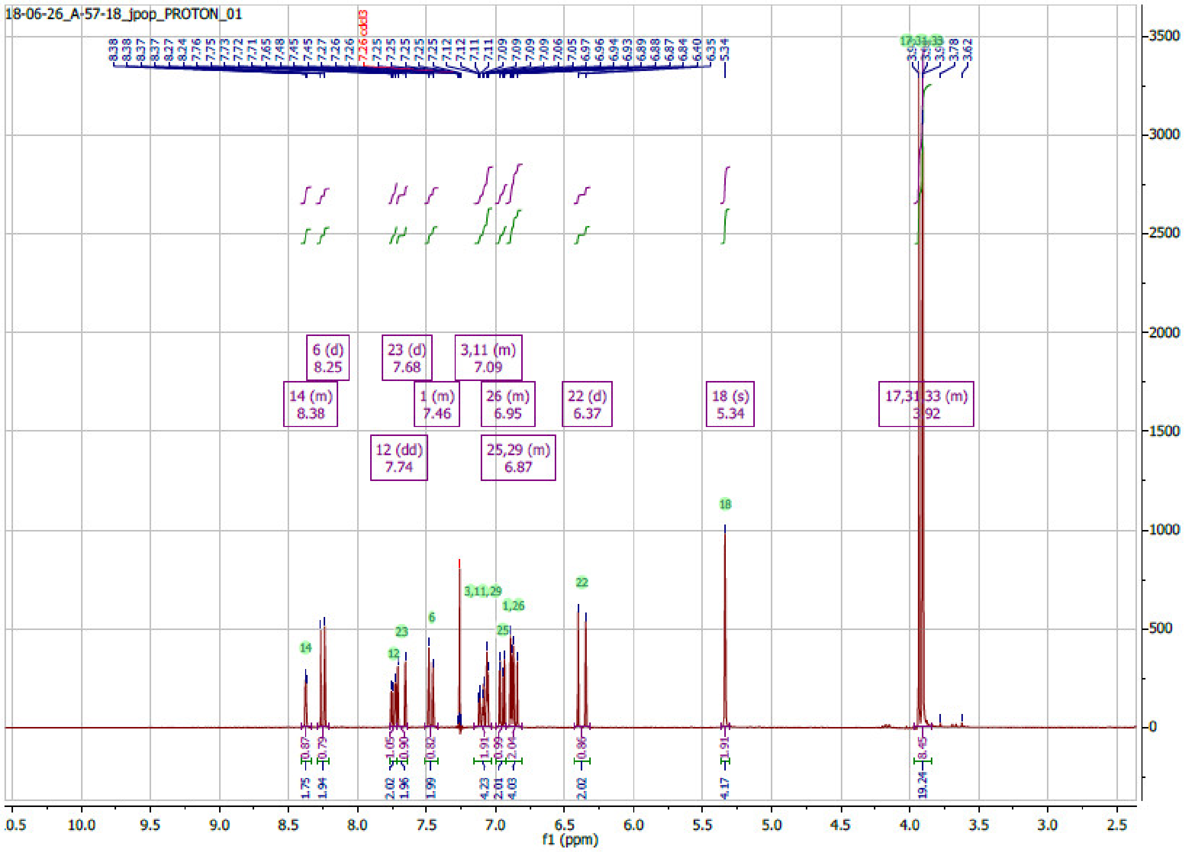
18-06-26_A-57-18_jpop_PROTON_01
f1 (ppm)

compound 11

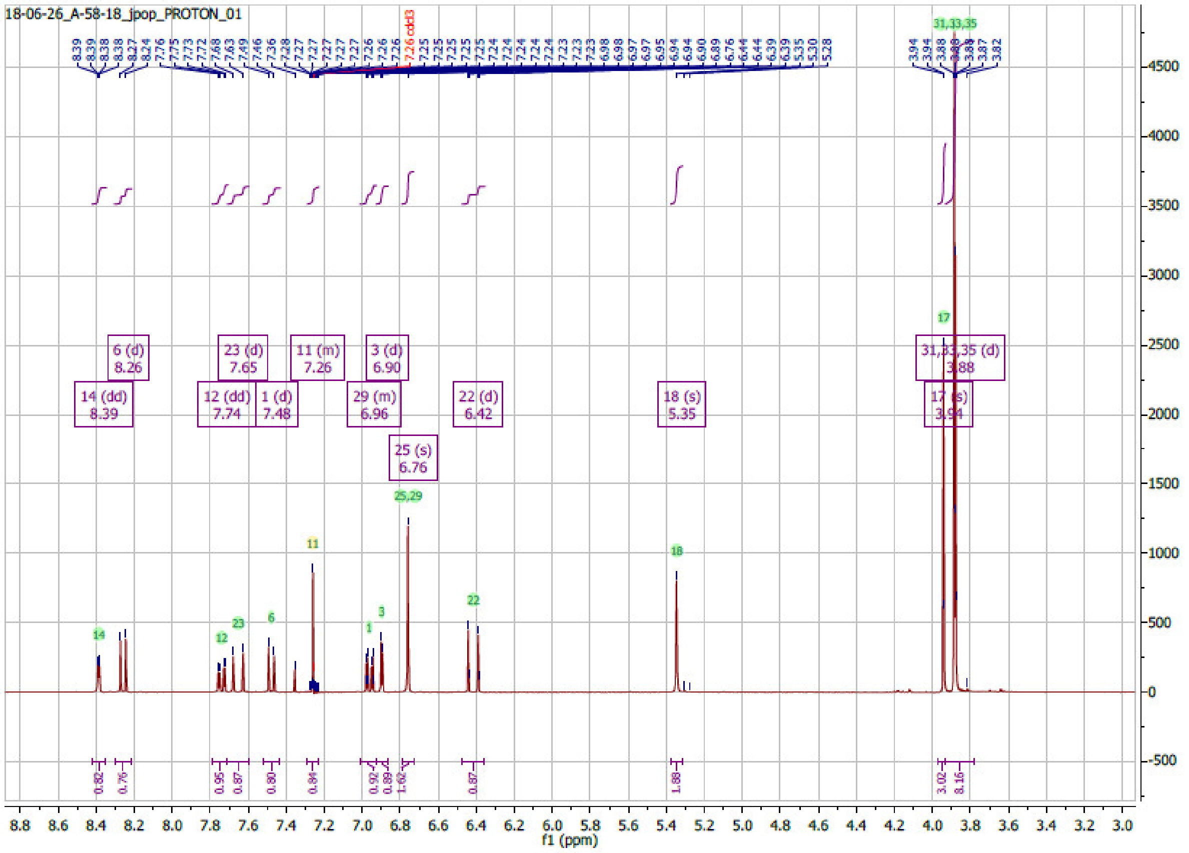

compound 12

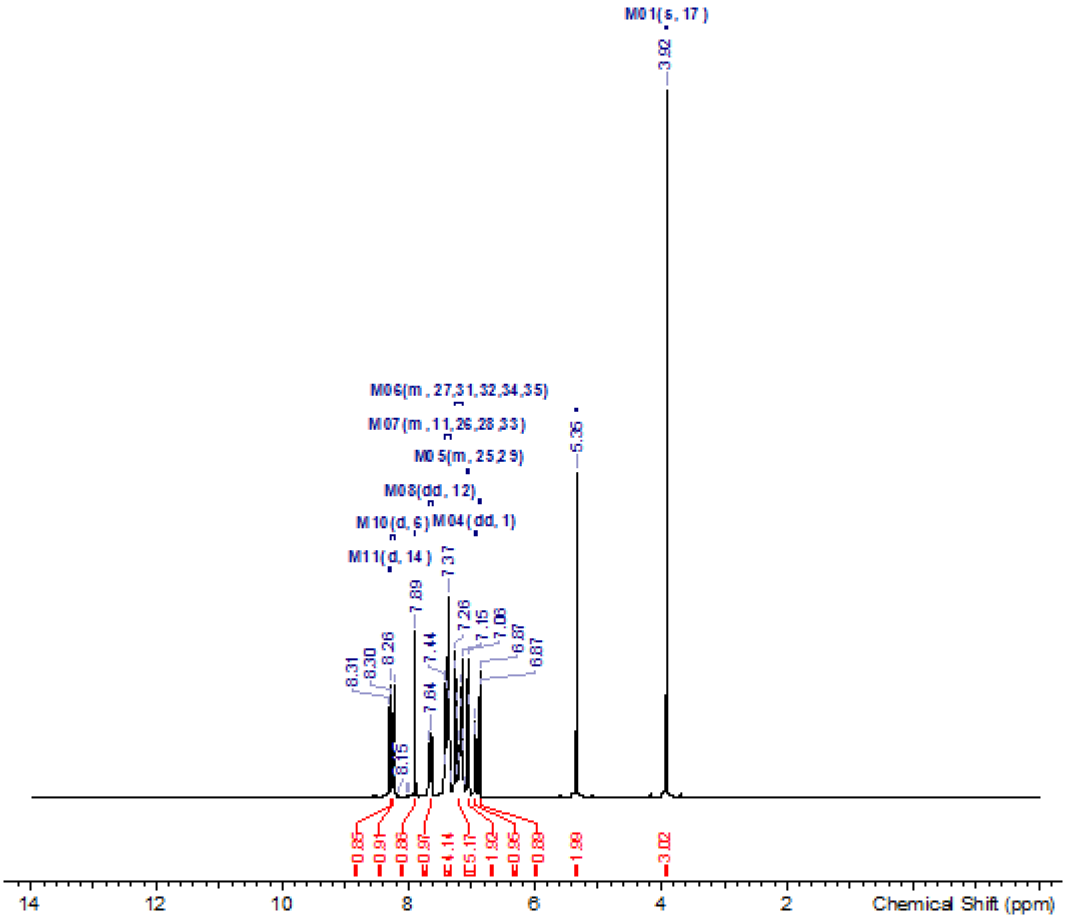

M01(s, 17)
M06(m, 27,31,32,34,35)
M07(m, 11,26,28,33)
M05(m, 25,29)
M08(dd, 12)
M10(d, 6)
M04(dd, 1)
M11(d, 14)
Chemical Shift (ppm)

compound 13

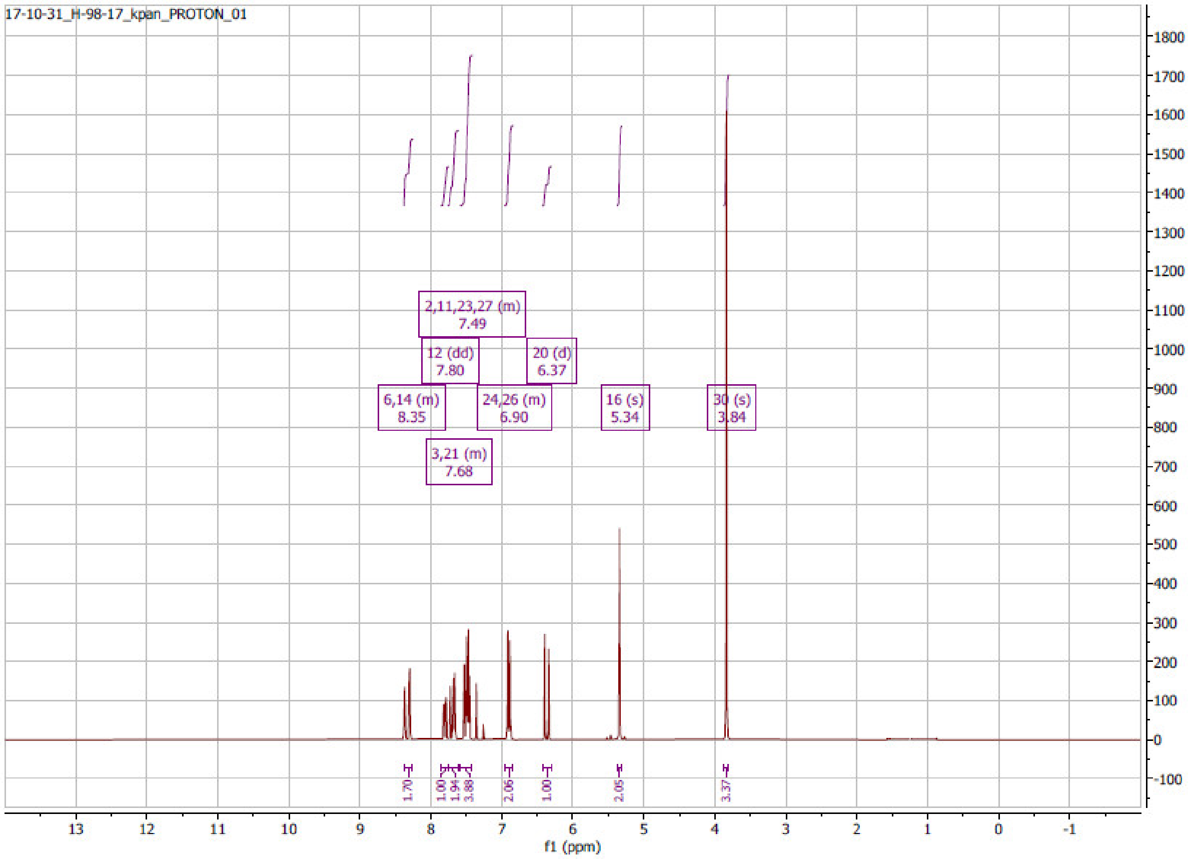

compound 14

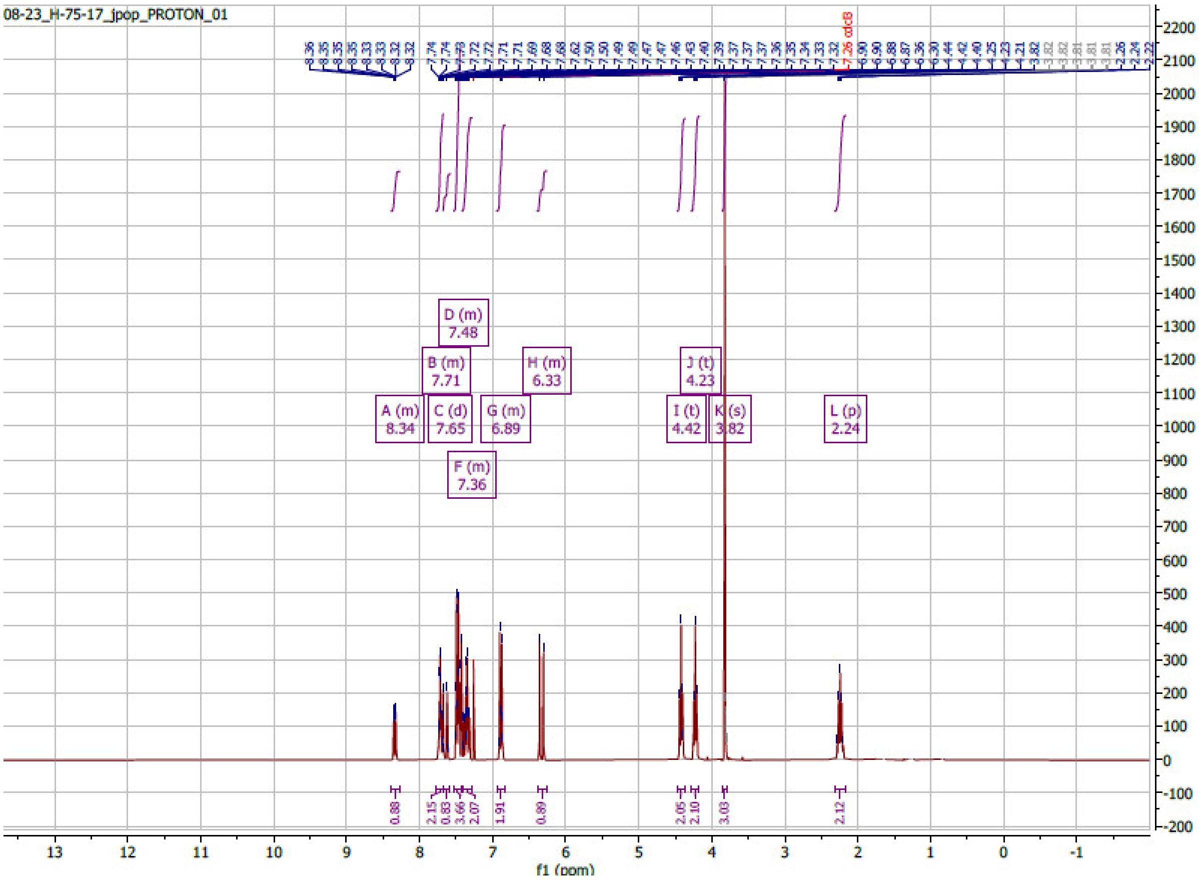

compound 15

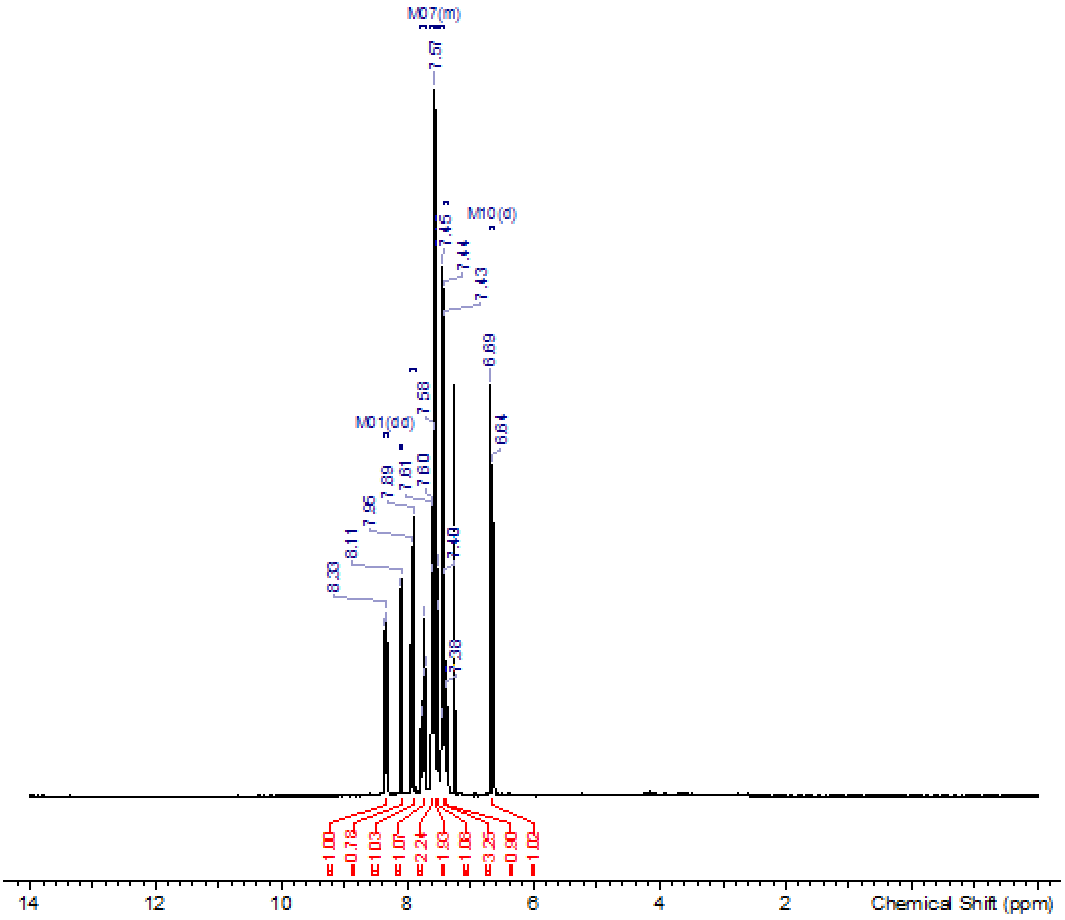

compound 16

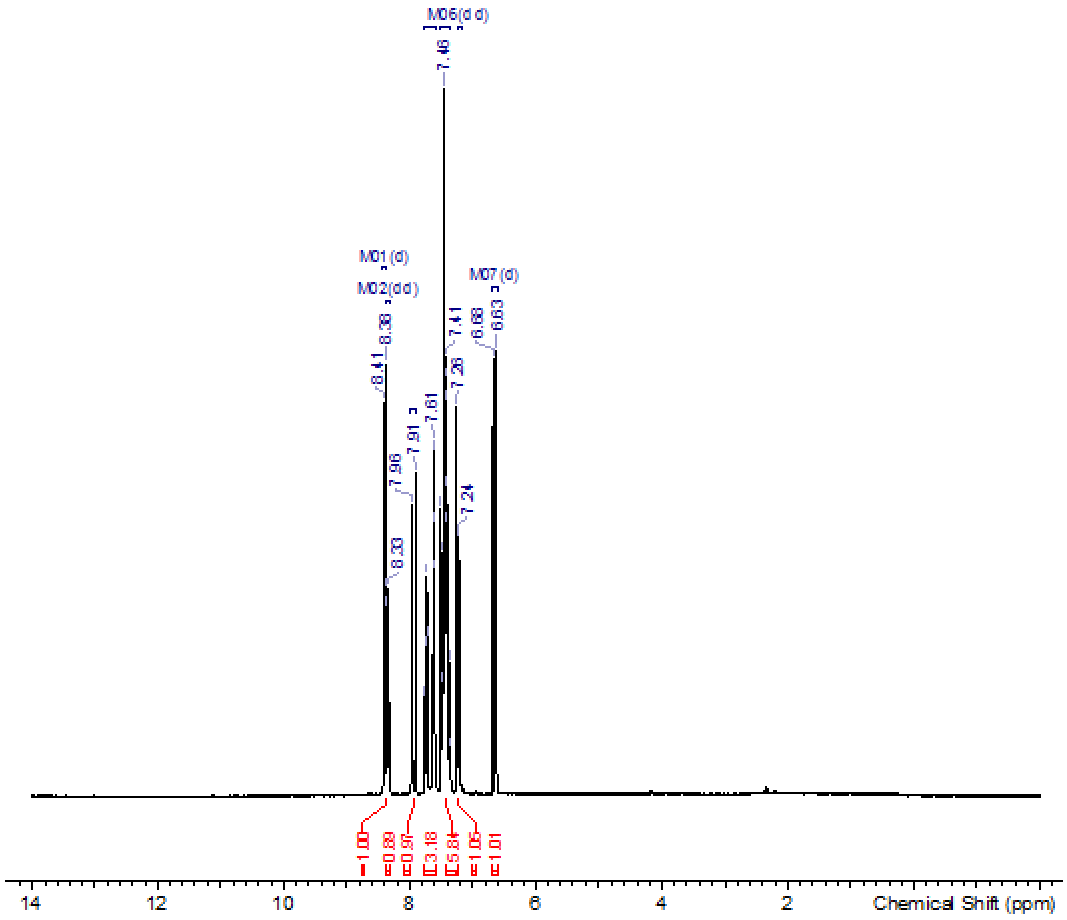
M06(d d)
7.46
M01 (d)
M02(dd)
8.41
8.38
8.33
7.96
7.91
7.61
7.41
7.28
7.24
M07 (d)
6.68
6.63
1.00
0.89
0.97
3.18
5.84
1.05
1.01
14
12
10
8
6
4
2
Chemical Shift (ppm)

compound 17

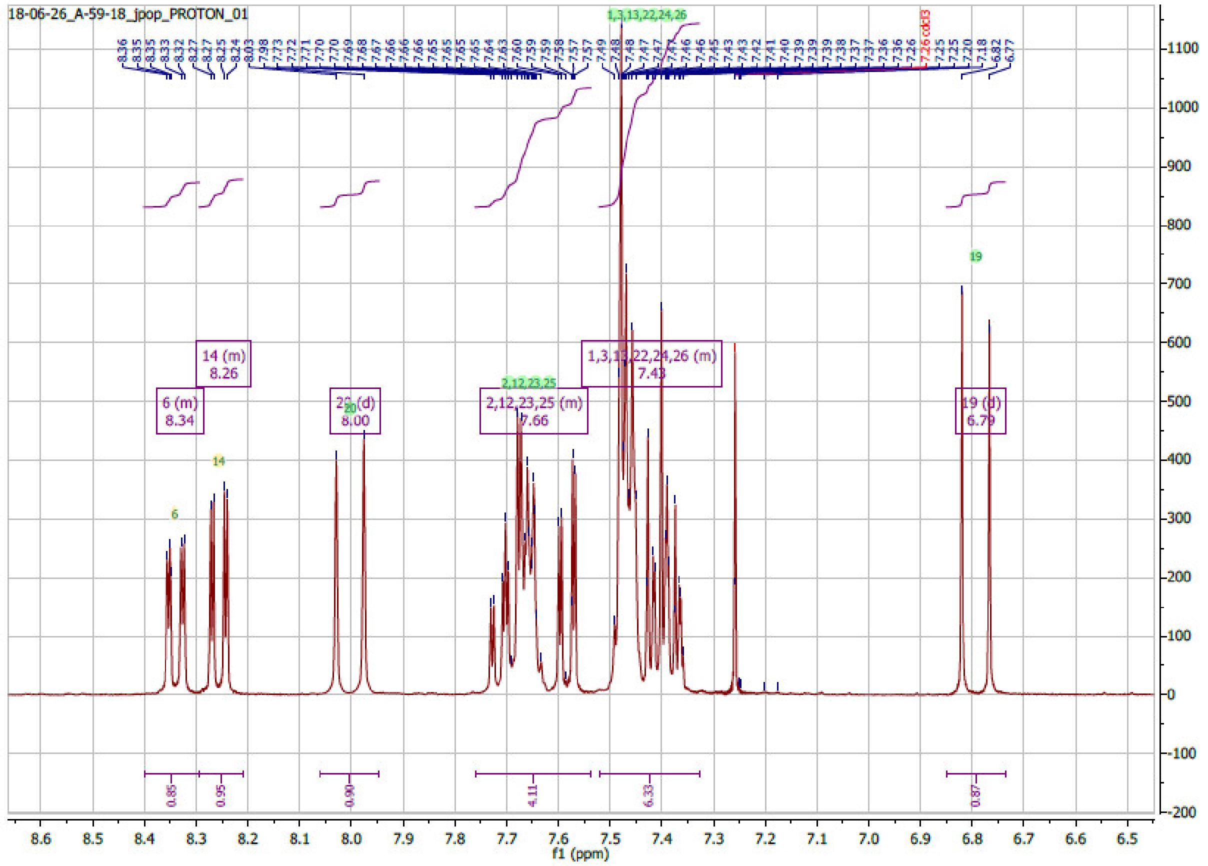

2. LC MS spectra

compound 1

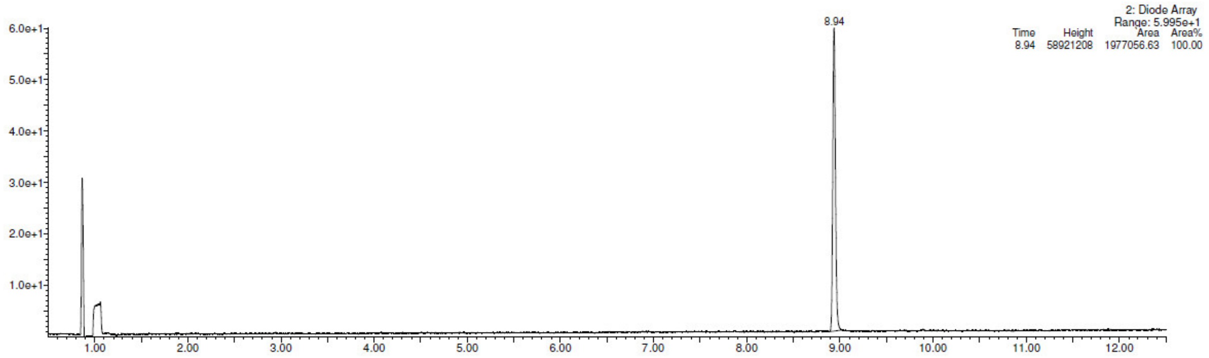

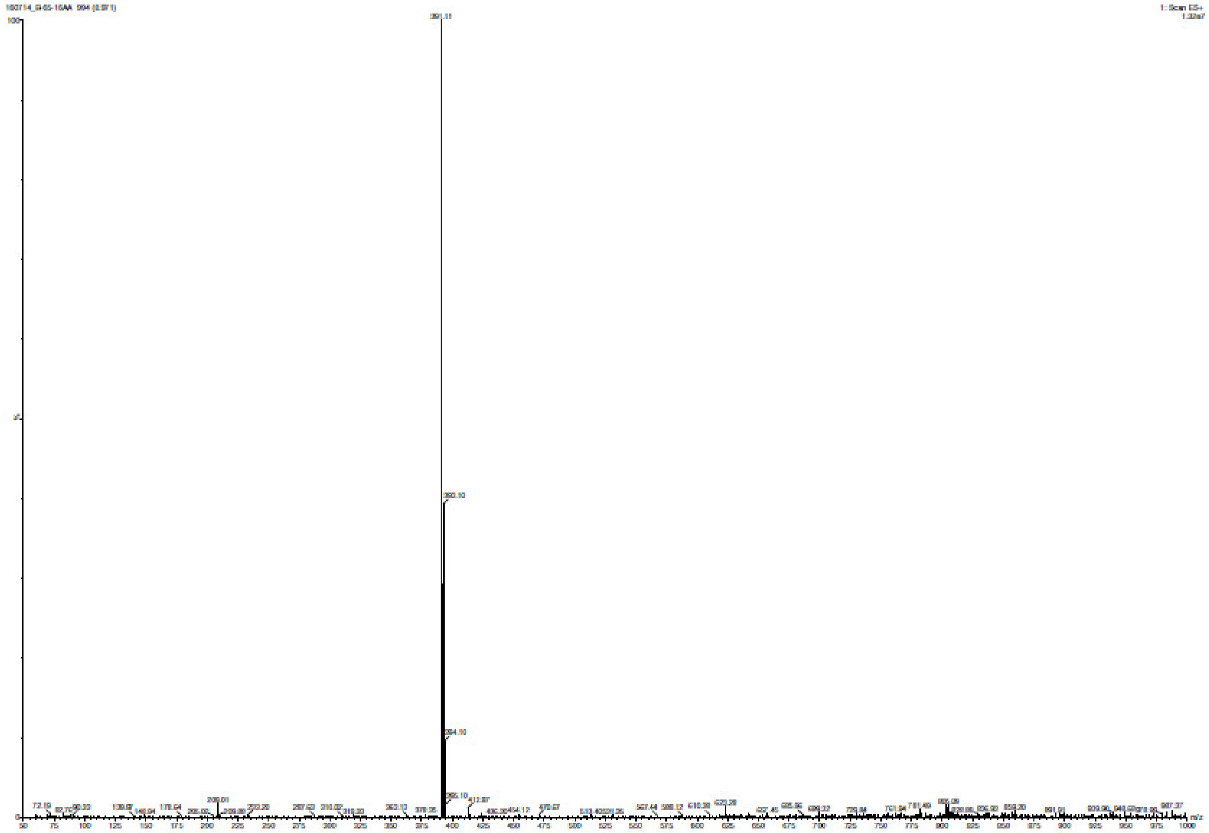

compound 2

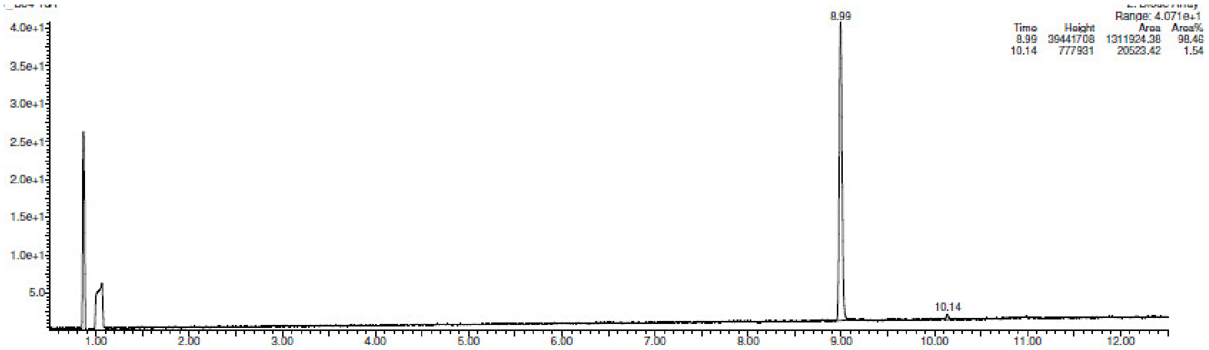

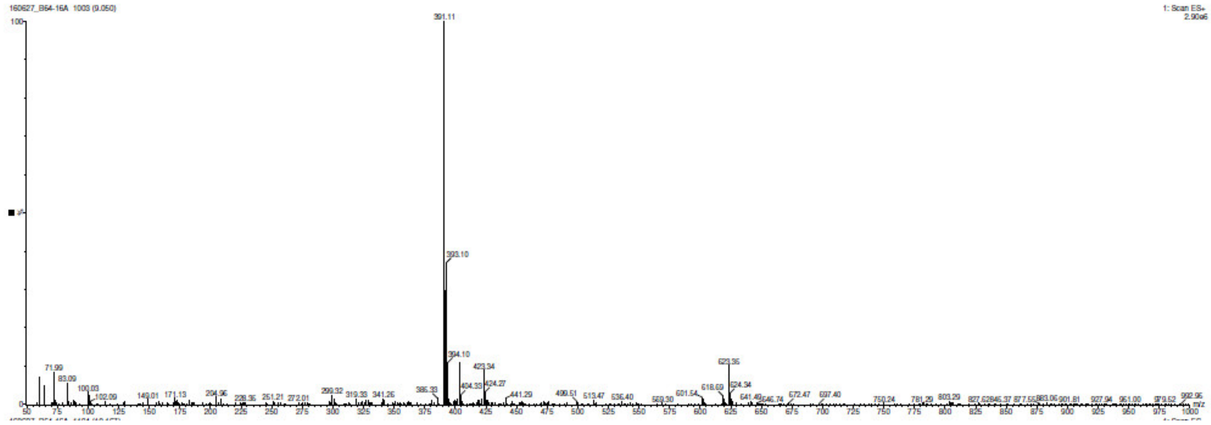

compound 3

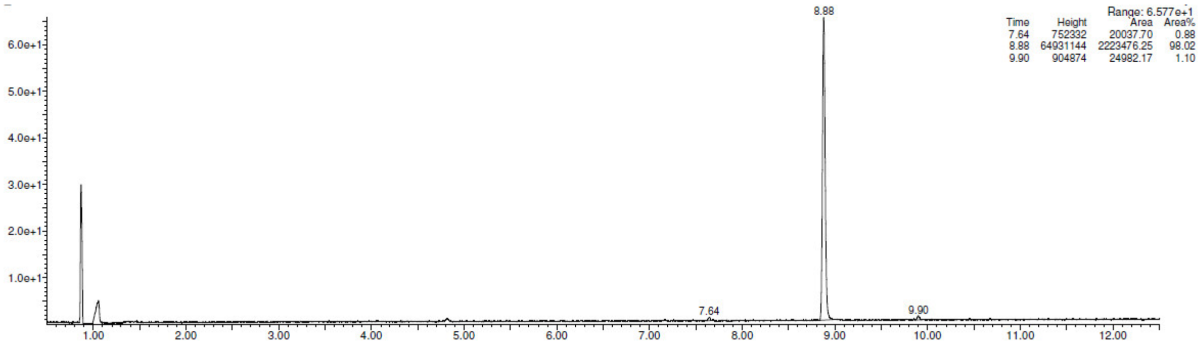
Range: 6.577e+1
Time Height Area Area%
7.64 752332 20037.70 0.88
8.88 64931144 2223476.25 98.02
9.90 904874 24982.17 1.10

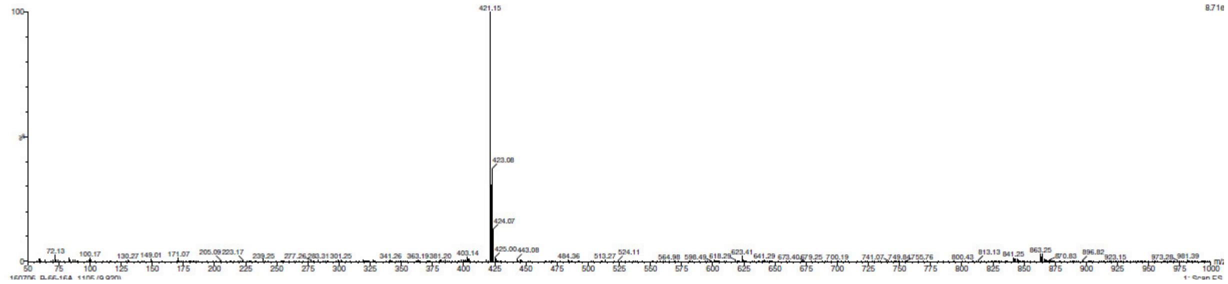

compound 4

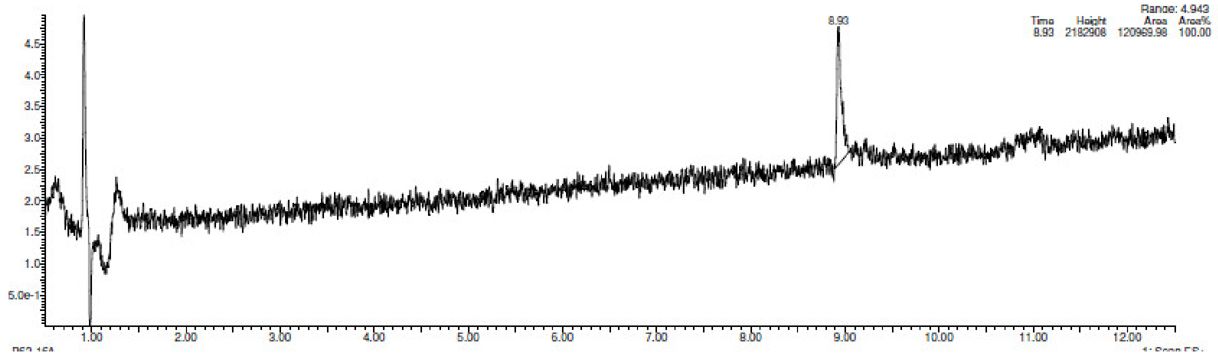
Range: 4.943
Time Height Area Area%
8.93 2182908 120969.98 100.00

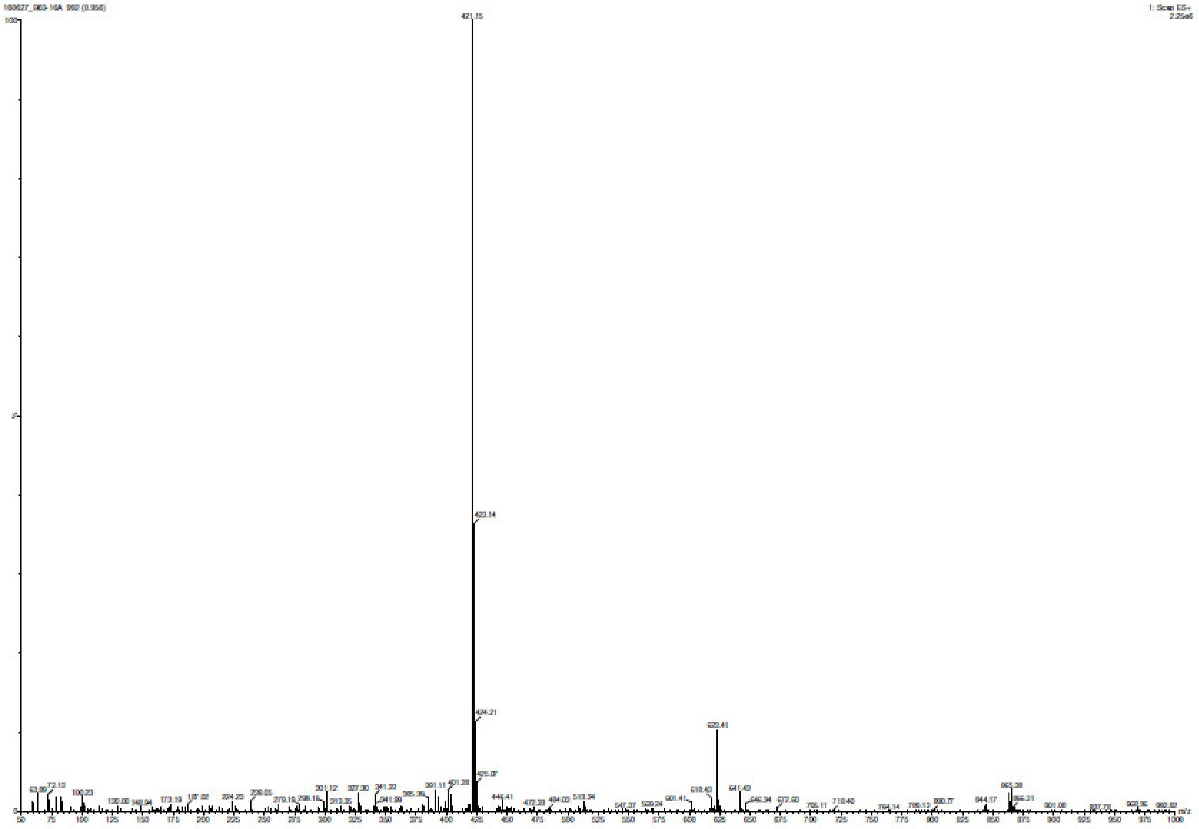
1: Scan ES+
2.25e6
421.15
423.14
424.21
425.07
623.41
m/z

compound 5

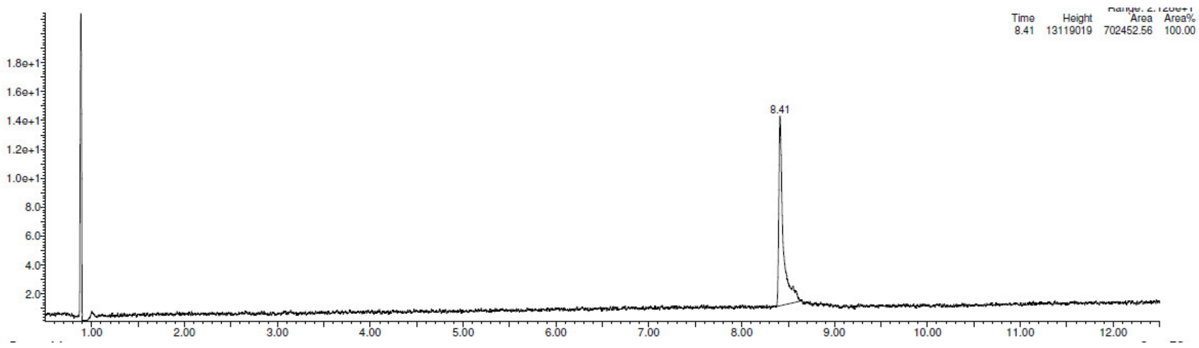
Time Height Area Area%
8.41 13119019 702452.56 100.00
8.41

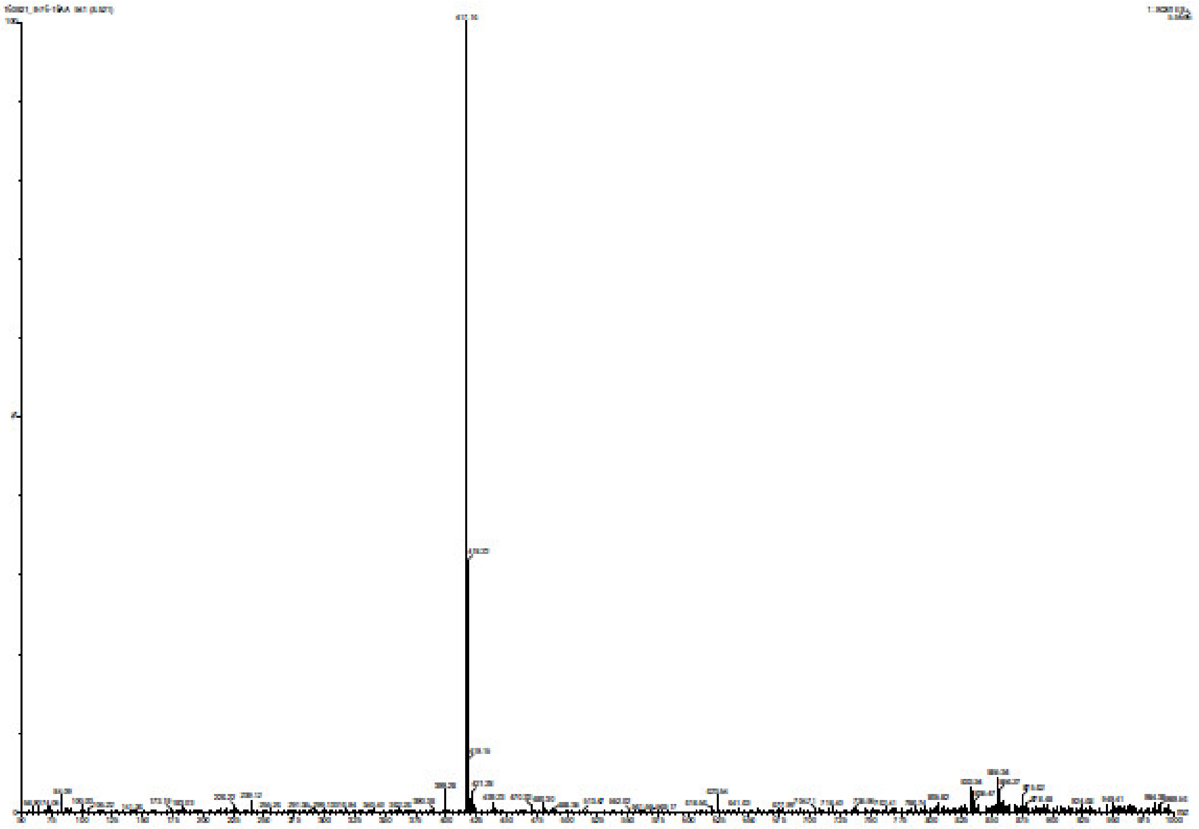

compound 6

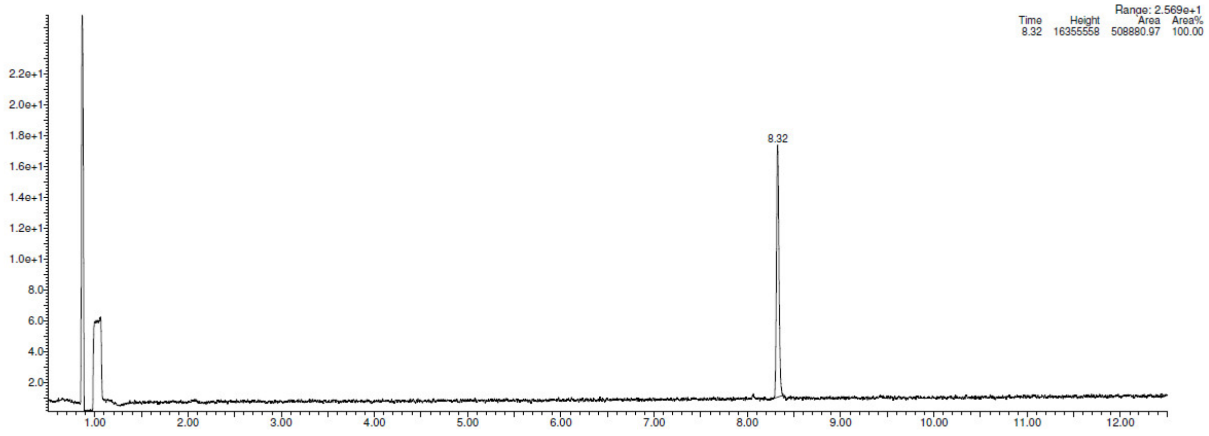
Range: 2.569e+1
Time Height Area Area%
8.32 16355558 508880.97 100.00
8.32
2.2e+1
2.0e+1
1.8e+1
1.6e+1
1.4e+1
1.2e+1
1.0e+1
8.0
6.0
4.0
2.0
1.00 2.00 3.00 4.00 5.00 6.00 7.00 8.00 9.00 10.00 11.00 12.00

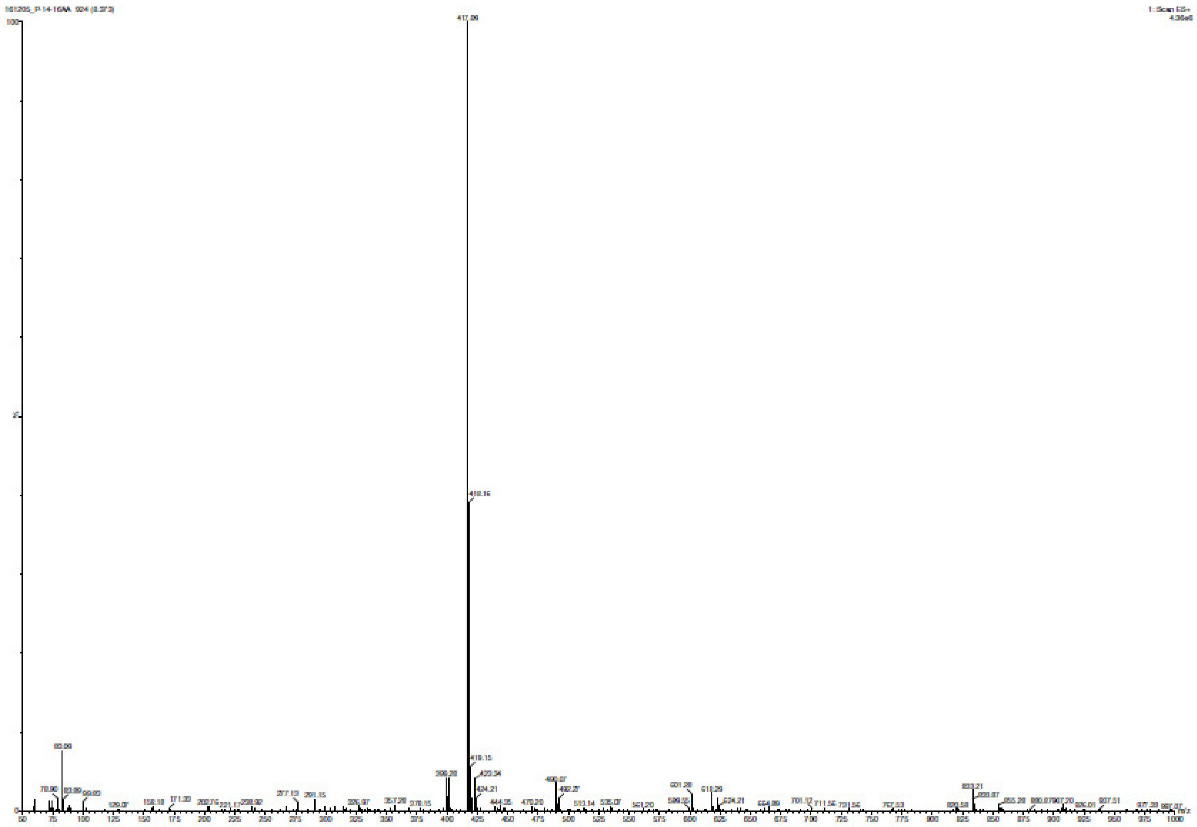

compound 7

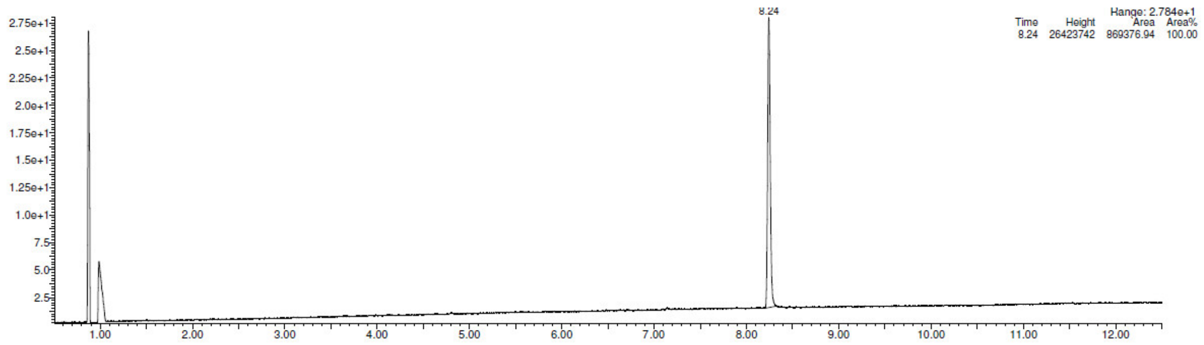
8.24
Range: 2.784e+1
Time Height Area Area%
8.24 26423742 869376.94 100.00
2.75e+1
2.5e+1
2.25e+1
2.0e+1
1.75e+1
1.5e+1
1.25e+1
1.0e+1
7.5
5.0
2.5
1.00
2.00
3.00
4.00
5.00
6.00
7.00
8.00
9.00
10.00
11.00
12.00

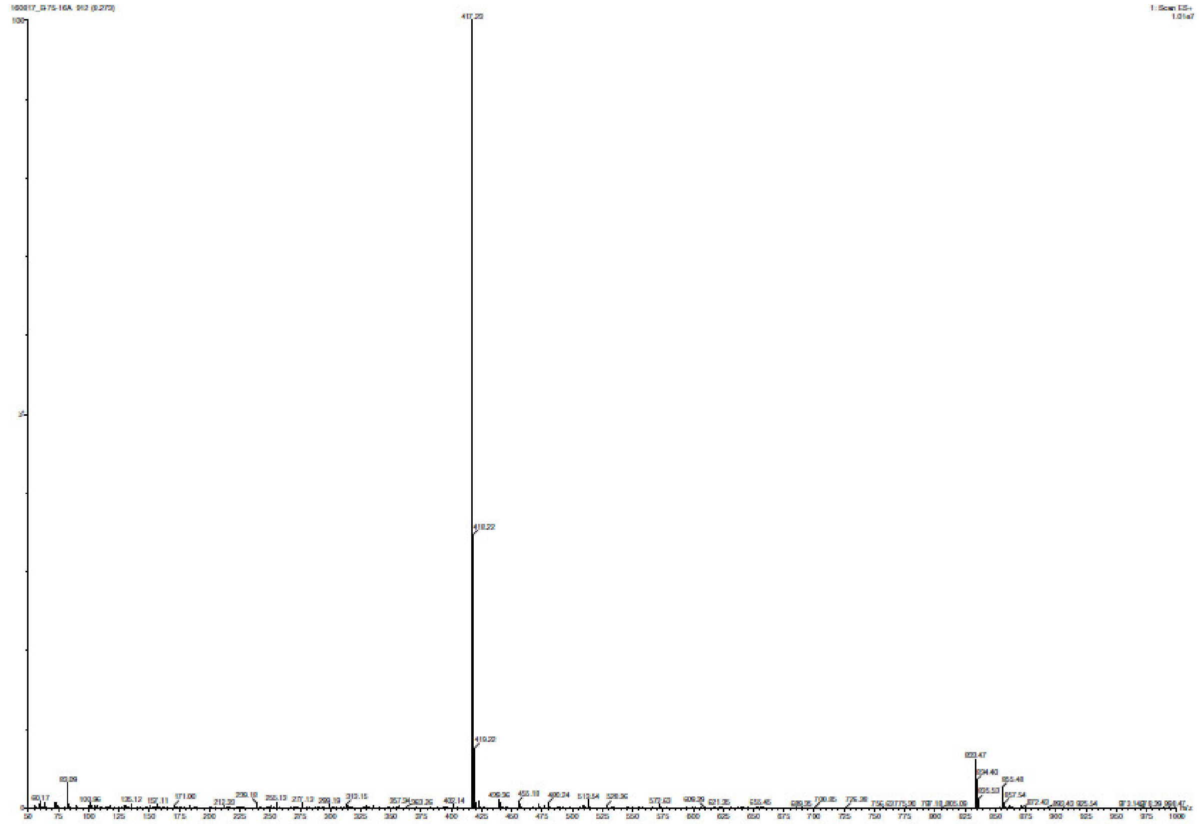

compound 8

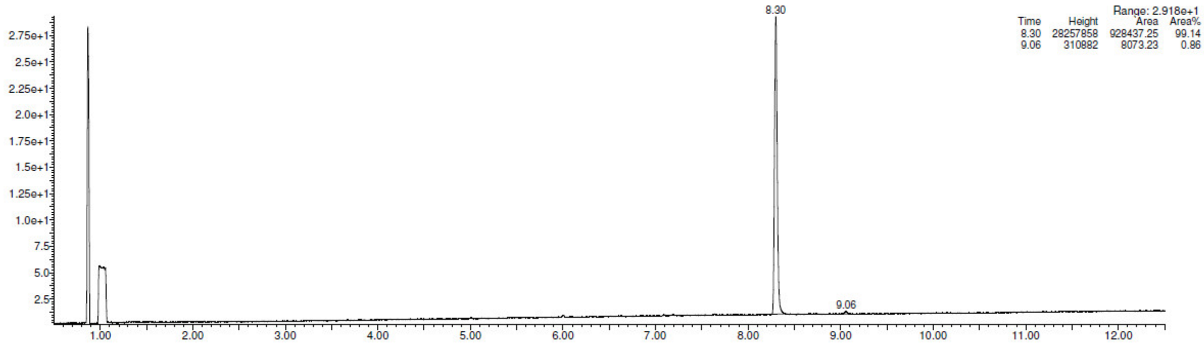

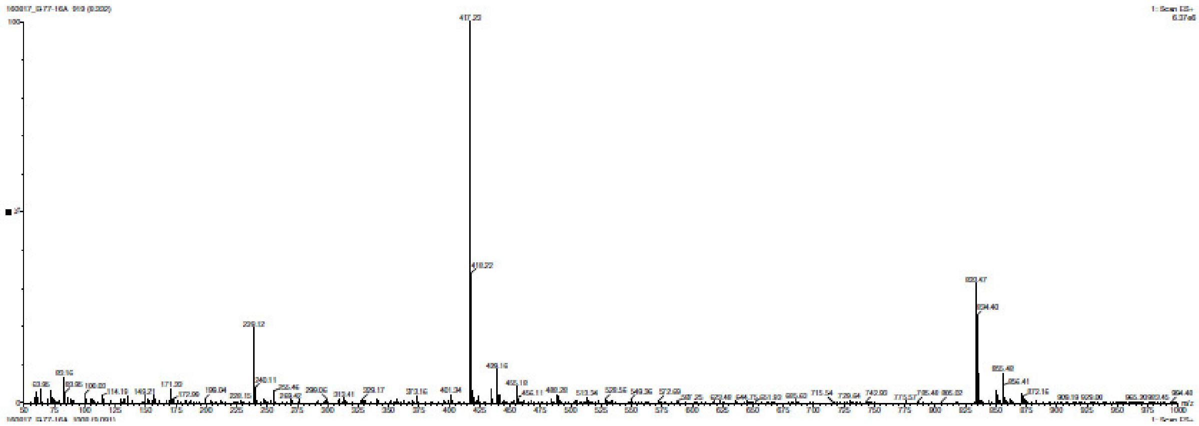

compound 9

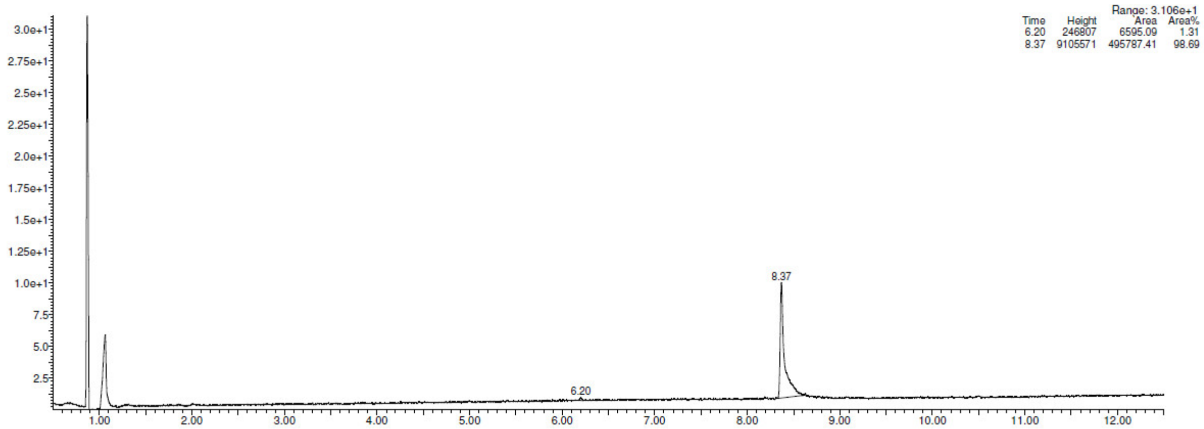
Range: 3.106e+1
Time Height Area Area%
6.20 246807 6595.09 1.31
8.37 9105571 495787.41 98.69

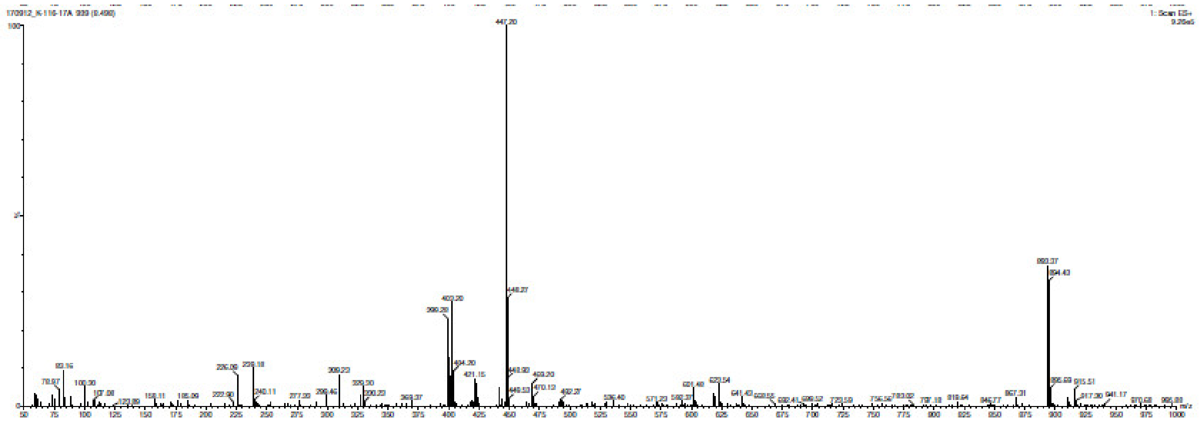

compound 10

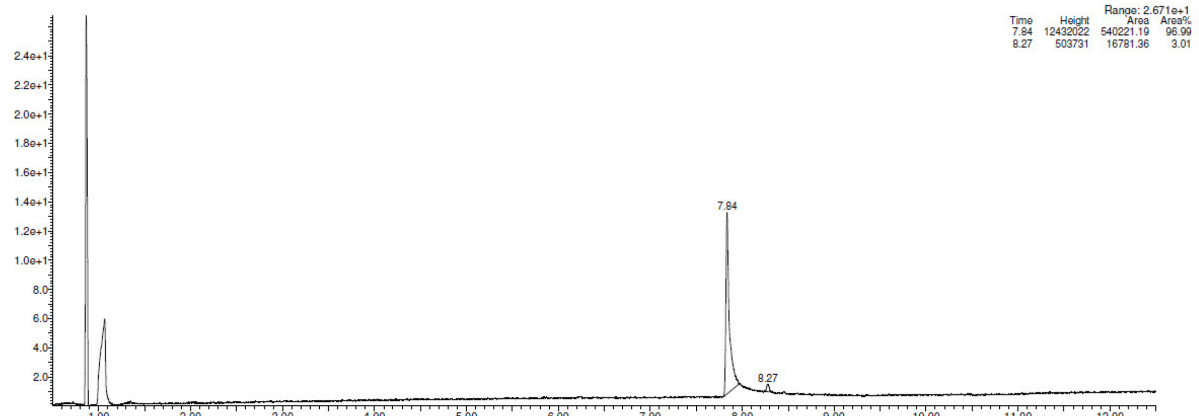
Range: 2.671e+1
Time Height Area Area%
7.84 12432022 540221.19 96.99
8.27 503731 16781.36 3.01

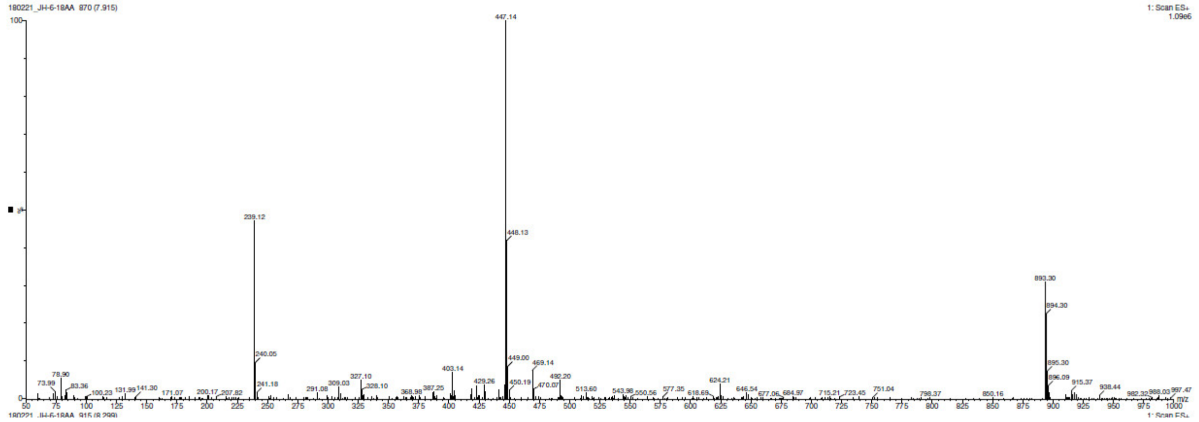

compound 11

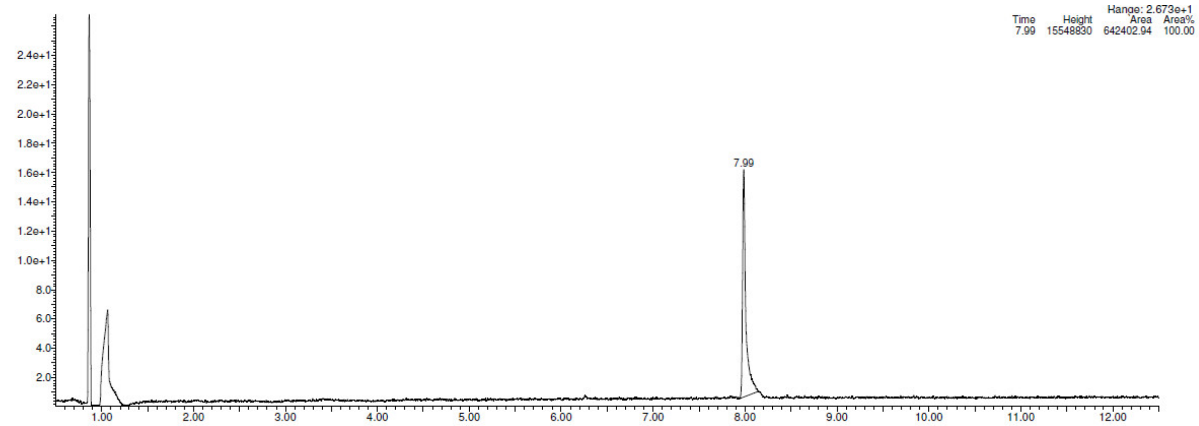

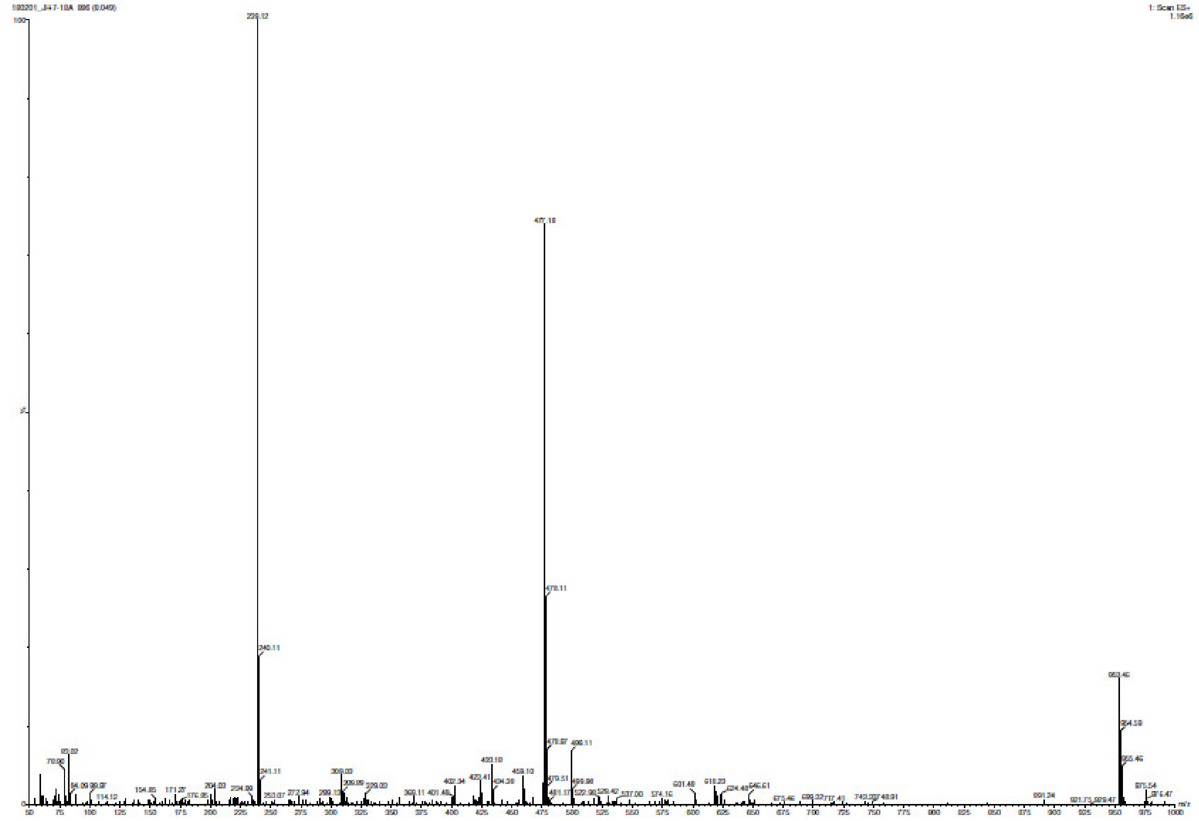

compound 12

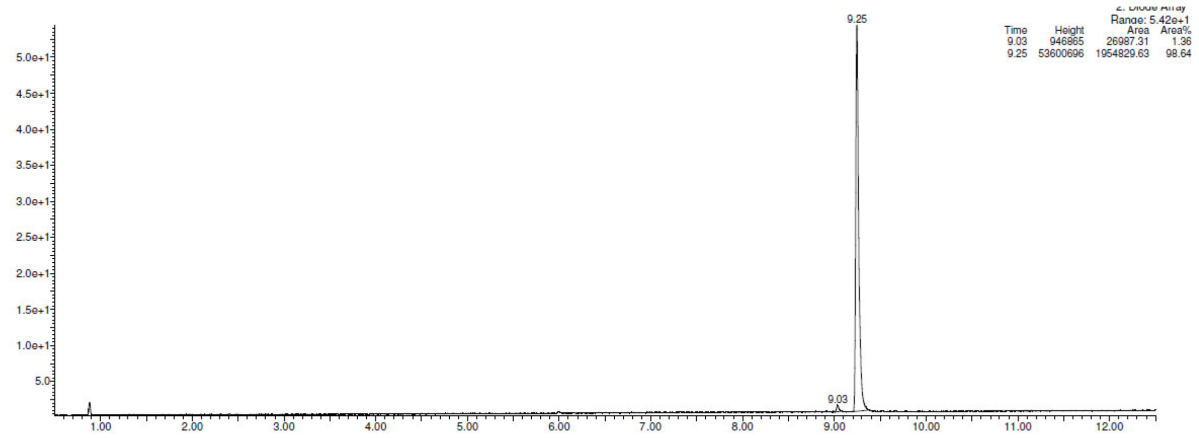

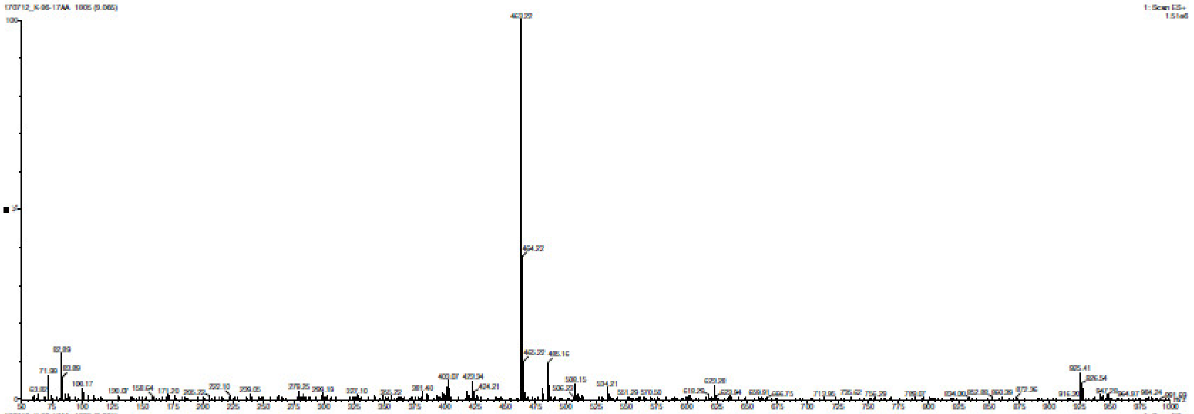

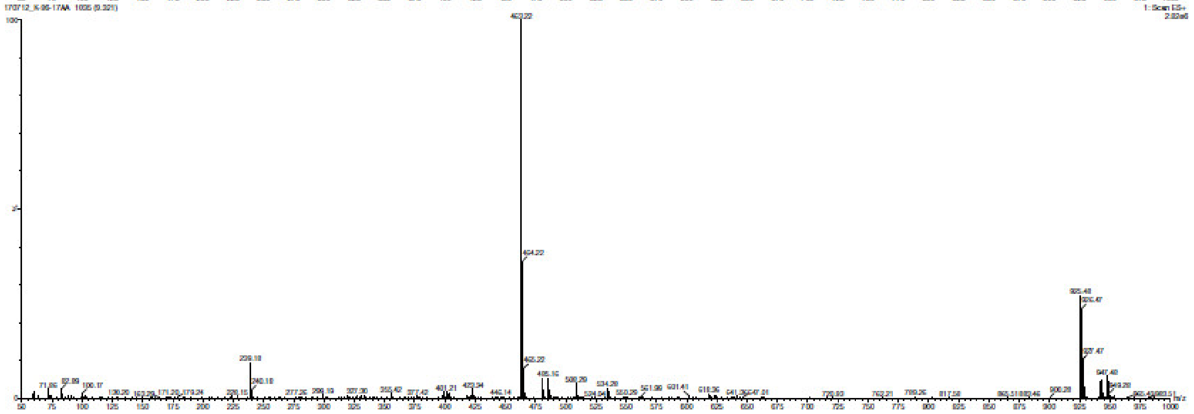

compound 13

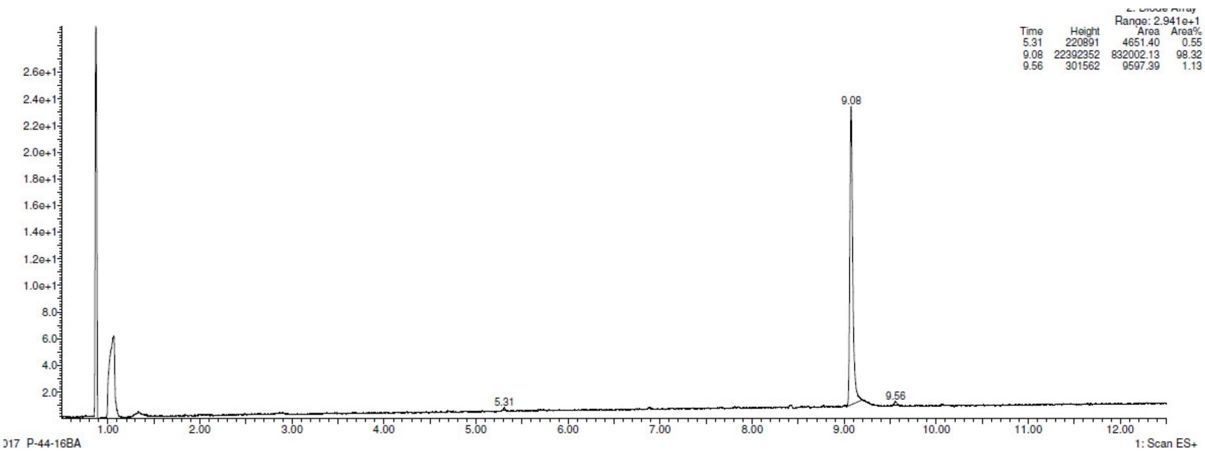
Range: 2.941e+1
Time Height Area Area%
5.31 220891 4651.40 0.55
9.08 22392352 832002.13 98.32
9.56 301562 9597.39 1.13
9.08
5.31
9.56
1: Scan ES+

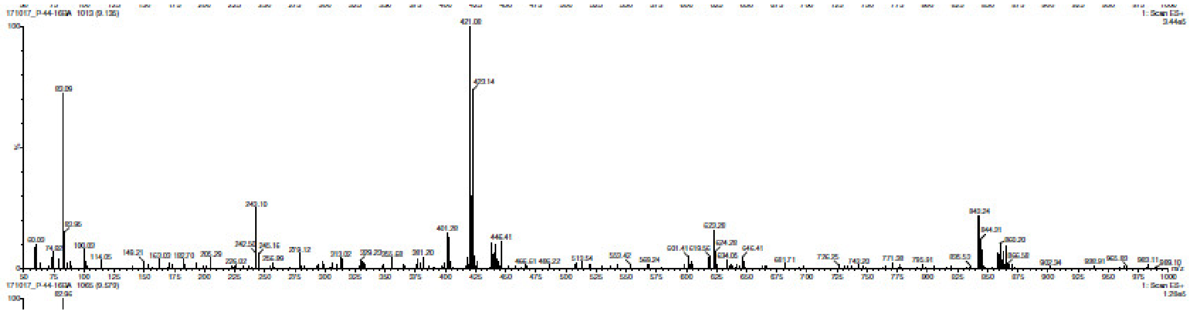
421.08
423.14
243.10
843.24

compound 14

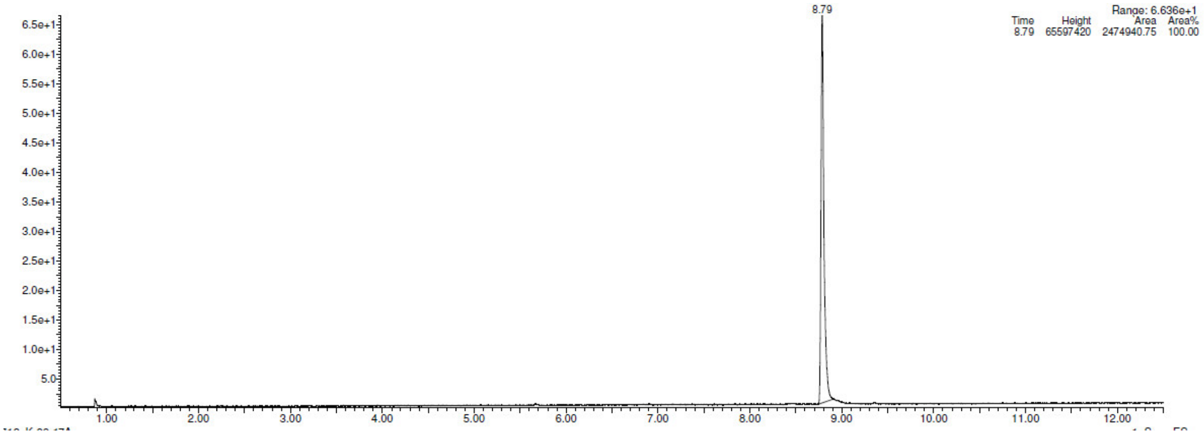
8.79
Range: 6.636e+1
Time Height Area Area%
8.79 65597420 2474940.75 100.00

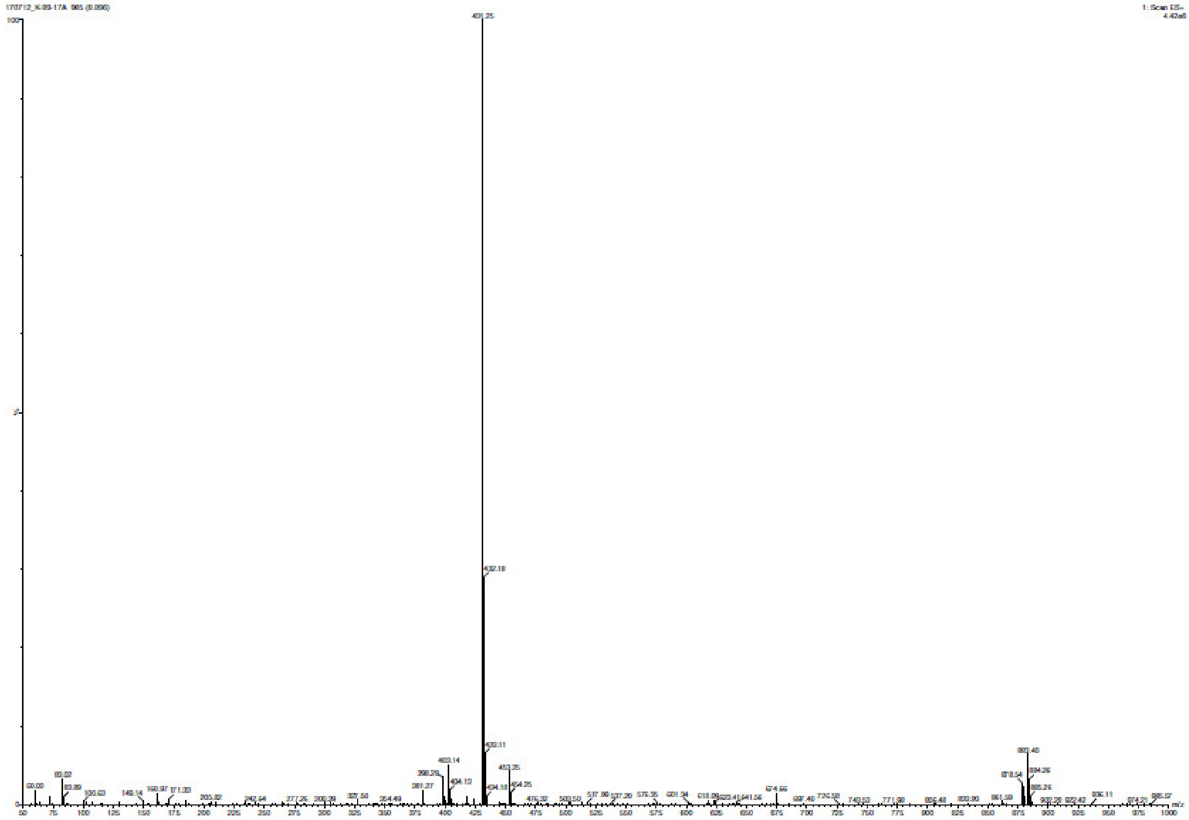

compound 15

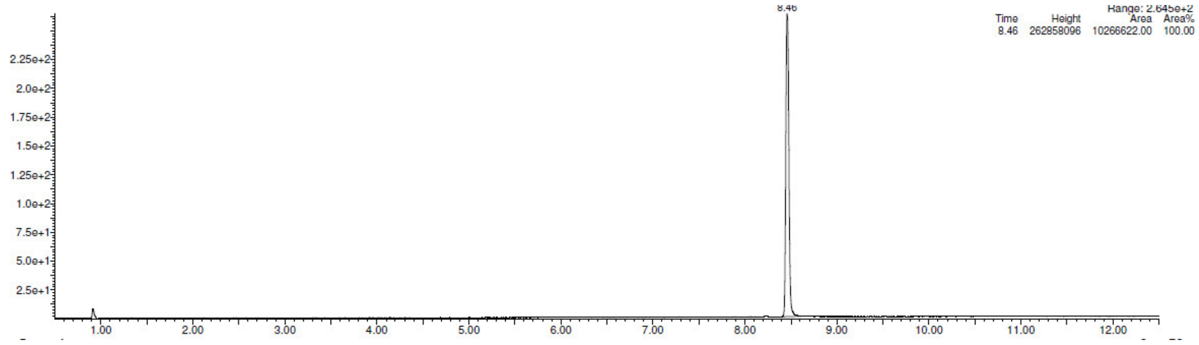
Range: 2.645e+2
Time Height Area Area%
8.46 262858096 10266622.00 100.00

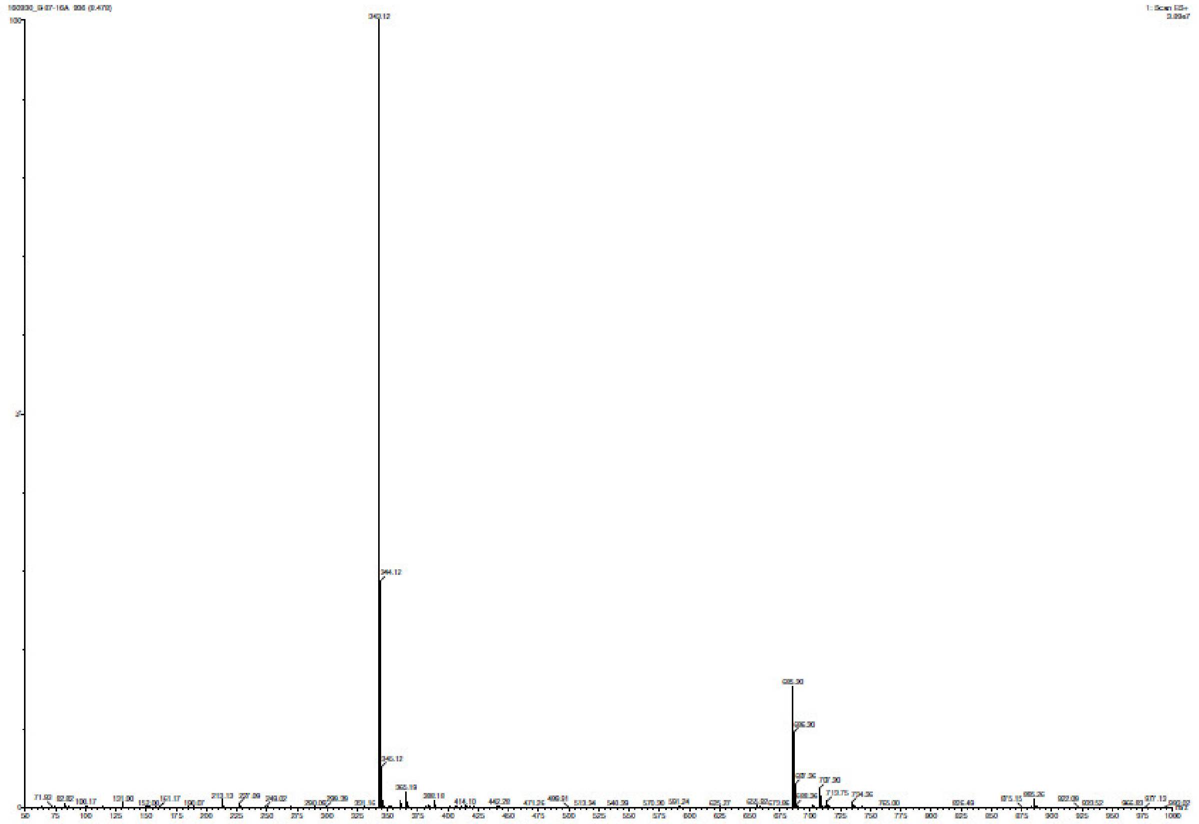
1: Scan ES+
343.12
344.12
345.12
685.30
686.30

compound 16

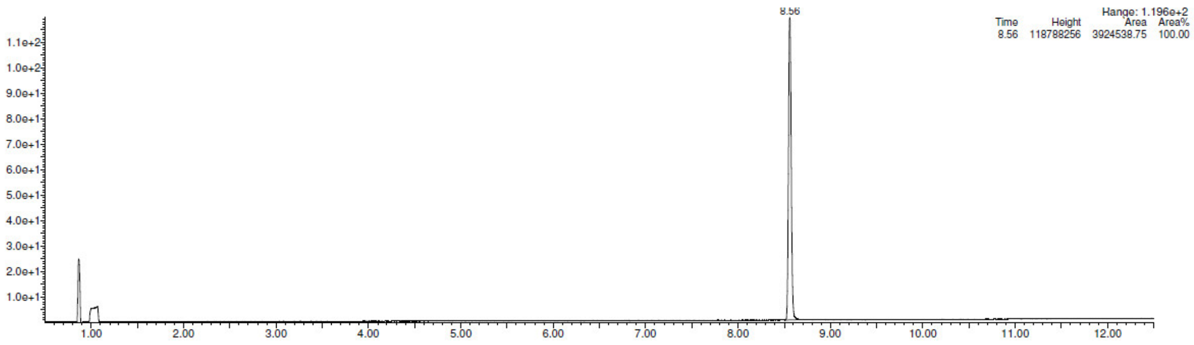
8.56
Range: 1.196e+2
Time Height Area Area%
8.56 118788256 3924538.75 100.00

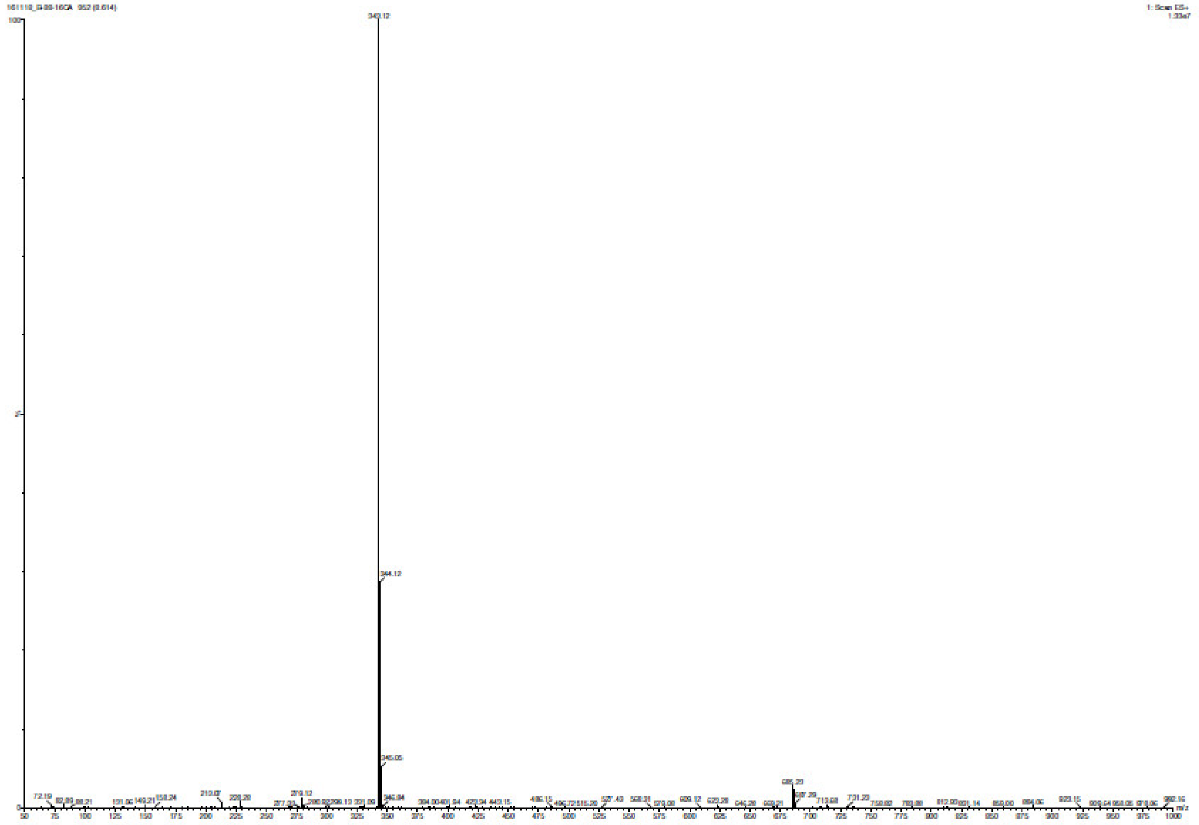
1: Scan ES+
343.12
344.12
345.05
m/z

compound 17

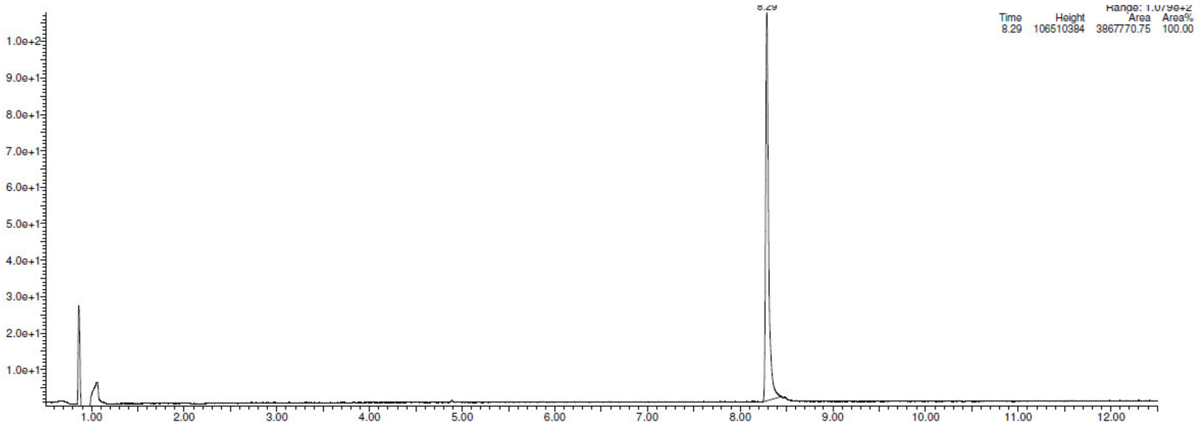
8.29
Time Height Area Area%
8.29 106510384 3867770.75 100.00

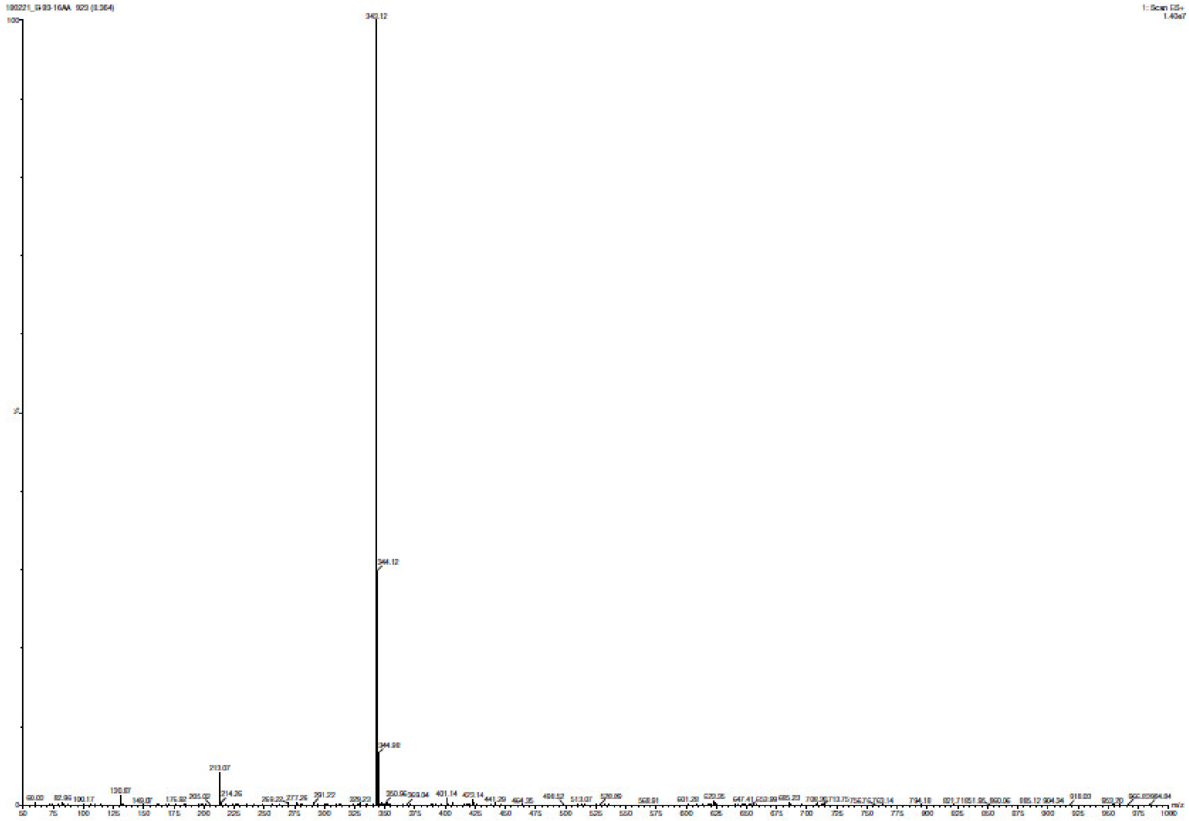
1: Scan ES+
344.12
213.07
m/z
